# Chemometric modeling of thermogravimetric data for the compositional analysis of forest biomass

**DOI:** 10.1371/journal.pone.0172999

**Published:** 2017-03-02

**Authors:** Gifty E. Acquah, Brian K. Via, Oladiran O. Fasina, Sushil Adhikari, Nedret Billor, Lori G. Eckhardt

**Affiliations:** 1 Forest Products Development Center, School of Forestry and Wildlife Sciences, Auburn University, Auburn, Alabama, United States of America; 2 Center for Bioenergy and Bioproducts, Department of Biosystems Engineering, Auburn University, Auburn, Alabama, United States of America; 3 Department of Mathematics and Statistics, Auburn University, Auburn, Alabama, United States of America; 4 Forest Health Dynamics Laboratory, School of Forestry and Wildlife Sciences, Auburn University, Auburn, Alabama, United States of America; USDA Forest Service, UNITED STATES

## Abstract

The objective of this study was to investigated the use of chemometric modeling of thermogravimetric (TG) data as an alternative approach to estimate the chemical and proximate (i.e. volatile matter, fixed carbon and ash contents) composition of lignocellulosic biomass. Since these properties affect the conversion pathway, processing costs, yield and / or quality of products, a capability to rapidly determine these for biomass feedstock entering the process stream will be useful in the success and efficiency of bioconversion technologies. The 38-minute long methodology developed in this study enabled the simultaneous prediction of both the chemical and proximate properties of forest-derived biomass from the same TG data. Conventionally, two separate experiments had to be conducted to obtain such information. In addition, the chemometric models constructed with normalized TG data outperformed models developed via the traditional deconvolution of TG data. PLS and PCR models were especially robust in predicting the volatile matter (R^2^–0.92; RPD– 3.58) and lignin (R^2^–0.82; RPD– 2.40) contents of the biomass. The application of chemometrics to TG data also made it possible to predict some monomeric sugars in this study. Elucidation of PC loadings obtained from chemometric models also provided some insights into the thermal decomposition behavior of the chemical constituents of lignocellulosic biomass. For instance, similar loadings were noted for volatile matter and cellulose, and for fixed carbon and lignin. The findings indicate that common latent variables are shared between these chemical and thermal reactivity properties. Results from this study buttresses literature that have reported that the less thermally stable polysaccharides are responsible for the yield of volatiles whereas the more recalcitrant lignin with its higher percentage of elementary carbon contributes to the yield of fixed carbon.

## Introduction

The use of lignocellulosic biomass for energy and to replace other products derived from fossil fuel will reduce net greenhouse gas emissions and persistent toxic materials that result during the extraction and processing of fossil fuels. Furthermore, because of its widespread distribution, biomass utilization can present an opportunity for localities to develop new and innovative industries [1]. Ligno-cellulosics may be herbaceous (annual) or non-herbaceous (perennial) and is made up of mainly cellulose, hemicellulose and lignin [2].

The chemical composition of lignocellulosic biomass to a large extent determines the optimal conversion methodology and also affects the distribution and yield of products. The chemical compositional distribution can be complex due to presence of needles, bark or woody tissues [3]. Because of the recalcitrance of lignin during biochemical conversion processes, lignocellulosic biomass is usually converted via thermochemical conversion methods such as combustion, gasification and pyrolysis [4]. When biomass is to be used as a source of energy or fuel, information about its proximate composition is necessary. Proximate analysis gives an indication of the thermal reactivity of biomass [5]. It is used to measure the mass fraction of water, volatile matter, ash and fixed carbon (by difference) in lignocellulosic biomass. Biomass with high volatile matter content are easier to ignite and yield higher quantities of liquid products; whereas a higher fixed carbon gives more solid products. Ash is formed from incombustible minerals in biomass, and is increased when material is contaminated with soil during harvesting. Apart from decreasing the available energy, ash content also influences the choice of conversion pathway, the overall cost of processing and also creates pollution concerns. The chemical and proximate characteristics of a fuel feedstock impact the kinetics of degradation, thus, the efficiency and emission parameters of a processing plant.

Consequently, prior knowledge of the chemical and proximate composition of the raw biomass feedstock will be useful in conversion processes. Considering the heterogeneity of biomass, efficient operation of a bioconversion plant will require real time adaption of process parameters to the characteristics of the feedstock. In addition, the ability to determine these properties using rapid and cost-effective techniques will be necessary in the successful commercialization of bio-based products.

One technique with this potential is thermogravimetric analysis (TGA). TGA is a rapid type of thermal analysis that measures the change in mass as a function of temperature as a material is heated at a fixed rate under a set of conditions. The mass loss gives insight into a sample’s chemical composition, thermal stability, number and sequence of reactions and kinetic parameters such as the order and activation energy of the chemical and physical reactions occurring [6,7]. TGA has been a useful tool for determining the thermal decomposition behavior of biomass and the kinetic parameters required for the design and operation of thermochemical conversion equipment. It has widely been used in the characterization of forestry residues [8], softwoods and hardwoods [9], corn stover [10] and municipal solid waste [11]. TGA was also utilized to study the degradation temperatures and kinetic parameters of several understory grasses found in a longleaf pine (*Pinus palustris*) ecosystem [12]. Employing TGA together with differential scanning calorimeter (DSC), Owen et al. 2015 [13] determined the rate, kinetics and energy involved in the thermal degradation of loblolly pine biomass in both air and nitrogen. Systems integrating TGA with other analytical tools such as Fourier transform infrared spectroscopy (FTIR), gas chromatography (GC) and mass spectroscopy (MS) have also been used to enable the identification and quantification of the composition and evolution rates of gaseous and liquid products during the pyrolysis and gasification of biomass feedstocks [14,15,16]. In recent times, a couple of studies have explored the application of TGA in the quantitative [17,18] and qualitative [19,20] characterization of lignocellulosic biomass. Several researchers have also reported the successful use of this tool to investigate the physicochemical changes that occur with the pretreatment of lignocellulosic biomass. For instance, Zhang et al. (2014) [21] used TGA to investigate the thermal stability / recalcitrance of biomass that have been subjected to ionic liquid (IL) pretreatment; whereas Singh et al. (2015) [22] used it to investigate the effect of different pretreatment methods (i.e. IL, ammonia fiber expansion and dilute sulfuric acid) on corn stover. Traditionally, researchers have determined the chemical composition of fuels by the deconvolution of derivative thermograms (DTGs), especially in quantitative analysis.

In this study, the objective was to employ chemometric modeling of thermogravimetric (TG) data as an alternative approach to estimate the chemical and proximate composition of forest-derived biomass. Chemometrics uses mathematical and statistical tools to extract pertinent information from chemical data [23]. It is hypothesized that, because the mass loss that occurs during TGA gives an indication of a material’s chemical composition, chemometrics can be applied to TG data (also known as thermograms) to determine and then predict the thermochemical properties of lignocellulosic biomass.

## Materials and methods

### Sample preparation

Biomass samples were obtained during harvesting operations on several *Pinus taeda* (loblolly pine) plantations in southern Alabama, USA. Biomass materials were sampled with permission from Corley Land and Timber, a private land owner. The stands were between 10 and 18 years old, and the diameter at breast height (dbh) of trees ranged from 10 cm to 20 cm. Materials acquired included the whole tree, wood and bark, slash and clean wood chips of loblolly pine. Material labeled as whole tree comprised the entire above ground biomass of loblolly pine trees. Clean wood was sampled from either whole debarked stems or from disks that were removed at 5 feet interval along the main stem. All the disks from a tree were combined into a single sample. Wood and bark material was sampled from the wood and bark of whole stems of southern pines (mostly loblolly pine), and slash material was the limbs and foliage of delimbed loblolly pine trees. Except for the debarked disks that were transported and chipped at Auburn University, AL, all other materials were sampled onsite from chip streams at chipper discharge. A sampling pipe was raised into a chip stream 8–10 times per load. Final representative subsamples were obtained in the laboratory through coning and quartering. Ten biomass sets were sampled for each of the four biomass types to give a total sample size of forty. Biomass used in this study is representative of feedstock material a bioprocessing plant located in this region will procure either as pre-commercial thinnings, whole tree from dedicated energy plantations, or pulpwood chips.

In preparation for analysis, chipped materials were first ground to pass a 1/8 in. screen of a hammer mill, followed by further grinding in a Wiley mill to pass a 40-mesh screen size to give a homogenous material. Test samples were stored in airtight plastic vials until time of analysis.

### Methods

#### Compositional analysis

Test materials were first extracted with industrial grade acetone for 6 hours in a Soxhlet Apparatus for extractive content determination. The major chemical constituents of forest biomass (i.e. hemicellulose, cellulose and lignin) were then determined via wet chemistry as specified in NREL LAP (2012) [24]. Air-dried extractive-free material was hydrolyzed with sulfuric acid in a two-step procedure.

The proximate composition was determined according to conventional standards. For ash determination, 1 g of unextracted test sample was weighed into a dry crucible and heated for 12 minutes at 105°C. The temperature was raised to 250°C, held isothermal for 30 minutes and then ramped up to 575°C. The final temperature was maintained for another 180 minutes. The ash content was computed as given in NREL LAP (2008) [25]. Volatile matter content of unextracted biomass samples were determined as specified in CEN 15148 (2005) [26] using a furnace (VMF Carbolite, model 10/6/3216P, England). Empty crucibles with their lids were first heated to 900°C ± 10°C for 7 minutes. After allowing to completely cool in a desiccator, they were filled with 1 g of test material, covered with lids and placed again in the furnace for 7 minutes. Unlike ash and volatile matter, the fixed carbon of biomass is a calculated value. It is the summation of the percentage of moisture, volatile matter and ash deducted from 100.

For each sample, experiments were run in duplicates. Analysis of Variance (ANOVA) followed by Tukey pairwise comparison tests between the four biomass types (α = 0.05) was performed using the R Stats Package. ANOVA was done to determine if differences existed in the property means for the four biomass types, and Tukey HSD tests conducted for pairwise comparison.

#### Thermal analysis

Thermal decomposition of biomass samples were done in a Pyris 1 TGA thermogravimetric analyzer (PerkinElmer, Waltham, MA, USA) using different heating cycles. For the proximate analysis, adopting an earlier study by Acquah (2010) [27] and the standard test method specified in ASTM E872-82 [28], 7 mg ± 2 mg of unextracted air dried samples ground to a homogenous powder (i.e. passed 40-mesh screen size) were used for the thermal analysis. This amount was enough to provide a good contact between the sample and the crucible, and also reduce the limitations associated with mass and heat transfer. In addition, approximately the same sample mass was used for each test run to ensure reproducibility and reduce the run to run variation [17,20,29]. A test sample was heated from 30°C to 105°C at a rate 20°C/min in an atmosphere of nitrogen. The temperature was held at 105°C for 5 minutes, later ramped up to 800°C at 50°C/min and then held isothermal for 7 minutes. Next, air was introduced and maintained at 800°C for an additional 10 minutes. The other method involved heating unextracted test samples from 30°C to 800°C at a rate of 20°C/min in the presence of nitrogen. Both methods took approximately 38 minutes.

The TGs of samples were exported into excel for initial analysis. Several studies have been conducted in which the derivative of TG data have been used to show how the major chemical components thermally degrade in inert atmosphere at different temperature ranges. The amorphous, branched and lower molecular mass hemicelluloses is the first to decompose at mild temperatures (150°C to 360°C), followed by the linear, higher molecular mass cellulose (250°C to 440°C). The thermal degradation of lignin, a complex 3-dimensional polymer has been reported to occur over a wider range; from as low as 180°C, or high as 300°C, to 900°C [14,17,30,31,32]. The mass loss from room temperature to about 180°C has been attributed to the loss of water and lower molecular mass volatiles. Several studies in recent times have also sought to determine the proximate composition (i.e. volatile matter, fixed carbon and ash contents) of biomass from thermogravimetric data [18,27,32].

Based on the literature, two degradation regimes were adopted to be used for the quantitative computation of hemicelluloses, cellulose and lignin. The following equations were employed:

%  Hemicelluloses={(B−C)÷A}*100 (1)

%  Cellulose={(C−D)÷A}*100 (2)

%  Lignin={D÷A}*100 (3)

For the first regime [30] which will be known as KIN-1 henceforth, A is the mass of test sample remaining after 130°C, B is the mass after 250°C, C is the mass after 350°C and D is the mass after 500°C. For the second regime [14] dubbed KIN-2, A is the mass after 180°C, B is the mass after 360°C, C is the mass after 440°C and D is the sample mass after 440°C.

Similarly, a degradation regime (i.e. KIN-3) was adopted [27] and used to calculate the amount of volatile matter, fixed carbon and ash using TG data. The computations were as follows:

%  Volatile matter={(A−B)÷A}*100 (4)

%  Ash={C÷A}*100 (5)

%   Fixed carbon=100−{%  Volatile matter+% Ash } (6)

Where A is the dry mass of test sample; B is mass at 600°C after holding for 7 minutes in the presence of nitrogen and C is the residual mass after complete oxidation in air.

Simple linear regression models were then developed to evaluate how these estimated properties compared to values experimentally obtained.

#### Chemometric modeling

Chemometrics involve the application of multivariate analysis (MVA) to chemistry-relevant data. It has been used to determine the concentration of compounds in mixtures, identify substructures in unknown chemical compounds and predict their properties. MVA uses many measured variables (X_1_, X_2_…..X_i_) simultaneously to quantify a response or target variable (Y) [33]. In this case, X is thermogravimetric data and Y is the measured property. The PROC PLS package in SAS (SAS Institute, Inc. Cary, NC, USA) was used to develop both principal component regression (PCR) and partial least squares regression (PLS) models.

PCR is a two-step process involving principal component analysis (PCA) and multiple linear regression (MLR). PCA reduces the dimensionality of a dataset by taking a set of correlated X variables and transforming them into a smaller set of uncorrelated variables known as principal components (PC) scores. In other words, assuming that there are n observations X_ij_ on p correlated variables X_1_, X_2_,…,X_p_, i = 1,…,n, j = 1,…, p, PCA finds new uncorrelated Z_1_, Z_2_,…,Zp that are linear combinations of X_1_, X_2_, …,X_p_ as

$${\text{Z}}_{\text{i}}={\text{ e}}_{\text{i1}}{\text{X}}_{\text{1i}}+{\text{ e}}_{\text{i2}}{\text{X}}_{\text{2i}}+\;\ldots \ldots .+{\text{ e}}_{\text{ip}}{\text{X}}_{\text{pi}}\;\&\text{ Var}\left({\text{Z}}_{\text{i}}\right)=\lambda_{\text{i}},\text{ i}=1,\ldots ,\text{p}$$

where λ_i_s (λ_1_ >λ_2_ >…>λ_p_) and **e**_i_ are the eigenvalues and the corresponding eigenvectors of the covariance matrix of data matrix X (n by p). The coefficient, e_ij_ is a measure of the relevance of the j^th^ original variable to the i^th^ PC irrespective of the other variables. The coefficients, which are also known as eigenvectors or component loadings are proportional to the correlation between Zs and Xs; thus they can be used in the elucidation and interpretation of models. The values of the i^th^ principal component are called the PC scores (i.e. Zs). PCR then regress the PC scores against a response variable, Y.

The model structure for PLS is similar to that of PCR. However, unlike PCR, PLS takes the Y variable into consideration and generate latent variables scores (LVs, synonymous to PCs in PCA) in such a way that the covariance between X and Y is maximized.

Before exporting the TG data into SAS (SAS Institute, Inc. Cary, NC, USA) for model construction, they were normalized to the dry mass (i.e. the temperature range associated with the loss of water: 30°C to 105°C were excluded), S1 File. A total of forty samples (i.e. 10 of each biomass type) were used in the calibration of chemometric models. Due to the relatively small sample size, a leave-one-out cross validation was used to validate the models [34]. With this technique, all available samples were utilized in validation as independent single-element test datasets. The PROC PLS procedure, using either the NIPALS (i.e. non-iterative PLS) or PCR algorithm gave the optimum models as those with the absolute minimum predicted residual sum of squares (PRESS). The final optimum models were chosen as those that used a lesser number of LVs or PCs to give a PRESS value that was not statistically different (Hotelling’s T^2^_;_ p > 0.1) from the lowest PRESS value achieved with a higher number of LVs or PCs. The predictive performances of models were also assessed with the standard error of cross-validation (SECV), coefficient of determination (R^2^) and ratio of preformance to deviation (RPD).

The chemical and proximate composition of forest residue used in this study is presented in Table 1.

**Table 1:** Properties of loblolly pine biomass.

	Lignin	Cellulose	Hemi-celluloses	Ash	Volatile matter	Fixed carbon
Whole	37.3 (1.6) a	31.0 (2.4) a	24.1 (2.2) a	1.8 (0.7) a	81.4 (1.4) a	9.8 (1.2) a
Wood & bark	35.9 (2.0) a	38.9 (3.8) b	22.8 (2.8) ab	1.5 (1.6) a	82.3 (0.8) a	9.7 (1.4) a
Slash	43.7 (1.7) b	25.2 (2.4) c	22.1 (4.2) ab	1.9 (0.2) a	77.3 (0.6) b	16.2 (0.8) b
Wood	33.5 (1.6) c	42.7 (2.4) d	20.3 (0.9) b	0.4 (0.1) b	84.7 (0.5) c	8.9 (0.7) a

Note: Mean values (SD in bracket) expressed on % oven-dry basis. N = 10 for each biomass type. Statistically different [Tukey Test, P<0.05] properties noted with different letters.

## Results and discussion

### Thermogravimetric analysis

**Fig 1:**
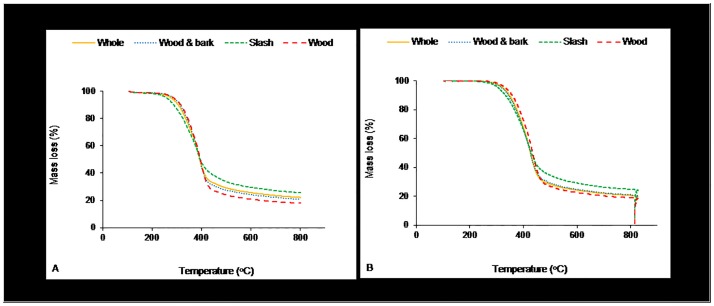
Mass loss from thermal decomposition of forest biomass in (A) nitrogen and (B) nitrogen plus air.

Fig 1 shows representative TGs obtained for each biomass type when volatilized using the two temperature programs. The thermograms are an average (n = 10) for each of the four biomass types. In Fig 1A, the TGs follow the characteristic thermal behavior of lignocellulosic biomass in the presence of nitrogen [8,13,17,20]. At about 150°C, the devolatilization process starts, causing mass loss. The decomposition is steep until about 450°C, after which the rate is more gradual. Maximum mass loss occurs approximately between 300°C and 450°C. The pyrolysis process removed 70% to 80% of the dry mass of forest biomass. The residual mass decreased drastically when air was introduced to facilitate combustion, Fig 1B. Under both pyrolytic and oxidative environments, wood had the least residual mass whereas slash had the highest. These results could be attributed to slash having a significantly higher percentage of the more thermally stable lignin and incombustible inorganics; while wood had the least, Table 1. The relatively more similar chemical and proximate composition of whole and wood and bark were made evident in their overlapping thermograms.

The run to run variation among all four biomass types used in this study was about 1% weight loss difference (with a range of 0.5% to 1.3%) along the temperature regime of 105°C to 800°C. As was to be expected, smaller variations were noted within the clean wood samples compared with the other biomass types under both pyrolytic and pyrolytic plus oxidative conditions. The whole tree samples were expected to exhibit a relatively larger within group variation compared to the other biomass types because of its more heterogeneous plant part composition of wood and bark from stems and branches, as well as foliage. This was however not the case in this study. The relatively small errors recorded could be attributed to the steps that were taken during material preparation to ensure representative and homogenous test samples.

**Fig 2:**
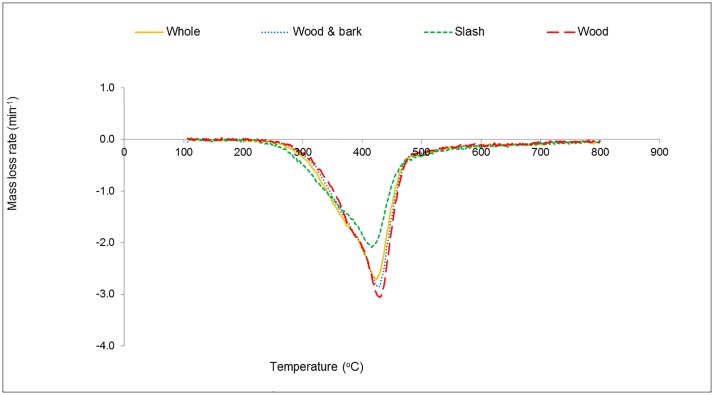
DTG curves of pine biomass in nitrogen plus air.

A DTG curve is a plot of the rate at which mass changes within a time range versus temperature [35]. It can be used to determine the point at which mass loss is most apparent. From to the DTG curves in Fig 2, the highest rate of mass loss occurred at a slightly higher temperature for whole, wood & bark and wood (i.e. 420°C) than it was for slash (400°C). This behavior of slash could be as a result of the significantly higher concentration of easily volatilized amorphous extractives compared to the other biomass types (i.e. whole = 4%, wood & bark = 2%, slash = 10%, wood = 3%) [36]. The bulk of mass loss happened from 150°C to 550°C as portrayed in the negative peak of the DTG.

According to literature, this peak results from the overlap of the degradation of hemicellulose and celluloses. The presence of acetyl groups in the amorphous and branched hemicelluloses contribute to their relatively low thermal stability [17,18,31]. The smaller shoulder peak at (350°C) attributed to the decomposition of hemicelluloses was not as prominent in whole, wood & bark and slash, as it was in wood, and other studies reported in the literature. When a similar occurrence was encountered by [13], the researchers attributed it to higher ash content of the samples. Degradation of lignin has been reported to occur over a wider temperature range, with significant mass loss occurring after 550°C. This gives the flat tail in the DTG of lignocellulosic biomass, Fig 2. There was a general upward shift in the temperature ranges that have been reported to correspond to the degradation of the major chemical constituents of lignocellulosic biomass. This could be a consequence of the relatively high lignin content of samples used in this study, Table 1.

### Chemometric modeling for property prediction

Normalized TGs were used in the construction of PLS and PCR models. The PCR algorithm generally utilized more PCs to obtain the absolute minimum PRESS because the Y variables were not considered in the computations of PCs, Table 2. Considering parsimony, the final optimum models for prediction were chosen as those that used fewer LVs/PCs to give a PRESS value that was statistically the same as the absolute minimum PRESS; usually only two or three LVs/PCs could accomplish this. In the modeling of volatile matter, the minimizing number of factors and the smallest number of factors with p > 0.1 were the same. Once the final optimum LVs/PCs were chosen, both the PLS and PCR algorithms gave similar SEC and PRESS values, Table 2. This gave the assurance that the most stable and robust models had been selected.

**Table 2:** Calibration statistics of TG-based chemometric models.

	PLS				PCR			
	SEC	LVs (Opt)	LVs (Sig)	Press (Sig)	SEC	PCs (Opt)	PCs (Sig)	Press (Sig)
Lignin	1.63	6	2	0.48	1.63	10	2	0.48
Cellulose	3.44	3	2	0.58	3.45	4	2	0.58
Volatile matter	0.78	2	2	0.31	0.78	2	2	0.31
Fixed carbon	1.33	7	3	0.58	1.22	7	5	0.54
Ash	0.49	3	2	0.86	0.51	3	3	0.83

The predictive statistics of the constructed models are presented in Table 3. The PLS models performed slightly better in predicting the understudied properties than PCR models because LVs are extracted in a way that optimally explains the variation in both predictor and response variables [23,33]. The R^2^—which measures the degree of linear association between measured and predicted—for cross-validated models ranged from a low of 0.32 (ash) to a high of 0.92 (volatile matter). Also, the RPD—which gives an indication of the predictive adequacy of a model—of models were from 0.59 (ash) to 3.58 (volatile matter). In the literature, a chemometric model with an R^2^ greater than 0.5 could be used in several applications ranging in sensitivity; from rough screening to quality assurance [37]. Likewise, a model that has an RPD of 1.5 or greater can be employed for preliminary prediction and screening [38].

**Table 3:** Predictive performance of TG-based chemometric models.

	PLS			PCR		
	SECV	R^2^	RPD	SECV	R^2^	RPD
Lignin	1.76	0.82	2.37	1.76	0.82	2.37
Cellulose	4.01	0.70	1.85	4.02	0.70	1.85
Volatile matter	0.79	0.92	3.58	0.79	0.92	3.58
Fixed carbon	1.47	0.78	2.14	1.32	0.82	2.39
Ash	1.39	0.32	0.59	0.82	0.37	1.28

Note: Models developed with the smallest number of LVs/PCs that gave PRESS values statistically not different form the absolute minim PRESS.

For the major chemical constituent, TG-based models were especially able to predict the lignin content of forest biomass (R^2^–0.82; RPD– 2.37 for both PLS and PCR), Table 3. The hemicelluloses were however very poorly modeled when chemometrics was applied to TG data. Nonetheless, Carrier et al. (2011) [17] were able to better estimate the hemicelluloses content of biomass by the deconvolution of DTG curves. Their computations assumed a multi-component pyrolysis model in which hemicelluloses, cellulose and lignin have independent parallel reactions. However, as Cozzani et al. (1997) [39] pointed out, these macro-components are intricately linked and the occurrence of their interactions during thermal degradation cannot be entirely overruled.

Chemometric models constructed for the volatile matter content of biomass had the best predictive performance (R^2^–0.92; RPD– 3.58). The predictive power for both PLS and PCR models for fixed carbon were also good. Unfortunately, models constructed for the ash content prediction didn’t do as well. As TGs are a function of the mass loss of organics as samples are heated, the incombustibility of the inorganics might not have been adequately captured, as such, the poor performance of the ash models.

The performance of TG-based models developed in this study is similar to what have been reported in the literature for other widely utilized high throughput tools such as near infrared spectroscopy (NIR) [40,41] and Fourier transform infrared spectroscopy (FTIR) [42,43]. Lande et al. (2010) [44] constructed TGA-based PLS models to predict the furfuryl alcohol polymer content of *Pinus sylvestris* treated in a commercial plant. Depending on the pretreatment method used, as much as 94% of the variance could be accounted for. Comparing to NIR-based models, the authors concluded that both tools had similar predictive powers; although more LVs were required in TGA models to attain this parity.

**Fig 3:**
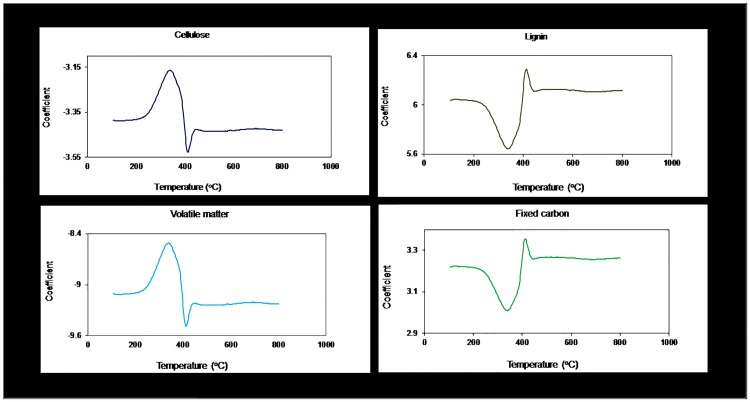
A plot of coefficients showing temperatures that had significant contribution in the prediction of thermochemical properties.

Regression coefficients of PCs (i.e. obtained from PCR) were investigated to identify temperatures that were important in modeling the various thermochemical constituents. PCs instead of LVs were employed for model interpretation because as mentioned earlier, the PCR algorithm only regards the X data when extracting its factors; as such most of the information in the original data is preserved. Also in an earlier study, we had determined that PC loadings worked better than LV loadings for model interpretation [45]. A plot of the regression coefficients is presented in Fig 3. Peaks were noted at about 320°C and 410°C for cellulose and lignin respectively. This is a suggestion that these temperatures had significant influence in the thermal degradation of the two chemical components. In kinetic studies of TGs/DTGs of lignocellulosic biomass, the thermal decomposition of cellulose have been determined to occur from 250°C to 440°C, while lignin degrades over a wider range (180°C to 900°C). Yang et al. (2006) [31] reported that the maximum mass loss was obtained at 355°C during the pyrolysis of pure cellulose. The authors could however not pinpoint the exact temperature at which the mass loss rate of lignin was highest. Instead, they noted that up until 700°C, the rate of lignin degradation was slow (< 0.5 wt%/°C) and this rate doubled at temperatures above 750°C.

The loadings plot of volatile matter was identical to that of cellulose and the plots of fixed carbon and lignin also looked similar. This suggests common latent variables are shared between these chemical and thermal reactivity properties. These findings buttress what has been reported in the literature that the thermally less stable polysaccharides are responsible for the volatile matter while the lignin with its higher percentage of elementary carbon and low oxygen contributes to the yield of fixed carbon [31,46,47]. A simple regression of cellulose versus volatile matter and lignin versus fixed carbon provided strong linear correlations between these properties (cellulose vs. volatile matter: R^2^ = 0.7; p < 0.05; lignin vs. fixed carbon: R^2^ = 0.77; p < 0.05), reinforcing the literature.

### Comparing chemometric and kinetic models

Evaluation results of the performance of chemometric models compared to the kinetic models developed with temperature regimes adopted and modified from deconvolution studies reported in the literature are presented in Table 4. Although the calibration errors associated with the two modeling approaches were similar, the cross validation errors were quiet high for the kinetic models; an indication that these models will poorly predict the understudied properties of future unknowns. Between the kinetic models for chemistry, cellulose and lignin contents were better estimated with KIN-2 (i.e. cellulose as the mass loss occurring between 360°C to 440°C and lignin as the mass after 440°C) than with KIN-1 (cellulose: 350°C to 500°C; lignin: after 500°C). As can be seen from Table 4, the chemometric models outperformed the kinetic models in all instances.

**Table 4:** Predictive performance of TG-based chemometric models versus kinetic models.

		SEC	SECV	RPD	R^2^
Lignin	PCR	1.63	1.76	2.37	0.82
	KIN-1	1.72	6.56	0.63	0.83
	KIN-2	1.72	2.65	1.57	0.81
Cellulose	PCR	3.45	4.02	1.85	0.7
	KIN-1	3.28	16.56	0.45	0.76
	KIN-2	3.22	5.95	1.25	0.71
Volatile matter	PCR	0.78	0.79	3.58	0.92
	KIN-3	0.74	1.45	1.57	0.90
Fixed carbon	PCR	1.22	1.32	2.39	0.82
	KIN-3	1.07	3.42	0.59	0.73
Ash	PCR	0.51	0.82	1.28	0.37
	KIN-3	0.40	0.91	0.58	0.46

In addition to the superior predictive capability of TG-based chemometric models over the conventional deconvolution of DTG curves for quantitative analysis of biomass, chemometric modeling has several other advantages.

Usually when TGA has been used in proximate analysis, heating regimes include temperature ramps and isothermal conditions in both inert and reactive gaseous atmospheres [27,32]. On the other hand, when TGA is used in kinetic studies for chemical composition estimation, samples are typically heated at a constant rate in an inert environment [17,31]. As such, in order to estimate the chemical and proximate composition of biomass, two separate experiments have conventionally been conducted. Using thermograms acquired under the inert conditions (total run time of 38 minutes), this study demonstrated that the chemical and thermal reactivity properties can be determined simultaneously when chemometrics is employed. In a recent study, Saldarriaga et al. (2015) [18] were also able to determine several properties of lignocellulosic biomass from a single TG/DTG employing deconvolution and empirical modeling. The authors reported about a TGA procedure that occurred in both inert and oxidative environments. However, their methodology took over 240 minutes of run time per sample. Thus, the 38 minute procedure developed in this current study presents a huge improvement in time saving.

**Fig 4:**
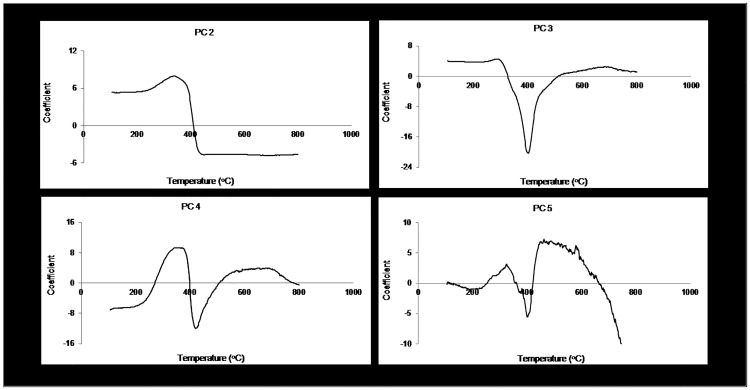
Loadings of PCs showing temperatures that had significant contribution in the prediction of chemical composition.

Furthermore, the application of chemometrics to TG data enabled the quantitative modeling of some monomeric sugars. This has not been possible with the deconvolution of DTGs. The fit statistics of models for predicting hemicellulose and its associated monomeric sugars is presented in Table 5. The predictive performance of the glucose model was good, with R^2^ of 0.77 and RPD of 2.11; thus meeting the criteria for preliminary screening. This was however not the case for mannose and galactose. Apart from these two hexoses having similar chemical structures to that of glucose (i.e. epimers), these also combine to form galactoglucomannans, the major hemicelluloses of softwoods. As such, analytical tools such as TGA might have some difficulty in distinguishing / predicting them. Nevertheless, by employing chemometrics in this study, elucidation of the PC loadings gave some insight into their thermal degradation. A plot of the PC loadings is presented in Fig 4. PC 2 and PC 4 were essential in estimating the three sugars. The position of the peak in PC 2 suggests that significant devolatilization of these hexoses occurred at 338°C. For mannose, PC 4 accounted for the most variation. PC 4 showed a more rounded peak ranging from 334 to 372°C; with a maxima at 358°C. This temperature shift could be due to the fact that mannose is the major component of the more stable backbone of galactoglucomannans (i.e. 0.1–1:1: 3–4) [48].

**Table 5:** Chemometric model statistics for monomeric sugars, hemicelluloses and holocellulose.

	PCs	SEC	SECV	RPD	R^2^	R^2^_Adj_
Glucose	3	3.17	3.51	2.14	0.78	0.77
Galactose	2	0.69	1.55	1.12	0.19	0.17
Mannose	2	0.63	1.31	1.15	0.22	0.20
Xylose	2	0.66	0.97	1.34	0.43	0.42
Arabinose	3	0.33	0.38	1.92	0.72	0.72
Hemicelluloses	3	0.98	2.81	1.08	0.11	0.09
Holocellulose	2	3.43	4.09	1.76	0.67	0.66

TGA performed better at predicting the five-ringed sugars of xylose and arabinose. The errors associated with the arabinose model were lower probably because this sugar is less similar to the abundant glucose, compared to xylose. PCs 2 and 4 once again made significant contributions in the modeling of this pentose. Xylose, which has been used in TGA kinetic studies to represent hemicelluloses was predicted with lesser success (R2–0.43; RPD– 1.3). Unlike for the other monomeric sugars, PC 3 and PC 5 subsequently explained most of the variation in the thermal degradation of xylose, Fig 4. Decomposition of xylose started at a much lower temperature, with maximum mass loss occurring at 288°C, Fig 4. Also, when Biagini et al. (2006) [49] modeled commercial xylan as a model for hemicelluloses, they reported an onset degradation temperature of 253°C, with bulk mass loss occurring at 299°C. However, Werner et al. (2014) [50] found out in their study that xylan decomposed in two stages, with significant mass loss at 243°C to 292°C. Results from the monomeric sugars chemometric models suggest that, thermal decomposition of hemicelluloses range from 282°C to 372°C. These are consistent with what have been reported in the literature using the deconvolution of thermograms [14,17,30,31].

The poor prediction of the composite hemicelluloses could be a consequent of the difficulty in modeling mannose and galactose. However, TG-based chemometric models adequately predicted the holocellulose content of biomass. The holocellulose content could thus be used together with predicted cellulose content to provide a rough estimation of the hemicelluloses.

It should however be noted that, the models developed in this study are limited to the biomass types (i.e. wood, bark and foliage) of southern yellow pines and will not work if applied to different biomass types. A bioprocessing facility utilizing TGA coupled with chemometrics as a tool to estimate the composition of biomass will need to calibrate their system with sample material that is representative of their feedstock.

## Conclusions

This study has demonstrated that chemometric modeling of thermogravimetric (TG) data can be used as an alternative approach to rapidly estimate the chemical and proximate composition of lignocellulosic biomass. Developed chemometric models had superior predictive capabilities than models constructed using the conventional deconvolution of TGs. PLS and PCR models calibrated with normalized TG data performed very well in predicting especially the lignin (R^2^–0.82; RPD– 2.37) and volatile matter (R^2^–0.92; RPD– 3.58) contents of forest-derived biomass. Examination of the loadings plots of PCR models suggested that significant degradation of cellulose and lignin occurred at around 320°C and 410°C respectively. Furthermore, these plots showed that common latent variables were shared between cellulose and volatile matter content; and between lignin and fixed carbon content.

The methodology developed in this study involved a 38-minutes procedure that allowed the simultaneous estimation of the chemical and proximate composition of lignocellulosic biomass from the same TG data. In addition to its rapidity and simplicity, this approach enabled the prediction of some monomeric sugars.

Elucidation of PC loadings showed similar plots for volatile matter and cellulose, and for fixed carbon and lignin; an indication that common latent variables are shared between these chemical and thermal reactivity properties. Results from this study buttresses literature that have reported that the less thermally stable polysaccharides are responsible for the yield of volatiles whereas the more recalcitrant lignin with its higher percentage of elementary carbon contributes to the yield of fixed carbon. In addition, the findings suggested that, the thermal degradation of xylose started at a much lower temperature, with significant mass loss occurring at 288°C, compared to the other monomeric sugars in lignocellulosic biomass. According to the literature, these have not been attainable by the conventional deconvolution of DTGs obtained from the composite lignocellulosic biomass. Future studies will be necessary to further investigate the capability of chemometrics to model the thermal degradation and quantitative prediction of the individual monomeric sugars.

## Supporting information

S1 File TG dataset used for model development. (PDF)
