# Supplementary material for: Chemometric modeling of thermogravimetric data for the compositional analysis of forest biomass
Source: PLoS One. 2017 Mar 2;12(3):e0172999. doi: 10.1371/journal.pone.0172999 (PMC5333859; doi:10.1371/journal.pone.0172999)
Supplement: S1 File — (PDF) [file pone.0172999.s001.pdf]

	106	108	110	112	114	116	118	120
PWT1	5.879	5.881	5.882	5.883	5.884	5.884	5.884	5.884
PWT2	6.33	6.332	6.332	6.333	6.333	6.333	6.334	6.333
PWT3	8.807	8.776	8.777	8.777	8.777	8.778	8.778	8.778
PWT4	9.373	9.33	9.33	9.331	9.331	9.333	9.331	9.333
PWT5	6.485	6.484	6.487	6.486	6.486	6.486	6.486	6.486
PWT6	8.132	8.134	8.134	8.134	8.135	8.135	8.135	8.134
PWT7	7.182	7.183	7.183	7.183	7.183	7.183	7.183	7.183
PWT8	8.03	8.031	8.033	8.032	8.033	8.033	8.033	8.033
PWT9	7.472	7.474	7.474	7.474	7.475	7.474	7.475	7.475
PWT10	7.817	7.819	7.819	7.819	7.819	7.818	7.819	7.819
PWB1	7.001	6.983	6.984	6.983	6.983	6.984	6.985	6.984
PWB2	8.774	8.757	8.757	8.757	8.757	8.757	8.757	8.758
PWB3	6.699	6.679	6.679	6.681	6.681	6.681	6.68	6.681
PWB4	9.436	9.396	9.397	9.399	9.399	9.399	9.399	9.399
PWB5	6.972	6.958	6.959	6.959	6.959	6.959	6.959	6.959
PWB6	6.95	6.917	6.916	6.917	6.917	6.919	6.92	6.919
PWB7	7.985	7.947	7.948	7.948	7.951	7.95	7.951	7.95
PWB8	7.772	7.746	7.742	7.742	7.743	7.743	7.743	7.743
PWB9	7.088	7.076	7.078	7.078	7.078	7.078	7.078	7.076
PWB10	5.647	5.648	5.649	5.647	5.647	5.649	5.648	5.65
PSL1	7.077	7.079	7.08	7.079	7.08	7.08	7.079	7.079
PSL2	8.961	8.963	8.963	8.963	8.963	8.963	8.963	8.963
PSL3	5.684	5.685	5.686	5.686	5.686	5.687	5.687	5.687
PSL4	7.525	7.526	7.527	7.526	7.526	7.527	7.526	7.526
PSL5	7.779	7.78	7.781	7.78	7.78	7.779	7.78	7.78
PSL6	6.927	6.927	6.929	6.929	6.929	6.929	6.929	6.93
PSL7	8.434	8.433	8.434	8.434	8.434	8.436	8.435	8.435
PSL8	8.097	8.095	8.097	8.097	8.097	8.097	8.097	8.097
PSL9	5.968	5.968	5.968	5.969	5.968	5.968	5.967	5.967
PSL10	6.61	6.611	6.61	6.61	6.611	6.611	6.61	6.612
PCW1	5.658	5.66	5.66	5.66	5.66	5.66	5.66	5.66
PCW2	7.384	7.384	7.385	7.387	7.387	7.387	7.387	7.386
PCW3	8.186	8.188	8.189	8.189	8.189	8.189	8.189	8.189
PCW4	8.056	8.057	8.057	8.058	8.058	8.058	8.058	8.059
PCW5	7.575	7.575	7.576	7.577	7.578	7.578	7.578	7.578
PCW6	8.481	8.481	8.483	8.483	8.483	8.483	8.483	8.483
PCW7	7.467	7.468	7.468	7.468	7.467	7.467	7.468	7.467
PCW8	6.803	6.803	6.805	6.804	6.803	6.805	6.805	6.805
PCW9	8.202	8.202	8.202	8.202	8.202	8.202	8.203	8.203
PCW10	6.342	6.342	6.343	6.344	6.343	6.344	6.344	6.345

122	124	126	128	130	132	134	136	138
5.884	5.884	5.884	5.884	5.884	5.884	5.884	5.883	5.884
6.333	6.334	6.334	6.333	6.334	6.333	6.334	6.333	6.333
8.777	8.778	8.778	8.777	8.778	8.777	8.775	8.777	8.775
9.333	9.332	9.331	9.331	9.331	9.329	9.33	9.33	9.331
6.486	6.486	6.485	6.486	6.485	6.485	6.485	6.484	6.484
8.135	8.135	8.135	8.134	8.134	8.134	8.134	8.132	8.132
7.183	7.181	7.183	7.181	7.181	7.181	7.18	7.18	7.178
8.033	8.033	8.033	8.034	8.031	8.032	8.03	8.031	8.03
7.474	7.475	7.475	7.475	7.475	7.474	7.474	7.474	7.473
7.819	7.819	7.818	7.819	7.817	7.817	7.817	7.816	7.816
6.984	6.984	6.983	6.982	6.982	6.982	6.981	6.98	6.98
8.757	8.758	8.758	8.757	8.757	8.755	8.757	8.755	8.754
6.682	6.681	6.681	6.679	6.681	6.679	6.679	6.679	6.677
9.399	9.399	9.397	9.396	9.397	9.397	9.395	9.394	9.394
6.959	6.959	6.959	6.959	6.96	6.959	6.96	6.96	6.959
6.917	6.917	6.918	6.918	6.917	6.916	6.917	6.916	6.916
7.95	7.95	7.95	7.95	7.95	7.948	7.948	7.947	7.947
7.742	7.742	7.742	7.742	7.742	7.741	7.742	7.741	7.741
7.078	7.077	7.076	7.077	7.077	7.075	7.075	7.074	7.074
5.65	5.649	5.649	5.649	5.647	5.649	5.647	5.647	5.648
7.079	7.079	7.079	7.079	7.079	7.079	7.078	7.077	7.076
8.961	8.962	8.962	8.961	8.961	8.96	8.959	8.958	8.959
5.687	5.689	5.688	5.689	5.687	5.686	5.687	5.687	5.687
7.526	7.525	7.525	7.525	7.525	7.525	7.525	7.522	7.522
7.78	7.78	7.779	7.779	7.779	7.779	7.777	7.777	7.777
6.93	6.929	6.928	6.929	6.927	6.928	6.927	6.926	6.927
8.434	8.434	8.435	8.434	8.434	8.434	8.433	8.433	8.432
8.098	8.097	8.097	8.097	8.095	8.094	8.095	8.095	8.092
5.968	5.967	5.967	5.967	5.967	5.967	5.967	5.967	5.967
6.611	6.61	6.61	6.61	6.61	6.609	6.609	6.608	6.608
5.659	5.66	5.658	5.658	5.657	5.657	5.657	5.657	5.657
7.387	7.387	7.386	7.385	7.385	7.385	7.385	7.384	7.384
8.189	8.189	8.189	8.188	8.188	8.188	8.188	8.187	8.188
8.059	8.058	8.058	8.058	8.059	8.058	8.057	8.057	8.057
7.577	7.578	7.578	7.576	7.578	7.578	7.577	7.577	7.576
8.483	8.483	8.482	8.483	8.483	8.483	8.481	8.481	8.481
7.468	7.468	7.468	7.467	7.467	7.467	7.467	7.466	7.467
6.804	6.805	6.804	6.805	6.805	6.805	6.804	6.803	6.804
8.202	8.203	8.205	8.203	8.203	8.203	8.203	8.202	8.202
6.344	6.346	6.344	6.345	6.345	6.344	6.344	6.344	6.343

140	142	144	146	148	150	152	154	156
5.883	5.883	5.883	5.883	5.883	5.883	5.883	5.883	5.883
6.333	6.333	6.332	6.332	6.331	6.332	6.33	6.33	6.331
8.775	8.775	8.774	8.774	8.772	8.772	8.772	8.771	8.772
9.33	9.328	9.328	9.327	9.327	9.327	9.327	9.324	9.324
6.485	6.483	6.483	6.482	6.481	6.481	6.48	6.48	6.479
8.132	8.131	8.132	8.132	8.132	8.131	8.13	8.129	8.128
7.177	7.176	7.176	7.176	7.176	7.176	7.175	7.173	7.174
8.028	8.028	8.029	8.028	8.027	8.027	8.026	8.026	8.025
7.471	7.472	7.471	7.471	7.469	7.469	7.469	7.469	7.468
7.816	7.814	7.816	7.815	7.814	7.813	7.813	7.813	7.813
6.979	6.978	6.977	6.977	6.977	6.976	6.974	6.974	6.973
8.754	8.753	8.751	8.75	8.751	8.75	8.75	8.75	8.748
6.677	6.678	6.677	6.677	6.676	6.675	6.675	6.675	6.675
9.394	9.393	9.392	9.392	9.39	9.39	9.392	9.39	9.389
6.959	6.959	6.958	6.959	6.959	6.959	6.957	6.957	6.954
6.914	6.914	6.913	6.913	6.912	6.912	6.911	6.91	6.908
7.947	7.945	7.944	7.944	7.944	7.943	7.943	7.941	7.941
7.741	7.739	7.739	7.739	7.738	7.738	7.738	7.736	7.736
7.073	7.073	7.073	7.073	7.071	7.071	7.07	7.071	7.07
5.647	5.648	5.647	5.648	5.646	5.647	5.646	5.646	5.645
7.076	7.076	7.075	7.073	7.074	7.072	7.072	7.071	7.07
8.956	8.956	8.955	8.955	8.955	8.953	8.952	8.951	8.951
5.686	5.686	5.686	5.686	5.686	5.684	5.685	5.685	5.684
7.522	7.521	7.521	7.519	7.519	7.517	7.518	7.517	7.516
7.777	7.776	7.775	7.774	7.773	7.774	7.773	7.771	7.771
6.926	6.926	6.925	6.925	6.925	6.925	6.925	6.923	6.923
8.432	8.432	8.43	8.431	8.43	8.43	8.429	8.429	8.428
8.092	8.092	8.092	8.091	8.091	8.09	8.089	8.088	8.088
5.967	5.965	5.965	5.965	5.963	5.962	5.962	5.962	5.959
6.607	6.608	6.608	6.606	6.605	6.606	6.606	6.607	6.605
5.656	5.657	5.656	5.656	5.657	5.657	5.656	5.657	5.656
7.384	7.384	7.384	7.384	7.384	7.385	7.384	7.384	7.382
8.187	8.186	8.185	8.186	8.185	8.185	8.185	8.185	8.185
8.057	8.055	8.055	8.054	8.053	8.054	8.053	8.052	8.052
7.575	7.575	7.575	7.575	7.575	7.575	7.574	7.573	7.573
8.48	8.48	8.481	8.48	8.479	8.479	8.477	8.477	8.477
7.465	7.465	7.465	7.465	7.465	7.464	7.465	7.465	7.464
6.803	6.803	6.803	6.803	6.804	6.803	6.803	6.803	6.803
8.202	8.202	8.201	8.201	8.201	8.2	8.201	8.2	8.199
6.344	6.344	6.343	6.344	6.343	6.343	6.344	6.343	6.344

158	160	162	164	166	168	170	172	174
5.883	5.883	5.883	5.883	5.881	5.881	5.881	5.881	5.88
6.33	6.33	6.33	6.33	6.332	6.33	6.33	6.33	6.329
8.771	8.771	8.771	8.771	8.77	8.77	8.77	8.77	8.768
9.324	9.323	9.324	9.323	9.321	9.32	9.32	9.32	9.318
6.479	6.478	6.477	6.477	6.476	6.475	6.476	6.476	6.475
8.126	8.126	8.126	8.126	8.124	8.124	8.124	8.123	8.122
7.173	7.173	7.173	7.171	7.17	7.171	7.171	7.17	7.17
8.025	8.025	8.024	8.025	8.024	8.023	8.023	8.023	8.021
7.468	7.466	7.467	7.467	7.468	7.467	7.465	7.466	7.465
7.813	7.812	7.813	7.814	7.813	7.813	7.813	7.811	7.811
6.974	6.973	6.972	6.972	6.97	6.97	6.97	6.969	6.969
8.748	8.747	8.746	8.745	8.746	8.747	8.745	8.745	8.744
6.677	6.675	6.674	6.674	6.673	6.674	6.673	6.672	6.671
9.387	9.387	9.387	9.386	9.385	9.384	9.384	9.384	9.383
6.956	6.954	6.953	6.954	6.953	6.953	6.953	6.953	6.952
6.907	6.907	6.906	6.905	6.906	6.904	6.904	6.903	6.903
7.94	7.941	7.941	7.938	7.94	7.938	7.938	7.939	7.937
7.736	7.736	7.735	7.733	7.735	7.732	7.732	7.731	7.731
7.069	7.068	7.067	7.067	7.067	7.066	7.066	7.064	7.064
5.646	5.646	5.645	5.644	5.643	5.643	5.642	5.643	5.642
7.07	7.07	7.068	7.068	7.066	7.067	7.066	7.065	7.064
8.951	8.949	8.948	8.948	8.946	8.946	8.946	8.945	8.943
5.684	5.684	5.684	5.684	5.684	5.685	5.686	5.685	5.684
7.515	7.515	7.513	7.512	7.512	7.51	7.511	7.509	7.508
7.77	7.771	7.771	7.77	7.769	7.767	7.767	7.767	7.766
6.922	6.921	6.92	6.92	6.92	6.919	6.919	6.92	6.918
8.428	8.427	8.426	8.426	8.427	8.426	8.426	8.426	8.426
8.088	8.087	8.085	8.085	8.085	8.085	8.084	8.085	8.084
5.961	5.959	5.958	5.958	5.958	5.957	5.957	5.955	5.955
6.604	6.604	6.604	6.603	6.604	6.602	6.601	6.601	6.602
5.656	5.654	5.656	5.654	5.654	5.654	5.654	5.653	5.653
7.382	7.382	7.382	7.382	7.382	7.382	7.382	7.382	7.381
8.185	8.185	8.184	8.184	8.184	8.182	8.183	8.182	8.182
8.053	8.051	8.052	8.051	8.051	8.051	8.051	8.051	8.049
7.573	7.572	7.572	7.572	7.572	7.572	7.572	7.572	7.571
8.477	8.477	8.476	8.476	8.476	8.476	8.475	8.475	8.476
7.464	7.463	7.465	7.464	7.464	7.463	7.464	7.462	7.463
6.803	6.803	6.802	6.803	6.802	6.802	6.801	6.802	6.801
8.199	8.199	8.199	8.198	8.198	8.197	8.196	8.196	8.196
6.343	6.343	6.343	6.344	6.343	6.343	6.343	6.343	6.342

176	178	180	182	184	186	188	190	192
5.88	5.88	5.881	5.88	5.878	5.878	5.877	5.878	5.878
6.329	6.328	6.327	6.327	6.328	6.328	6.327	6.329	6.327
8.768	8.768	8.768	8.767	8.767	8.766	8.765	8.765	8.765
9.318	9.317	9.317	9.316	9.316	9.315	9.316	9.314	9.314
6.475	6.474	6.473	6.472	6.472	6.47	6.47	6.469	6.469
8.122	8.121	8.121	8.121	8.121	8.119	8.118	8.117	8.117
7.169	7.168	7.168	7.168	7.168	7.167	7.166	7.166	7.166
8.023	8.021	8.02	8.018	8.018	8.017	8.017	8.015	8.017
7.465	7.465	7.464	7.464	7.462	7.462	7.463	7.461	7.46
7.811	7.811	7.81	7.811	7.81	7.811	7.81	7.81	7.809
6.968	6.969	6.967	6.967	6.967	6.967	6.966	6.966	6.966
8.744	8.744	8.744	8.744	8.745	8.744	8.745	8.745	8.744
6.672	6.671	6.669	6.669	6.67	6.668	6.668	6.668	6.667
9.383	9.382	9.382	9.382	9.382	9.381	9.38	9.381	9.38
6.952	6.952	6.95	6.951	6.95	6.949	6.949	6.949	6.947
6.903	6.903	6.903	6.902	6.9	6.902	6.902	6.9	6.902
7.937	7.937	7.938	7.937	7.938	7.938	7.937	7.937	7.938
7.731	7.731	7.731	7.729	7.728	7.728	7.728	7.728	7.727
7.064	7.064	7.064	7.063	7.063	7.062	7.061	7.06	7.06
5.641	5.642	5.64	5.639	5.64	5.64	5.639	5.639	5.638
7.062	7.063	7.062	7.062	7.06	7.06	7.058	7.058	7.056
8.942	8.942	8.941	8.94	8.939	8.936	8.936	8.935	8.935
5.683	5.684	5.684	5.683	5.683	5.683	5.682	5.681	5.681
7.507	7.505	7.505	7.505	7.503	7.502	7.502	7.501	7.501
7.767	7.766	7.765	7.766	7.764	7.764	7.764	7.765	7.763
6.918	6.918	6.917	6.916	6.916	6.915	6.915	6.913	6.912
8.424	8.423	8.424	8.424	8.423	8.423	8.422	8.422	8.422
8.085	8.084	8.084	8.082	8.082	8.081	8.081	8.081	8.08
5.955	5.954	5.954	5.954	5.954	5.952	5.951	5.952	5.951
6.602	6.602	6.602	6.603	6.603	6.602	6.602	6.601	6.602
5.653	5.653	5.651	5.65	5.651	5.65	5.65	5.65	5.648
7.381	7.381	7.381	7.381	7.381	7.381	7.381	7.379	7.379
8.182	8.181	8.182	8.182	8.182	8.181	8.181	8.181	8.181
8.049	8.051	8.049	8.051	8.051	8.051	8.052	8.051	8.051
7.57	7.57	7.572	7.573	7.573	7.573	7.573	7.573	7.573
8.474	8.474	8.473	8.473	8.473	8.473	8.473	8.473	8.474
7.462	7.462	7.461	7.462	7.462	7.461	7.461	7.461	7.461
6.802	6.802	6.801	6.801	6.801	6.801	6.801	6.801	6.801
8.196	8.196	8.196	8.196	8.196	8.195	8.195	8.195	8.195
6.343	6.342	6.343	6.342	6.342	6.343	6.342	6.34	6.34

194	196	198	200	202	204	206	208	210
5.877	5.877	5.877	5.876	5.876	5.876	5.876	5.876	5.875
6.327	6.327	6.326	6.326	6.326	6.326	6.326	6.324	6.324
8.764	8.762	8.761	8.761	8.758	8.758	8.758	8.755	8.754
9.312	9.311	9.31	9.31	9.309	9.307	9.307	9.307	9.306
6.467	6.467	6.466	6.466	6.466	6.466	6.463	6.465	6.463
8.117	8.116	8.116	8.115	8.114	8.112	8.111	8.111	8.109
7.166	7.165	7.164	7.163	7.163	7.162	7.161	7.161	7.16
8.015	8.015	8.014	8.014	8.013	8.014	8.013	8.014	8.014
7.459	7.459	7.458	7.457	7.456	7.454	7.454	7.454	7.454
7.809	7.808	7.807	7.807	7.809	7.806	7.807	7.806	7.806
6.965	6.964	6.964	6.963	6.963	6.963	6.963	6.962	6.962
8.744	8.744	8.743	8.744	8.744	8.744	8.744	8.744	8.744
6.667	6.666	6.667	6.665	6.666	6.667	6.667	6.668	6.667
9.38	9.379	9.379	9.38	9.379	9.378	9.379	9.379	9.377
6.948	6.947	6.947	6.947	6.946	6.946	6.946	6.946	6.946
6.9	6.901	6.9	6.9	6.902	6.902	6.902	6.902	6.9
7.937	7.937	7.937	7.937	7.936	7.937	7.934	7.935	7.935
7.726	7.726	7.725	7.726	7.726	7.726	7.725	7.725	7.725
7.061	7.061	7.06	7.059	7.058	7.058	7.059	7.061	7.059
5.639	5.639	5.639	5.639	5.639	5.638	5.639	5.639	5.639
7.055	7.053	7.05	7.05	7.049	7.046	7.045	7.043	7.043
8.933	8.933	8.932	8.93	8.929	8.928	8.925	8.923	8.923
5.68	5.68	5.68	5.68	5.679	5.679	5.677	5.676	5.676
7.501	7.499	7.498	7.497	7.496	7.495	7.494	7.493	7.492
7.763	7.764	7.763	7.763	7.763	7.763	7.762	7.762	7.76
6.911	6.912	6.909	6.909	6.907	6.906	6.906	6.905	6.903
8.419	8.419	8.419	8.418	8.417	8.417	8.417	8.414	8.414
8.078	8.078	8.077	8.077	8.076	8.075	8.075	8.073	8.073
5.951	5.95	5.951	5.949	5.949	5.948	5.947	5.946	5.944
6.601	6.601	6.601	6.601	6.599	6.598	6.598	6.597	6.597
5.649	5.649	5.649	5.648	5.648	5.648	5.649	5.648	5.647
7.379	7.379	7.38	7.379	7.379	7.38	7.379	7.378	7.377
8.181	8.179	8.181	8.18	8.181	8.179	8.179	8.181	8.18
8.051	8.051	8.051	8.049	8.051	8.049	8.049	8.05	8.049
7.573	7.573	7.573	7.575	7.573	7.575	7.573	7.575	7.573
8.474	8.473	8.473	8.474	8.473	8.474	8.473	8.474	8.473
7.46	7.461	7.461	7.46	7.459	7.458	7.459	7.458	7.457
6.801	6.801	6.799	6.799	6.798	6.799	6.799	6.8	6.799
8.195	8.195	8.195	8.193	8.194	8.192	8.192	8.191	8.192
6.341	6.34	6.341	6.34	6.339	6.339	6.339	6.337	6.338

212	214	216	218	220	222	224	226	228
5.874	5.873	5.873	5.871	5.87	5.87	5.868	5.867	5.866
6.324	6.324	6.323	6.323	6.323	6.321	6.322	6.32	6.32
8.754	8.751	8.751	8.75	8.749	8.748	8.745	8.744	8.742
9.306	9.303	9.301	9.3	9.298	9.297	9.296	9.294	9.291
6.462	6.462	6.463	6.462	6.462	6.46	6.46	6.459	6.457
8.109	8.108	8.107	8.105	8.105	8.103	8.101	8.101	8.099
7.158	7.158	7.157	7.156	7.154	7.153	7.153	7.15	7.148
8.013	8.012	8.011	8.012	8.011	8.01	8.008	8.008	8.007
7.452	7.452	7.451	7.45	7.449	7.448	7.448	7.446	7.446
7.806	7.805	7.804	7.803	7.803	7.801	7.8	7.8	7.799
6.96	6.959	6.959	6.957	6.956	6.956	6.953	6.953	6.952
8.743	8.743	8.744	8.742	8.742	8.742	8.742	8.741	8.741
6.667	6.666	6.667	6.667	6.666	6.667	6.665	6.665	6.664
9.378	9.378	9.376	9.376	9.375	9.374	9.374	9.373	9.372
6.944	6.944	6.943	6.942	6.942	6.942	6.94	6.939	6.937
6.9	6.9	6.9	6.899	6.899	6.899	6.897	6.897	6.897
7.934	7.935	7.934	7.934	7.934	7.933	7.934	7.933	7.932
7.724	7.723	7.723	7.723	7.723	7.722	7.721	7.721	7.72
7.058	7.058	7.057	7.057	7.057	7.056	7.056	7.055	7.054
5.64	5.64	5.64	5.64	5.639	5.639	5.639	5.637	5.638
7.042	7.04	7.039	7.036	7.035	7.033	7.032	7.03	7.028
8.921	8.919	8.917	8.915	8.913	8.911	8.909	8.906	8.902
5.675	5.674	5.674	5.674	5.673	5.671	5.67	5.669	5.669
7.491	7.489	7.486	7.487	7.485	7.483	7.481	7.478	7.477
7.759	7.76	7.759	7.757	7.757	7.755	7.754	7.753	7.75
6.902	6.9	6.899	6.899	6.896	6.895	6.893	6.89	6.889
8.412	8.412	8.41	8.409	8.409	8.407	8.406	8.403	8.402
8.071	8.069	8.068	8.067	8.064	8.062	8.061	8.059	8.058
5.944	5.942	5.942	5.94	5.938	5.937	5.934	5.933	5.931
6.595	6.595	6.595	6.594	6.592	6.592	6.592	6.59	6.588
5.647	5.648	5.648	5.648	5.647	5.648	5.647	5.647	5.647
7.377	7.377	7.376	7.376	7.376	7.375	7.375	7.374	7.375
8.179	8.178	8.178	8.178	8.178	8.176	8.176	8.175	8.175
8.051	8.049	8.048	8.048	8.048	8.048	8.048	8.048	8.047
7.573	7.572	7.573	7.572	7.573	7.573	7.573	7.572	7.571
8.473	8.473	8.473	8.473	8.473	8.473	8.472	8.472	8.471
7.458	7.458	7.457	7.457	7.457	7.455	7.455	7.454	7.454
6.798	6.798	6.796	6.796	6.796	6.795	6.796	6.794	6.793
8.192	8.191	8.189	8.189	8.189	8.189	8.188	8.188	8.185
6.337	6.337	6.336	6.336	6.336	6.336	6.334	6.334	6.333

230	232	234	236	238	240	242	244	246
5.866	5.864	5.863	5.861	5.861	5.86	5.858	5.858	5.854
6.319	6.317	6.317	6.316	6.314	6.313	6.312	6.31	6.309
8.74	8.74	8.738	8.735	8.734	8.731	8.73	8.727	8.725
9.29	9.288	9.286	9.284	9.281	9.279	9.274	9.273	9.27
6.456	6.455	6.455	6.453	6.452	6.45	6.449	6.448	6.446
8.098	8.097	8.095	8.093	8.092	8.088	8.088	8.084	8.082
7.147	7.146	7.143	7.141	7.14	7.137	7.134	7.131	7.13
8.005	8.005	8.004	8.003	8.001	8	7.998	7.997	7.995
7.446	7.445	7.444	7.444	7.442	7.441	7.439	7.436	7.435
7.797	7.794	7.794	7.793	7.791	7.789	7.788	7.786	7.784
6.95	6.948	6.946	6.946	6.945	6.943	6.943	6.94	6.94
8.741	8.74	8.739	8.738	8.737	8.737	8.735	8.734	8.733
6.664	6.664	6.663	6.663	6.662	6.661	6.659	6.658	6.658
9.373	9.37	9.37	9.369	9.368	9.367	9.366	9.364	9.363
6.936	6.936	6.932	6.93	6.927	6.926	6.924	6.92	6.918
6.897	6.896	6.896	6.896	6.894	6.894	6.893	6.892	6.89
7.931	7.93	7.928	7.928	7.926	7.926	7.925	7.923	7.921
7.718	7.718	7.718	7.716	7.715	7.713	7.711	7.709	7.708
7.054	7.053	7.053	7.051	7.051	7.049	7.048	7.047	7.046
5.637	5.637	5.637	5.637	5.637	5.636	5.634	5.634	5.633
7.026	7.023	7.02	7.017	7.015	7.01	7.007	7.003	6.999
8.899	8.896	8.892	8.889	8.886	8.882	8.878	8.875	8.871
5.666	5.666	5.663	5.662	5.66	5.66	5.659	5.657	5.657
7.474	7.471	7.468	7.465	7.461	7.456	7.452	7.448	7.442
7.749	7.749	7.746	7.746	7.743	7.74	7.739	7.737	7.733
6.888	6.885	6.883	6.88	6.876	6.873	6.869	6.866	6.862
8.399	8.397	8.395	8.393	8.389	8.386	8.383	8.38	8.377
8.057	8.053	8.051	8.048	8.044	8.04	8.036	8.032	8.028
5.929	5.926	5.924	5.921	5.918	5.915	5.911	5.907	5.902
6.588	6.585	6.585	6.584	6.58	6.578	6.576	6.574	6.571
5.646	5.646	5.645	5.645	5.643	5.643	5.641	5.64	5.638
7.374	7.373	7.372	7.372	7.371	7.371	7.368	7.367	7.366
8.174	8.172	8.171	8.17	8.169	8.168	8.166	8.164	8.164
8.045	8.044	8.043	8.042	8.041	8.04	8.038	8.037	8.035
7.57	7.57	7.57	7.569	7.568	7.566	7.566	7.566	7.563
8.47	8.47	8.469	8.467	8.466	8.466	8.466	8.463	8.463
7.453	7.452	7.452	7.45	7.451	7.449	7.447	7.447	7.445
6.793	6.792	6.792	6.792	6.791	6.789	6.789	6.787	6.786
8.185	8.184	8.184	8.182	8.18	8.179	8.179	8.177	8.176
6.333	6.333	6.332	6.332	6.332	6.33	6.329	6.329	6.326

248	250	252	254	256	258	260	262	264
5.853	5.853	5.851	5.85	5.849	5.848	5.846	5.844	5.841
6.307	6.304	6.304	6.302	6.3	6.298	6.296	6.293	6.29
8.721	8.718	8.715	8.711	8.708	8.704	8.7	8.694	8.69
9.266	9.264	9.26	9.256	9.251	9.246	9.241	9.236	9.229
6.443	6.442	6.439	6.437	6.433	6.432	6.429	6.426	6.421
8.079	8.076	8.074	8.069	8.066	8.062	8.059	8.054	8.051
7.126	7.123	7.119	7.115	7.113	7.107	7.103	7.098	7.093
7.993	7.99	7.988	7.986	7.984	7.981	7.978	7.975	7.971
7.433	7.432	7.429	7.428	7.425	7.423	7.42	7.418	7.414
7.781	7.779	7.777	7.773	7.77	7.767	7.763	7.759	7.756
6.937	6.936	6.935	6.933	6.933	6.93	6.928	6.926	6.923
8.731	8.731	8.729	8.727	8.725	8.724	8.721	8.718	8.717
6.657	6.656	6.654	6.652	6.651	6.648	6.647	6.644	6.641
9.36	9.359	9.356	9.353	9.35	9.348	9.345	9.342	9.337
6.914	6.913	6.908	6.904	6.9	6.896	6.892	6.886	6.882
6.889	6.889	6.887	6.886	6.884	6.882	6.882	6.879	6.876
7.919	7.918	7.915	7.911	7.908	7.906	7.904	7.901	7.899
7.706	7.704	7.701	7.698	7.695	7.694	7.689	7.686	7.682
7.044	7.041	7.041	7.038	7.037	7.033	7.031	7.028	7.024
5.633	5.633	5.631	5.629	5.629	5.627	5.626	5.623	5.622
6.993	6.99	6.985	6.979	6.974	6.968	6.963	6.955	6.948
8.866	8.861	8.856	8.852	8.848	8.842	8.836	8.829	8.822
5.659	5.659	5.66	5.661	5.664	5.665	5.667	5.669	5.67
7.438	7.432	7.425	7.419	7.411	7.404	7.395	7.387	7.378
7.73	7.727	7.724	7.722	7.717	7.713	7.709	7.704	7.699
6.858	6.853	6.849	6.843	6.836	6.832	6.825	6.819	6.812
8.372	8.367	8.363	8.357	8.353	8.346	8.34	8.333	8.326
8.024	8.018	8.012	8.007	8	7.995	7.987	7.979	7.971
5.898	5.892	5.888	5.882	5.876	5.871	5.864	5.855	5.847
6.569	6.566	6.562	6.559	6.555	6.551	6.547	6.541	6.538
5.637	5.638	5.636	5.634	5.633	5.63	5.628	5.626	5.624
7.364	7.362	7.361	7.361	7.358	7.357	7.354	7.352	7.349
8.162	8.159	8.158	8.155	8.152	8.149	8.146	8.144	8.139
8.031	8.031	8.03	8.028	8.026	8.024	8.021	8.018	8.014
7.562	7.56	7.558	7.556	7.556	7.553	7.552	7.551	7.549
8.461	8.459	8.459	8.457	8.454	8.453	8.45	8.447	8.444
7.443	7.441	7.439	7.439	7.437	7.435	7.432	7.431	7.428
6.785	6.785	6.782	6.781	6.778	6.776	6.775	6.772	6.769
8.174	8.172	8.171	8.168	8.166	8.164	8.161	8.159	8.156
6.326	6.323	6.322	6.32	6.319	6.317	6.315	6.313	6.31

266	268	270	272	274	276	278	280	282
5.841	5.839	5.836	5.834	5.831	5.827	5.823	5.819	5.815
6.287	6.286	6.282	6.279	6.275	6.271	6.266	6.262	6.257
8.684	8.678	8.673	8.666	8.659	8.651	8.642	8.633	8.624
9.224	9.219	9.212	9.204	9.197	9.187	9.179	9.171	9.16
6.419	6.415	6.409	6.406	6.4	6.395	6.389	6.383	6.375
8.046	8.041	8.035	8.029	8.022	8.016	8.008	8.002	7.991
7.089	7.083	7.076	7.069	7.061	7.053	7.045	7.035	7.025
7.968	7.964	7.96	7.956	7.951	7.946	7.94	7.934	7.928
7.411	7.408	7.404	7.4	7.395	7.391	7.386	7.38	7.373
7.752	7.747	7.741	7.736	7.73	7.723	7.714	7.707	7.7
6.92	6.917	6.915	6.912	6.906	6.903	6.899	6.893	6.889
8.714	8.711	8.707	8.704	8.7	8.695	8.692	8.686	8.681
6.639	6.637	6.634	6.63	6.626	6.622	6.618	6.613	6.608
9.334	9.33	9.326	9.32	9.316	9.31	9.305	9.296	9.289
6.876	6.871	6.865	6.859	6.852	6.845	6.837	6.83	6.822
6.873	6.87	6.868	6.864	6.862	6.857	6.853	6.849	6.843
7.896	7.893	7.888	7.884	7.878	7.873	7.868	7.861	7.856
7.678	7.674	7.669	7.665	7.659	7.652	7.645	7.638	7.634
7.02	7.017	7.013	7.008	7.004	6.999	6.993	6.987	6.98
5.619	5.617	5.616	5.613	5.609	5.606	5.603	5.599	5.594
6.94	6.931	6.923	6.914	6.904	6.892	6.881	6.868	6.856
8.815	8.808	8.798	8.789	8.779	8.77	8.758	8.747	8.734
5.671	5.673	5.673	5.673	5.671	5.671	5.669	5.664	5.66
7.367	7.358	7.345	7.333	7.321	7.308	7.294	7.278	7.263
7.693	7.687	7.682	7.675	7.667	7.658	7.65	7.641	7.631
6.803	6.796	6.786	6.778	6.768	6.758	6.746	6.736	6.721
8.319	8.31	8.3	8.292	8.28	8.27	8.257	8.244	8.231
7.962	7.953	7.943	7.933	7.92	7.909	7.897	7.883	7.868
5.84	5.831	5.823	5.812	5.801	5.791	5.78	5.768	5.755
6.533	6.525	6.521	6.514	6.508	6.5	6.49	6.481	6.473
5.621	5.618	5.614	5.611	5.608	5.604	5.6	5.596	5.591
7.348	7.345	7.341	7.337	7.334	7.331	7.327	7.322	7.318
8.135	8.132	8.128	8.122	8.118	8.112	8.107	8.1	8.092
8.012	8.01	8.005	8	7.995	7.991	7.986	7.98	7.974
7.545	7.542	7.539	7.536	7.533	7.529	7.526	7.521	7.517
8.44	8.437	8.435	8.432	8.427	8.423	8.418	8.413	8.407
7.425	7.422	7.42	7.417	7.414	7.408	7.403	7.4	7.395
6.769	6.766	6.762	6.759	6.756	6.752	6.748	6.744	6.739
8.154	8.149	8.146	8.144	8.138	8.134	8.129	8.124	8.119
6.308	6.306	6.303	6.3	6.297	6.293	6.289	6.286	6.28

284	286	288	290	292	294	296	298	300
5.809	5.804	5.799	5.791	5.784	5.778	5.769	5.76	5.75
6.252	6.246	6.24	6.233	6.227	6.22	6.213	6.203	6.195
8.613	8.602	8.591	8.578	8.564	8.55	8.533	8.519	8.5
9.15	9.138	9.127	9.116	9.103	9.088	9.074	9.058	9.041
6.368	6.361	6.352	6.343	6.333	6.323	6.312	6.301	6.289
7.984	7.973	7.964	7.953	7.942	7.931	7.918	7.904	7.89
7.014	7.004	6.992	6.979	6.965	6.951	6.938	6.922	6.906
7.922	7.914	7.907	7.898	7.89	7.88	7.869	7.859	7.848
7.367	7.359	7.351	7.342	7.332	7.322	7.311	7.3	7.287
7.691	7.681	7.67	7.659	7.646	7.635	7.621	7.608	7.594
6.883	6.877	6.872	6.865	6.857	6.848	6.839	6.83	6.819
8.675	8.668	8.663	8.654	8.646	8.638	8.627	8.616	8.606
6.603	6.598	6.591	6.584	6.578	6.57	6.561	6.553	6.543
9.282	9.273	9.265	9.255	9.246	9.233	9.222	9.209	9.196
6.815	6.809	6.8	6.791	6.782	6.773	6.763	6.753	6.743
6.838	6.832	6.826	6.82	6.811	6.803	6.794	6.784	6.775
7.848	7.841	7.831	7.823	7.814	7.804	7.793	7.782	7.77
7.625	7.615	7.605	7.595	7.584	7.573	7.561	7.548	7.535
6.973	6.965	6.957	6.948	6.939	6.929	6.919	6.907	6.896
5.592	5.586	5.582	5.575	5.569	5.564	5.556	5.55	5.542
6.842	6.828	6.812	6.797	6.78	6.763	6.745	6.726	6.707
8.722	8.708	8.692	8.677	8.659	8.642	8.624	8.604	8.583
5.653	5.646	5.634	5.625	5.612	5.6	5.586	5.57	5.554
7.247	7.229	7.211	7.191	7.172	7.152	7.131	7.107	7.082
7.62	7.608	7.596	7.58	7.566	7.55	7.533	7.516	7.497
6.708	6.695	6.681	6.665	6.648	6.632	6.615	6.596	6.576
8.216	8.202	8.184	8.168	8.15	8.131	8.112	8.092	8.07
7.854	7.838	7.822	7.804	7.786	7.769	7.749	7.73	7.707
5.743	5.729	5.715	5.7	5.684	5.669	5.652	5.634	5.617
6.463	6.453	6.443	6.43	6.418	6.403	6.39	6.375	6.36
5.586	5.581	5.576	5.57	5.563	5.555	5.547	5.539	5.53
7.311	7.307	7.301	7.295	7.287	7.281	7.272	7.263	7.252
8.084	8.076	8.067	8.058	8.048	8.037	8.025	8.012	7.999
7.968	7.961	7.953	7.944	7.935	7.927	7.915	7.905	7.893
7.512	7.506	7.502	7.495	7.488	7.48	7.473	7.463	7.455
8.4	8.395	8.387	8.38	8.372	8.363	8.354	8.343	8.333
7.39	7.384	7.379	7.371	7.364	7.355	7.348	7.34	7.33
6.734	6.728	6.722	6.715	6.709	6.701	6.692	6.684	6.674
8.114	8.108	8.101	8.094	8.086	8.078	8.069	8.059	8.049
6.276	6.27	6.266	6.259	6.253	6.245	6.237	6.228	6.219

302	304	306	308	310	312	314	316	318
5.74	5.729	5.716	5.704	5.691	5.677	5.661	5.645	5.628
6.186	6.175	6.163	6.152	6.14	6.126	6.113	6.099	6.082
8.483	8.463	8.443	8.423	8.402	8.379	8.355	8.33	8.305
9.025	9.007	8.988	8.967	8.948	8.925	8.901	8.876	8.849
6.277	6.264	6.25	6.235	6.221	6.204	6.187	6.17	6.151
7.874	7.859	7.842	7.825	7.808	7.788	7.767	7.747	7.723
6.889	6.87	6.851	6.829	6.807	6.784	6.759	6.733	6.704
7.835	7.821	7.807	7.793	7.777	7.76	7.744	7.724	7.703
7.274	7.261	7.245	7.23	7.213	7.195	7.177	7.158	7.137
7.578	7.561	7.544	7.528	7.509	7.489	7.47	7.449	7.427
6.809	6.798	6.785	6.773	6.758	6.744	6.73	6.713	6.696
8.593	8.581	8.566	8.552	8.538	8.52	8.502	8.485	8.465
6.533	6.522	6.511	6.498	6.487	6.473	6.459	6.444	6.428
9.182	9.166	9.151	9.134	9.117	9.099	9.081	9.06	9.038
6.731	6.719	6.707	6.691	6.677	6.661	6.643	6.626	6.606
6.764	6.753	6.74	6.729	6.714	6.701	6.686	6.67	6.652
7.758	7.744	7.729	7.714	7.7	7.682	7.665	7.646	7.626
7.52	7.506	7.49	7.473	7.455	7.437	7.418	7.399	7.376
6.882	6.869	6.856	6.84	6.823	6.807	6.789	6.77	6.751
5.534	5.526	5.516	5.508	5.497	5.487	5.475	5.463	5.451
6.686	6.666	6.643	6.62	6.597	6.572	6.547	6.52	6.493
8.56	8.537	8.513	8.489	8.462	8.435	8.406	8.376	8.344
5.537	5.52	5.503	5.485	5.466	5.446	5.426	5.403	5.383
7.058	7.032	7.005	6.976	6.949	6.918	6.887	6.855	6.823
7.477	7.456	7.436	7.414	7.39	7.364	7.338	7.311	7.283
6.557	6.535	6.515	6.492	6.47	6.446	6.421	6.394	6.368
8.047	8.024	7.999	7.974	7.947	7.919	7.892	7.86	7.828
7.684	7.661	7.637	7.612	7.586	7.559	7.53	7.501	7.469
5.598	5.578	5.557	5.536	5.514	5.491	5.467	5.442	5.417
6.343	6.327	6.307	6.29	6.27	6.249	6.227	6.206	6.182
5.522	5.51	5.502	5.491	5.48	5.467	5.453	5.441	5.427
7.243	7.231	7.218	7.205	7.191	7.177	7.161	7.144	7.127
7.983	7.969	7.953	7.936	7.92	7.901	7.88	7.862	7.84
7.88	7.867	7.854	7.839	7.823	7.806	7.789	7.769	7.75
7.446	7.435	7.424	7.413	7.402	7.388	7.374	7.36	7.343
8.322	8.31	8.297	8.281	8.268	8.253	8.236	8.22	8.201
7.32	7.31	7.297	7.287	7.274	7.26	7.244	7.23	7.214
6.665	6.652	6.639	6.628	6.615	6.601	6.585	6.569	6.553
8.038	8.025	8.014	8.001	7.987	7.971	7.957	7.939	7.924
6.21	6.2	6.187	6.176	6.162	6.148	6.134	6.12	6.104

320	322	324	326	328	330	332	334	336
5.61	5.592	5.572	5.553	5.531	5.509	5.485	5.46	5.435
6.065	6.047	6.029	6.009	5.987	5.964	5.94	5.915	5.89
8.276	8.247	8.217	8.184	8.15	8.115	8.078	8.041	8
8.82	8.79	8.756	8.722	8.685	8.646	8.604	8.562	8.514
6.131	6.111	6.088	6.067	6.043	6.017	5.99	5.962	5.933
7.698	7.67	7.644	7.613	7.583	7.549	7.514	7.477	7.438
6.674	6.643	6.609	6.577	6.539	6.501	6.459	6.418	6.374
7.682	7.659	7.635	7.608	7.581	7.552	7.521	7.487	7.452
7.115	7.093	7.068	7.043	7.015	6.986	6.956	6.925	6.892
7.403	7.379	7.352	7.324	7.295	7.265	7.234	7.201	7.166
6.678	6.659	6.64	6.616	6.592	6.568	6.542	6.516	6.486
8.444	8.422	8.397	8.372	8.346	8.318	8.289	8.257	8.223
6.412	6.394	6.374	6.355	6.332	6.309	6.285	6.259	6.232
9.016	8.991	8.964	8.937	8.908	8.875	8.844	8.81	8.775
6.584	6.561	6.537	6.511	6.483	6.454	6.424	6.39	6.354
6.634	6.615	6.594	6.572	6.548	6.526	6.499	6.473	6.444
7.605	7.583	7.557	7.531	7.503	7.473	7.442	7.408	7.371
7.355	7.331	7.307	7.282	7.255	7.226	7.197	7.169	7.137
6.729	6.707	6.683	6.658	6.631	6.603	6.576	6.544	6.511
5.436	5.42	5.405	5.388	5.369	5.349	5.329	5.307	5.283
6.465	6.435	6.403	6.372	6.338	6.306	6.272	6.235	6.201
8.311	8.276	8.24	8.204	8.166	8.127	8.085	8.042	8
5.36	5.336	5.309	5.285	5.257	5.231	5.202	5.174	5.145
6.789	6.754	6.717	6.681	6.642	6.603	6.562	6.52	6.477
7.254	7.222	7.189	7.156	7.12	7.084	7.045	7.007	6.967
6.339	6.31	6.28	6.249	6.215	6.182	6.147	6.112	6.075
7.795	7.76	7.724	7.687	7.647	7.607	7.566	7.524	7.481
7.439	7.405	7.369	7.333	7.295	7.256	7.214	7.173	7.13
5.391	5.364	5.336	5.305	5.277	5.246	5.215	5.183	5.15
6.159	6.134	6.107	6.081	6.052	6.023	5.993	5.961	5.929
5.412	5.395	5.378	5.36	5.341	5.323	5.301	5.28	5.258
7.109	7.088	7.068	7.048	7.026	7.004	6.978	6.953	6.928
7.818	7.794	7.767	7.74	7.712	7.684	7.654	7.62	7.586
7.729	7.708	7.684	7.66	7.634	7.607	7.578	7.55	7.52
7.327	7.308	7.29	7.269	7.246	7.223	7.199	7.174	7.147
8.181	8.162	8.14	8.118	8.094	8.068	8.042	8.013	7.984
7.198	7.18	7.162	7.141	7.121	7.098	7.075	7.051	7.025
6.534	6.515	6.495	6.475	6.452	6.429	6.406	6.381	6.355
7.906	7.885	7.865	7.843	7.819	7.794	7.769	7.741	7.713
6.086	6.069	6.05	6.029	6.009	5.987	5.965	5.942	5.918

338	340	342	344	346	348	350	352	354
5.408	5.379	5.354	5.324	5.294	5.262	5.228	5.195	5.16
5.863	5.835	5.804	5.776	5.744	5.712	5.679	5.643	5.607
7.959	7.917	7.871	7.826	7.779	7.73	7.68	7.627	7.571
8.466	8.414	8.36	8.306	8.247	8.189	8.127	8.063	7.997
5.901	5.869	5.835	5.801	5.766	5.728	5.689	5.65	5.611
7.396	7.352	7.304	7.257	7.206	7.154	7.1	7.045	6.988
6.328	6.282	6.233	6.183	6.13	6.077	6.022	5.966	5.908
7.415	7.376	7.335	7.295	7.249	7.204	7.155	7.105	7.052
6.857	6.822	6.784	6.747	6.705	6.664	6.62	6.575	6.527
7.131	7.093	7.054	7.015	6.971	6.929	6.884	6.839	6.788
6.457	6.424	6.393	6.358	6.322	6.285	6.247	6.206	6.166
8.188	8.151	8.113	8.072	8.03	7.985	7.939	7.889	7.839
6.203	6.174	6.143	6.11	6.076	6.043	6.006	5.968	5.929
8.738	8.698	8.658	8.616	8.572	8.525	8.477	8.427	8.374
6.315	6.276	6.234	6.188	6.143	6.095	6.043	5.99	5.935
6.415	6.383	6.352	6.317	6.282	6.246	6.207	6.169	6.127
7.334	7.296	7.254	7.214	7.169	7.124	7.077	7.029	6.977
7.104	7.071	7.037	6.998	6.96	6.922	6.881	6.84	6.795
6.477	6.44	6.402	6.364	6.325	6.282	6.24	6.195	6.149
5.258	5.233	5.205	5.175	5.146	5.114	5.082	5.048	5.014
6.162	6.125	6.086	6.047	6.006	5.964	5.921	5.88	5.835
7.955	7.91	7.862	7.812	7.763	7.711	7.66	7.606	7.55
5.113	5.082	5.05	5.016	4.981	4.947	4.91	4.874	4.837
6.434	6.39	6.345	6.299	6.254	6.208	6.159	6.11	6.062
6.928	6.884	6.842	6.798	6.752	6.704	6.656	6.607	6.556
6.038	6	5.963	5.925	5.885	5.845	5.803	5.76	5.716
7.436	7.39	7.345	7.295	7.246	7.194	7.144	7.09	7.033
7.088	7.044	6.997	6.951	6.903	6.854	6.803	6.753	6.7
5.115	5.082	5.045	5.009	4.972	4.936	4.897	4.857	4.816
5.896	5.861	5.827	5.79	5.752	5.714	5.674	5.635	5.592
5.234	5.208	5.183	5.156	5.128	5.098	5.067	5.036	5.004
6.9	6.87	6.839	6.807	6.773	6.74	6.705	6.67	6.629
7.551	7.515	7.477	7.437	7.395	7.354	7.307	7.259	7.21
7.489	7.455	7.42	7.384	7.347	7.309	7.268	7.227	7.182
7.12	7.091	7.061	7.027	6.994	6.96	6.923	6.886	6.844
7.953	7.919	7.885	7.848	7.81	7.771	7.729	7.686	7.638
6.999	6.97	6.941	6.91	6.878	6.844	6.809	6.771	6.733
6.328	6.3	6.27	6.239	6.208	6.173	6.139	6.102	6.064
7.683	7.652	7.62	7.586	7.55	7.513	7.474	7.435	7.391
5.893	5.866	5.838	5.81	5.781	5.749	5.717	5.682	5.648

356	358	360	362	364	366	368	370	372
5.125	5.085	5.045	5.004	4.961	4.918	4.873	4.827	4.779
5.569	5.531	5.488	5.445	5.401	5.355	5.307	5.258	5.206
7.511	7.45	7.388	7.322	7.255	7.186	7.114	7.039	6.962
7.93	7.857	7.786	7.713	7.636	7.56	7.482	7.404	7.323
5.567	5.525	5.48	5.434	5.386	5.337	5.288	5.236	5.184
6.928	6.865	6.802	6.737	6.669	6.604	6.537	6.466	6.395
5.85	5.786	5.723	5.658	5.594	5.528	5.461	5.392	5.325
6.997	6.941	6.883	6.822	6.76	6.696	6.628	6.561	6.492
6.48	6.429	6.377	6.322	6.266	6.21	6.149	6.088	6.026
6.737	6.685	6.631	6.576	6.519	6.46	6.399	6.336	6.271
6.122	6.078	6.031	5.982	5.932	5.879	5.828	5.774	5.718
7.784	7.729	7.672	7.61	7.548	7.484	7.416	7.348	7.277
5.888	5.847	5.804	5.757	5.712	5.663	5.614	5.562	5.509
8.321	8.262	8.203	8.14	8.075	8.01	7.94	7.868	7.793
5.879	5.821	5.76	5.698	5.637	5.574	5.509	5.444	5.38
6.083	6.038	5.992	5.943	5.894	5.838	5.784	5.729	5.671
6.923	6.868	6.811	6.752	6.692	6.631	6.57	6.504	6.436
6.75	6.703	6.653	6.602	6.549	6.494	6.436	6.376	6.313
6.099	6.05	5.998	5.945	5.889	5.833	5.775	5.715	5.653
4.979	4.941	4.902	4.862	4.819	4.776	4.733	4.687	4.639
5.79	5.742	5.694	5.644	5.594	5.544	5.491	5.439	5.384
7.493	7.433	7.374	7.312	7.25	7.186	7.12	7.051	6.983
4.797	4.758	4.717	4.675	4.632	4.589	4.544	4.499	4.452
6.01	5.956	5.905	5.851	5.796	5.74	5.682	5.624	5.566
6.504	6.45	6.394	6.337	6.279	6.219	6.161	6.099	6.036
5.67	5.625	5.577	5.527	5.478	5.427	5.376	5.323	5.27
6.976	6.917	6.858	6.796	6.734	6.67	6.605	6.535	6.467
6.646	6.588	6.531	6.471	6.411	6.35	6.287	6.224	6.16
4.775	4.734	4.689	4.646	4.601	4.555	4.509	4.462	4.414
5.55	5.505	5.46	5.412	5.364	5.314	5.263	5.21	5.156
4.969	4.931	4.892	4.852	4.809	4.766	4.72	4.673	4.623
6.589	6.546	6.501	6.454	6.405	6.354	6.3	6.245	6.184
7.156	7.101	7.043	6.984	6.924	6.862	6.795	6.728	6.657
7.138	7.091	7.042	6.988	6.934	6.877	6.816	6.753	6.688
6.803	6.758	6.71	6.662	6.61	6.558	6.5	6.442	6.381
7.592	7.541	7.488	7.432	7.372	7.311	7.248	7.181	7.111
6.691	6.648	6.601	6.553	6.502	6.448	6.392	6.333	6.273
6.024	5.984	5.939	5.894	5.845	5.796	5.744	5.691	5.634
7.345	7.298	7.247	7.195	7.139	7.083	7.024	6.963	6.897
5.611	5.573	5.533	5.491	5.445	5.399	5.35	5.299	5.248

374	376	378	380	382	384	386	388	390
4.728	4.674	4.623	4.567	4.514	4.456	4.399	4.342	4.284
5.154	5.099	5.043	4.984	4.924	4.863	4.801	4.739	4.677
6.882	6.799	6.717	6.634	6.546	6.459	6.367	6.275	6.181
7.241	7.157	7.074	6.99	6.903	6.815	6.728	6.639	6.547
5.131	5.074	5.019	4.963	4.905	4.846	4.788	4.728	4.667
6.323	6.249	6.173	6.099	6.021	5.943	5.865	5.784	5.702
5.255	5.185	5.114	5.042	4.971	4.893	4.817	4.74	4.661
6.422	6.348	6.273	6.195	6.119	6.042	5.963	5.881	5.798
5.961	5.895	5.83	5.762	5.692	5.624	5.553	5.479	5.406
6.203	6.136	6.065	5.994	5.92	5.848	5.772	5.697	5.622
5.66	5.6	5.542	5.481	5.419	5.355	5.292	5.223	5.156
7.203	7.126	7.05	6.968	6.886	6.804	6.719	6.632	6.545
5.455	5.397	5.338	5.279	5.218	5.156	5.093	5.028	4.963
7.717	7.638	7.559	7.478	7.392	7.304	7.214	7.125	7.036
5.314	5.247	5.179	5.111	5.04	4.967	4.893	4.817	4.74
5.612	5.551	5.486	5.422	5.356	5.288	5.221	5.151	5.08
6.367	6.296	6.224	6.152	6.079	6.004	5.927	5.849	5.768
6.248	6.181	6.109	6.037	5.966	5.891	5.818	5.742	5.664
5.59	5.524	5.457	5.391	5.32	5.251	5.18	5.109	5.037
4.59	4.54	4.491	4.441	4.39	4.337	4.283	4.226	4.172
5.328	5.273	5.216	5.159	5.101	5.043	4.984	4.924	4.863
6.912	6.84	6.767	6.693	6.617	6.54	6.462	6.384	6.305
4.406	4.359	4.311	4.262	4.214	4.163	4.113	4.064	4.013
5.505	5.444	5.383	5.319	5.257	5.192	5.126	5.062	4.996
5.969	5.905	5.839	5.772	5.703	5.634	5.566	5.494	5.423
5.217	5.163	5.107	5.05	4.994	4.937	4.878	4.819	4.758
6.396	6.325	6.253	6.178	6.104	6.03	5.954	5.875	5.796
6.093	6.025	5.957	5.888	5.818	5.746	5.672	5.599	5.524
4.364	4.314	4.264	4.211	4.16	4.106	4.052	3.997	3.941
5.101	5.044	4.986	4.929	4.869	4.809	4.748	4.689	4.628
4.573	4.521	4.465	4.41	4.354	4.296	4.238	4.178	4.117
6.122	6.058	5.99	5.922	5.853	5.783	5.708	5.634	5.56
6.587	6.513	6.437	6.359	6.279	6.199	6.119	6.035	5.948
6.618	6.549	6.476	6.401	6.325	6.247	6.167	6.084	5.999
6.316	6.248	6.179	6.105	6.031	5.956	5.879	5.802	5.724
7.037	6.962	6.884	6.802	6.719	6.635	6.549	6.462	6.373
6.21	6.145	6.077	6.007	5.933	5.86	5.784	5.707	5.629
5.576	5.514	5.45	5.386	5.321	5.253	5.183	5.112	5.039
6.831	6.76	6.689	6.615	6.536	6.458	6.377	6.297	6.214
5.192	5.137	5.078	5.019	4.957	4.894	4.829	4.763	4.698

392	394	396	398	400	402	404	406	408
4.224	4.163	4.098	4.036	3.971	3.906	3.839	3.771	3.701
4.612	4.547	4.48	4.412	4.342	4.273	4.2	4.128	4.053
6.087	5.991	5.895	5.796	5.696	5.592	5.487	5.379	5.269
6.453	6.358	6.263	6.164	6.065	5.963	5.857	5.746	5.636
4.605	4.543	4.479	4.415	4.348	4.28	4.211	4.141	4.066
5.619	5.535	5.451	5.363	5.274	5.181	5.086	4.988	4.887
4.583	4.5	4.412	4.319	4.228	4.139	4.044	3.949	3.851
5.717	5.639	5.556	5.471	5.385	5.295	5.206	5.112	5.017
5.332	5.256	5.179	5.102	5.022	4.941	4.855	4.77	4.684
5.542	5.466	5.384	5.302	5.215	5.129	5.041	4.949	4.855
5.088	5.017	4.947	4.874	4.802	4.726	4.647	4.569	4.489
6.458	6.367	6.277	6.184	6.089	5.992	5.894	5.794	5.692
4.896	4.828	4.76	4.691	4.619	4.545	4.47	4.393	4.314
6.943	6.851	6.755	6.658	6.558	6.456	6.353	6.247	6.139
4.663	4.581	4.495	4.413	4.323	4.233	4.138	4.04	3.944
5.009	4.936	4.86	4.783	4.705	4.626	4.546	4.462	4.38
5.688	5.607	5.526	5.44	5.354	5.265	5.175	5.08	4.982
5.587	5.507	5.425	5.345	5.261	5.176	5.088	4.998	4.905
4.964	4.89	4.812	4.733	4.649	4.564	4.475	4.385	4.292
4.114	4.058	3.998	3.938	3.879	3.816	3.752	3.687	3.62
4.801	4.738	4.673	4.606	4.537	4.466	4.395	4.324	4.25
6.226	6.147	6.064	5.981	5.896	5.808	5.719	5.626	5.532
3.961	3.907	3.855	3.802	3.748	3.691	3.635	3.576	3.515
4.928	4.858	4.786	4.712	4.639	4.562	4.486	4.407	4.329
5.35	5.275	5.195	5.116	5.034	4.951	4.865	4.776	4.687
4.697	4.636	4.574	4.509	4.443	4.377	4.307	4.237	4.165
5.716	5.634	5.55	5.462	5.374	5.286	5.192	5.1	5.003
5.447	5.37	5.291	5.209	5.124	5.039	4.951	4.862	4.772
3.884	3.826	3.766	3.705	3.645	3.583	3.52	3.455	3.391
4.564	4.498	4.431	4.365	4.296	4.226	4.154	4.081	4.005
4.055	3.992	3.928	3.863	3.797	3.729	3.658	3.585	3.51
5.482	5.403	5.325	5.244	5.162	5.08	4.995	4.908	4.821
5.862	5.775	5.688	5.6	5.51	5.416	5.32	5.222	5.119
5.913	5.827	5.739	5.653	5.565	5.473	5.379	5.285	5.188
5.645	5.565	5.481	5.398	5.313	5.227	5.14	5.051	4.959
6.284	6.192	6.101	6.007	5.912	5.811	5.708	5.602	5.493
5.549	5.47	5.393	5.312	5.229	5.143	5.057	4.966	4.872
4.967	4.893	4.821	4.745	4.669	4.593	4.514	4.435	4.351
6.129	6.044	5.958	5.87	5.779	5.686	5.593	5.495	5.393
4.631	4.564	4.495	4.427	4.357	4.284	4.211	4.135	4.059

410	412	414	416	418	420	422	424	426
3.63	3.556	3.481	3.403	3.323	3.239	3.156	3.071	2.986
3.977	3.896	3.815	3.73	3.644	3.555	3.462	3.369	3.271
5.16	5.046	4.927	4.805	4.679	4.55	4.418	4.283	4.152
5.521	5.402	5.28	5.159	5.034	4.904	4.775	4.648	4.523
3.991	3.913	3.833	3.75	3.668	3.581	3.496	3.41	3.324
4.783	4.674	4.566	4.454	4.343	4.229	4.113	3.998	3.886
3.752	3.652	3.547	3.442	3.339	3.236	3.136	3.038	2.944
4.921	4.819	4.715	4.607	4.496	4.382	4.265	4.146	4.027
4.594	4.5	4.404	4.303	4.2	4.097	3.994	3.885	3.779
4.76	4.662	4.559	4.455	4.348	4.24	4.128	4.018	3.906
4.405	4.318	4.229	4.136	4.041	3.941	3.841	3.736	3.633
5.585	5.477	5.369	5.264	5.155	5.04	4.914	4.785	4.654
4.233	4.15	4.063	3.973	3.881	3.784	3.687	3.586	3.484
6.027	5.911	5.792	5.669	5.544	5.411	5.275	5.138	4.997
3.843	3.742	3.64	3.537	3.438	3.338	3.237	3.141	3.045
4.293	4.203	4.111	4.017	3.918	3.818	3.715	3.61	3.503
4.883	4.778	4.668	4.557	4.442	4.323	4.202	4.077	3.953
4.809	4.712	4.612	4.508	4.402	4.292	4.18	4.065	3.949
4.197	4.096	3.99	3.882	3.772	3.663	3.551	3.435	3.319
3.55	3.478	3.403	3.327	3.248	3.165	3.08	2.993	2.905
4.177	4.103	4.025	3.95	3.874	3.798	3.724	3.649	3.576
5.436	5.34	5.244	5.143	5.043	4.944	4.844	4.743	4.645
3.453	3.391	3.327	3.264	3.2	3.134	3.071	3.008	2.943
4.25	4.169	4.088	4.008	3.927	3.847	3.769	3.695	3.623
4.596	4.504	4.409	4.314	4.22	4.126	4.031	3.939	3.849
4.093	4.018	3.943	3.863	3.787	3.71	3.634	3.56	3.486
4.905	4.807	4.708	4.609	4.508	4.41	4.311	4.216	4.122
4.68	4.587	4.495	4.401	4.308	4.217	4.126	4.036	3.951
3.324	3.257	3.189	3.124	3.06	2.995	2.934	2.874	2.817
3.93	3.853	3.771	3.69	3.609	3.528	3.444	3.361	3.281
3.431	3.35	3.265	3.178	3.088	2.995	2.901	2.806	2.709
4.729	4.632	4.534	4.437	4.334	4.226	4.114	4	3.883
5.01	4.898	4.785	4.67	4.553	4.432	4.308	4.182	4.056
5.085	4.979	4.869	4.755	4.638	4.515	4.39	4.26	4.127
4.865	4.768	4.664	4.558	4.446	4.33	4.209	4.086	3.96
5.382	5.267	5.148	5.021	4.892	4.757	4.617	4.476	4.333
4.776	4.676	4.573	4.464	4.351	4.233	4.11	3.987	3.859
4.267	4.18	4.091	4.002	3.907	3.809	3.708	3.604	3.495
5.287	5.181	5.07	4.953	4.831	4.705	4.573	4.436	4.3
3.981	3.9	3.817	3.73	3.64	3.547	3.454	3.357	3.259

428	430	432	434	436	438	440	442	444
2.899	2.811	2.728	2.644	2.564	2.486	2.416	2.35	2.287
3.175	3.079	2.985	2.894	2.807	2.725	2.647	2.575	2.506
4.025	3.901	3.779	3.664	3.554	3.45	3.354	3.269	3.19
4.398	4.276	4.16	4.047	3.943	3.845	3.755	3.673	3.596
3.239	3.155	3.074	2.995	2.92	2.848	2.781	2.72	2.663
3.776	3.666	3.561	3.461	3.366	3.277	3.195	3.117	3.048
2.857	2.776	2.699	2.632	2.569	2.512	2.462	2.416	2.376
3.907	3.79	3.672	3.562	3.454	3.352	3.255	3.164	3.082
3.673	3.57	3.468	3.371	3.278	3.191	3.109	3.032	2.96
3.795	3.685	3.582	3.481	3.383	3.291	3.204	3.124	3.05
3.528	3.423	3.316	3.213	3.113	3.018	2.925	2.838	2.757
4.518	4.379	4.241	4.106	3.974	3.848	3.727	3.616	3.512
3.379	3.272	3.165	3.063	2.962	2.863	2.771	2.683	2.6
4.854	4.713	4.576	4.441	4.309	4.183	4.066	3.957	3.854
2.952	2.865	2.779	2.7	2.627	2.558	2.494	2.436	2.383
3.398	3.292	3.189	3.09	2.996	2.905	2.82	2.744	2.673
3.827	3.703	3.584	3.469	3.36	3.258	3.164	3.077	2.997
3.832	3.714	3.595	3.483	3.374	3.267	3.168	3.076	2.991
3.209	3.1	2.997	2.899	2.808	2.723	2.648	2.577	2.513
2.817	2.728	2.638	2.551	2.465	2.384	2.303	2.229	2.161
3.504	3.437	3.37	3.305	3.244	3.188	3.135	3.085	3.039
4.548	4.453	4.361	4.277	4.202	4.129	4.058	3.992	3.93
2.881	2.821	2.762	2.707	2.655	2.607	2.56	2.517	2.478
3.553	3.488	3.425	3.367	3.313	3.263	3.216	3.173	3.135
3.763	3.681	3.601	3.528	3.46	3.398	3.339	3.287	3.239
3.415	3.347	3.282	3.219	3.161	3.108	3.056	3.011	2.967
4.032	3.947	3.863	3.786	3.712	3.642	3.579	3.519	3.465
3.87	3.791	3.717	3.647	3.582	3.523	3.468	3.418	3.37
2.76	2.705	2.657	2.609	2.564	2.523	2.486	2.45	2.419
3.2	3.122	3.046	2.975	2.907	2.845	2.786	2.731	2.681
2.612	2.513	2.418	2.328	2.242	2.161	2.084	2.015	1.953
3.767	3.651	3.536	3.417	3.303	3.189	3.08	2.976	2.88
3.929	3.804	3.681	3.562	3.45	3.343	3.243	3.151	3.067
3.996	3.865	3.733	3.605	3.48	3.358	3.245	3.137	3.038
3.831	3.702	3.575	3.449	3.327	3.209	3.097	2.988	2.889
4.188	4.041	3.897	3.758	3.627	3.498	3.379	3.269	3.167
3.73	3.601	3.469	3.341	3.217	3.097	2.984	2.878	2.781
3.387	3.278	3.168	3.06	2.954	2.849	2.748	2.651	2.562
4.162	4.024	3.885	3.746	3.614	3.488	3.371	3.259	3.156
3.159	3.059	2.957	2.857	2.757	2.66	2.569	2.482	2.4

446	448	450	452	454	456	458	460	462
2.231	2.179	2.134	2.093	2.055	2.022	1.994	1.967	1.944
2.444	2.386	2.335	2.289	2.248	2.212	2.178	2.15	2.123
3.117	3.052	2.994	2.942	2.896	2.855	2.817	2.784	2.757
3.525	3.459	3.399	3.344	3.295	3.248	3.206	3.168	3.132
2.61	2.562	2.518	2.479	2.441	2.406	2.376	2.35	2.323
2.984	2.925	2.871	2.822	2.777	2.737	2.702	2.667	2.639
2.34	2.309	2.28	2.255	2.233	2.213	2.197	2.181	2.167
3.005	2.934	2.869	2.809	2.756	2.706	2.659	2.619	2.583
2.893	2.833	2.777	2.726	2.679	2.638	2.598	2.563	2.532
2.982	2.922	2.865	2.815	2.768	2.725	2.685	2.649	2.617
2.681	2.611	2.547	2.488	2.435	2.386	2.343	2.302	2.265
3.417	3.332	3.252	3.182	3.117	3.057	3.004	2.956	2.915
2.523	2.45	2.383	2.322	2.263	2.211	2.164	2.12	2.082
3.763	3.679	3.602	3.531	3.467	3.409	3.357	3.311	3.269
2.335	2.294	2.256	2.224	2.195	2.168	2.146	2.128	2.11
2.608	2.547	2.494	2.445	2.401	2.362	2.327	2.296	2.267
2.924	2.859	2.801	2.748	2.701	2.66	2.623	2.589	2.56
2.912	2.842	2.778	2.72	2.666	2.62	2.578	2.538	2.503
2.456	2.404	2.359	2.317	2.28	2.247	2.218	2.193	2.169
2.096	2.039	1.985	1.935	1.892	1.852	1.817	1.786	1.76
2.996	2.955	2.917	2.882	2.849	2.818	2.79	2.765	2.739
3.87	3.814	3.761	3.713	3.667	3.627	3.587	3.552	3.518
2.442	2.408	2.378	2.349	2.325	2.3	2.277	2.258	2.237
3.099	3.064	3.033	3.006	2.979	2.955	2.931	2.909	2.888
3.196	3.155	3.117	3.082	3.05	3.022	2.995	2.97	2.948
2.926	2.889	2.856	2.825	2.794	2.769	2.744	2.72	2.699
3.414	3.369	3.323	3.285	3.247	3.215	3.185	3.156	3.13
3.327	3.29	3.253	3.221	3.191	3.162	3.135	3.11	3.086
2.389	2.361	2.335	2.312	2.29	2.271	2.254	2.237	2.221
2.635	2.594	2.556	2.522	2.492	2.464	2.439	2.416	2.396
1.895	1.844	1.8	1.759	1.725	1.696	1.67	1.647	1.628
2.788	2.701	2.621	2.548	2.48	2.417	2.363	2.313	2.268
2.989	2.918	2.853	2.794	2.741	2.693	2.649	2.61	2.574
2.946	2.862	2.785	2.717	2.652	2.594	2.54	2.492	2.448
2.797	2.712	2.636	2.566	2.501	2.446	2.396	2.35	2.311
3.075	2.992	2.916	2.848	2.787	2.732	2.682	2.637	2.596
2.692	2.614	2.543	2.482	2.426	2.377	2.334	2.294	2.262
2.479	2.405	2.334	2.27	2.212	2.158	2.11	2.068	2.03
3.061	2.973	2.892	2.818	2.751	2.693	2.639	2.59	2.546
2.324	2.253	2.186	2.127	2.074	2.024	1.98	1.942	1.908

464	466	468	470	472	474	476	478	480
1.922	1.905	1.89	1.876	1.862	1.851	1.838	1.829	1.819
2.099	2.08	2.062	2.045	2.03	2.019	2.006	1.994	1.982
2.73	2.707	2.686	2.668	2.65	2.632	2.616	2.602	2.588
3.099	3.068	3.041	3.016	2.992	2.971	2.952	2.934	2.916
2.301	2.279	2.26	2.243	2.227	2.21	2.195	2.178	2.164
2.612	2.589	2.568	2.548	2.53	2.512	2.498	2.484	2.468
2.154	2.139	2.123	2.109	2.099	2.088	2.077	2.069	2.06
2.548	2.517	2.491	2.465	2.442	2.422	2.402	2.385	2.371
2.504	2.479	2.456	2.436	2.418	2.401	2.385	2.372	2.359
2.588	2.56	2.536	2.514	2.493	2.476	2.459	2.445	2.429
2.234	2.204	2.178	2.154	2.136	2.117	2.1	2.084	2.069
2.876	2.843	2.814	2.789	2.764	2.743	2.725	2.708	2.692
2.046	2.016	1.989	1.964	1.942	1.923	1.906	1.892	1.879
3.232	3.197	3.166	3.138	3.114	3.09	3.071	3.053	3.036
2.094	2.08	2.067	2.054	2.042	2.033	2.022	2.011	2.001
2.244	2.221	2.201	2.183	2.169	2.155	2.143	2.131	2.119
2.532	2.508	2.487	2.469	2.452	2.437	2.421	2.408	2.395
2.472	2.443	2.417	2.397	2.378	2.359	2.342	2.327	2.311
2.15	2.132	2.115	2.101	2.087	2.076	2.064	2.051	2.04
1.736	1.714	1.697	1.68	1.668	1.655	1.644	1.634	1.625
2.716	2.693	2.673	2.653	2.635	2.618	2.601	2.585	2.569
3.487	3.46	3.435	3.41	3.386	3.364	3.342	3.322	3.303
2.221	2.205	2.189	2.175	2.161	2.148	2.137	2.123	2.111
2.869	2.851	2.833	2.816	2.8	2.784	2.767	2.751	2.735
2.927	2.908	2.889	2.872	2.855	2.839	2.823	2.81	2.794
2.678	2.659	2.642	2.624	2.609	2.592	2.577	2.561	2.546
3.104	3.081	3.059	3.039	3.018	3.001	2.983	2.965	2.948
3.064	3.044	3.024	3.005	2.988	2.969	2.951	2.935	2.917
2.207	2.193	2.179	2.165	2.152	2.14	2.129	2.116	2.104
2.375	2.358	2.339	2.324	2.31	2.296	2.282	2.27	2.257
1.612	1.597	1.584	1.574	1.564	1.555	1.545	1.534	1.525
2.228	2.191	2.157	2.128	2.102	2.078	2.056	2.04	2.022
2.543	2.515	2.49	2.468	2.448	2.43	2.412	2.397	2.381
2.409	2.374	2.34	2.312	2.286	2.263	2.242	2.224	2.207
2.275	2.243	2.218	2.196	2.176	2.157	2.141	2.127	2.114
2.56	2.528	2.499	2.474	2.451	2.431	2.414	2.396	2.38
2.234	2.208	2.187	2.169	2.152	2.137	2.123	2.112	2.099
1.997	1.968	1.943	1.922	1.902	1.884	1.87	1.857	1.845
2.51	2.476	2.446	2.42	2.397	2.377	2.358	2.342	2.328
1.879	1.852	1.828	1.809	1.789	1.773	1.759	1.745	1.736

482	484	486	488	490	492	494	496	498
1.809	1.8	1.79	1.781	1.771	1.763	1.755	1.747	1.737
1.973	1.963	1.952	1.943	1.933	1.924	1.915	1.906	1.897
2.573	2.558	2.545	2.531	2.518	2.505	2.491	2.479	2.467
2.899	2.883	2.867	2.852	2.839	2.826	2.813	2.798	2.784
2.149	2.132	2.117	2.101	2.084	2.066	2.048	2.029	2.01
2.456	2.442	2.43	2.418	2.406	2.393	2.381	2.37	2.358
2.051	2.042	2.032	2.023	2.013	2.005	1.994	1.985	1.976
2.357	2.344	2.332	2.319	2.307	2.295	2.285	2.273	2.262
2.347	2.334	2.322	2.311	2.3	2.288	2.277	2.266	2.256
2.416	2.401	2.388	2.377	2.365	2.353	2.34	2.33	2.318
2.056	2.043	2.033	2.022	2.009	2	1.991	1.98	1.969
2.677	2.66	2.648	2.636	2.622	2.609	2.596	2.584	2.57
1.868	1.856	1.844	1.835	1.822	1.814	1.804	1.795	1.786
3.02	3.005	2.992	2.978	2.965	2.951	2.938	2.925	2.912
1.992	1.983	1.973	1.964	1.956	1.946	1.939	1.93	1.922
2.108	2.097	2.086	2.077	2.066	2.057	2.047	2.037	2.029
2.382	2.368	2.357	2.346	2.333	2.322	2.31	2.3	2.289
2.299	2.286	2.273	2.259	2.248	2.234	2.222	2.212	2.2
2.028	2.017	2.006	1.996	1.985	1.974	1.966	1.955	1.947
1.616	1.609	1.601	1.593	1.587	1.578	1.571	1.563	1.557
2.553	2.538	2.523	2.509	2.495	2.481	2.467	2.454	2.441
3.284	3.266	3.248	3.231	3.211	3.195	3.178	3.161	3.146
2.099	2.088	2.077	2.066	2.056	2.044	2.033	2.024	2.013
2.72	2.704	2.69	2.676	2.661	2.647	2.633	2.618	2.606
2.78	2.766	2.752	2.737	2.723	2.709	2.697	2.684	2.672
2.533	2.517	2.503	2.489	2.477	2.465	2.451	2.438	2.427
2.931	2.913	2.898	2.883	2.867	2.853	2.838	2.824	2.809
2.901	2.883	2.868	2.854	2.839	2.823	2.808	2.793	2.779
2.091	2.08	2.067	2.056	2.045	2.035	2.025	2.013	2.003
2.244	2.232	2.22	2.209	2.197	2.185	2.174	2.163	2.153
1.515	1.508	1.5	1.491	1.484	1.475	1.467	1.459	1.453
2.007	1.993	1.981	1.969	1.957	1.946	1.936	1.924	1.915
2.367	2.354	2.342	2.329	2.316	2.304	2.292	2.279	2.268
2.192	2.177	2.164	2.152	2.138	2.127	2.117	2.105	2.096
2.103	2.09	2.079	2.068	2.057	2.046	2.036	2.027	2.019
2.367	2.352	2.341	2.328	2.315	2.304	2.293	2.281	2.27
2.088	2.077	2.064	2.053	2.043	2.033	2.022	2.012	2.002
1.833	1.822	1.811	1.801	1.791	1.781	1.771	1.761	1.753
2.314	2.302	2.288	2.277	2.264	2.252	2.24	2.228	2.217
1.724	1.715	1.705	1.695	1.685	1.677	1.667	1.659	1.65

500	502	504	506	508	510	512	514	516
1.731	1.724	1.715	1.708	1.702	1.694	1.687	1.681	1.674
1.889	1.881	1.872	1.863	1.857	1.849	1.842	1.834	1.828
2.455	2.444	2.431	2.419	2.408	2.397	2.386	2.377	2.366
2.772	2.759	2.746	2.735	2.723	2.711	2.699	2.687	2.676
1.992	1.973	1.954	1.936	1.916	1.9	1.883	1.868	1.851
2.348	2.337	2.327	2.316	2.305	2.297	2.287	2.278	2.268
1.969	1.959	1.952	1.942	1.936	1.93	1.922	1.916	1.909
2.251	2.239	2.23	2.22	2.21	2.201	2.191	2.182	2.173
2.245	2.234	2.224	2.214	2.204	2.194	2.185	2.176	2.167
2.308	2.296	2.285	2.276	2.267	2.257	2.247	2.237	2.228
1.959	1.95	1.939	1.932	1.924	1.916	1.907	1.899	1.89
2.558	2.546	2.534	2.521	2.511	2.499	2.488	2.478	2.468
1.778	1.769	1.761	1.753	1.744	1.737	1.728	1.721	1.713
2.9	2.887	2.875	2.864	2.852	2.839	2.83	2.819	2.808
1.916	1.908	1.901	1.893	1.886	1.879	1.872	1.866	1.86
2.02	2.01	2.002	1.992	1.985	1.976	1.968	1.96	1.953
2.278	2.268	2.258	2.248	2.238	2.229	2.22	2.211	2.202
2.189	2.177	2.169	2.158	2.148	2.138	2.127	2.119	2.109
1.937	1.928	1.919	1.911	1.903	1.894	1.885	1.876	1.869
1.55	1.543	1.535	1.53	1.523	1.517	1.511	1.505	1.499
2.428	2.416	2.404	2.391	2.38	2.367	2.358	2.346	2.336
3.129	3.114	3.1	3.085	3.071	3.057	3.044	3.031	3.017
2.005	1.995	1.985	1.976	1.967	1.959	1.949	1.94	1.932
2.593	2.581	2.568	2.555	2.543	2.532	2.519	2.507	2.496
2.661	2.648	2.638	2.626	2.615	2.606	2.596	2.584	2.574
2.414	2.401	2.389	2.377	2.367	2.355	2.344	2.334	2.324
2.795	2.78	2.769	2.756	2.743	2.73	2.717	2.706	2.694
2.766	2.753	2.739	2.726	2.713	2.7	2.688	2.676	2.666
1.992	1.984	1.973	1.965	1.956	1.946	1.938	1.93	1.922
2.143	2.132	2.121	2.114	2.104	2.094	2.084	2.077	2.069
1.445	1.44	1.433	1.425	1.419	1.412	1.406	1.401	1.394
1.903	1.893	1.884	1.875	1.865	1.856	1.847	1.839	1.831
2.257	2.245	2.234	2.224	2.214	2.204	2.193	2.183	2.173
2.084	2.075	2.064	2.053	2.043	2.033	2.024	2.014	2.004
2.009	1.999	1.989	1.98	1.972	1.962	1.954	1.947	1.938
2.258	2.247	2.237	2.228	2.217	2.206	2.196	2.186	2.177
1.992	1.983	1.973	1.963	1.955	1.947	1.938	1.93	1.923
1.744	1.735	1.727	1.719	1.711	1.702	1.694	1.687	1.679
2.207	2.196	2.186	2.176	2.167	2.157	2.148	2.138	2.129
1.641	1.632	1.622	1.615	1.607	1.601	1.594	1.585	1.578

518	520	522	524	526	528	530	532	534
1.667	1.66	1.654	1.647	1.641	1.636	1.63	1.624	1.618
1.821	1.812	1.806	1.799	1.793	1.785	1.779	1.774	1.768
2.356	2.347	2.337	2.327	2.317	2.31	2.301	2.292	2.284
2.667	2.655	2.646	2.635	2.625	2.615	2.606	2.597	2.588
1.836	1.824	1.811	1.8	1.788	1.779	1.769	1.761	1.753
2.258	2.25	2.241	2.233	2.224	2.217	2.208	2.201	2.194
1.9	1.895	1.888	1.882	1.876	1.871	1.865	1.858	1.852
2.164	2.155	2.147	2.139	2.13	2.123	2.114	2.107	2.099
2.159	2.151	2.141	2.133	2.126	2.118	2.11	2.103	2.095
2.219	2.21	2.2	2.193	2.184	2.176	2.169	2.16	2.154
1.883	1.875	1.866	1.859	1.853	1.845	1.838	1.832	1.825
2.456	2.447	2.437	2.427	2.417	2.407	2.399	2.391	2.382
1.707	1.699	1.691	1.684	1.678	1.672	1.665	1.658	1.652
2.798	2.789	2.779	2.77	2.759	2.751	2.741	2.732	2.725
1.853	1.848	1.842	1.835	1.829	1.823	1.817	1.812	1.807
1.946	1.938	1.931	1.925	1.916	1.91	1.903	1.897	1.891
2.194	2.185	2.177	2.17	2.161	2.155	2.146	2.139	2.132
2.1	2.091	2.083	2.073	2.065	2.057	2.049	2.043	2.034
1.86	1.853	1.846	1.839	1.833	1.825	1.819	1.812	1.806
1.493	1.487	1.481	1.476	1.471	1.465	1.46	1.454	1.45
2.326	2.315	2.306	2.296	2.286	2.277	2.268	2.258	2.251
3.005	2.991	2.978	2.967	2.955	2.944	2.933	2.921	2.91
1.925	1.916	1.91	1.903	1.895	1.888	1.881	1.873	1.866
2.485	2.475	2.465	2.454	2.445	2.434	2.425	2.417	2.407
2.564	2.555	2.543	2.535	2.525	2.516	2.508	2.498	2.489
2.314	2.304	2.294	2.285	2.276	2.266	2.258	2.25	2.241
2.682	2.67	2.66	2.648	2.637	2.628	2.618	2.606	2.597
2.654	2.642	2.631	2.621	2.61	2.6	2.591	2.582	2.572
1.912	1.905	1.896	1.889	1.881	1.875	1.866	1.86	1.853
2.06	2.052	2.043	2.036	2.029	2.021	2.013	2.006	1.999
1.386	1.381	1.376	1.37	1.364	1.36	1.354	1.351	1.346
1.821	1.813	1.805	1.798	1.789	1.781	1.774	1.768	1.76
2.164	2.154	2.146	2.137	2.129	2.121	2.113	2.106	2.099
1.995	1.986	1.977	1.968	1.96	1.953	1.945	1.937	1.931
1.93	1.922	1.915	1.907	1.9	1.893	1.886	1.88	1.873
2.168	2.159	2.151	2.142	2.134	2.126	2.119	2.111	2.103
1.914	1.906	1.899	1.892	1.885	1.878	1.871	1.864	1.857
1.672	1.666	1.659	1.652	1.645	1.638	1.632	1.625	1.619
2.12	2.112	2.103	2.094	2.086	2.077	2.07	2.062	2.054
1.571	1.564	1.558	1.551	1.545	1.539	1.533	1.526	1.521

536	538	540	542	544	546	548	550	552
1.614	1.609	1.604	1.598	1.594	1.589	1.584	1.579	1.576
1.762	1.757	1.751	1.746	1.741	1.735	1.731	1.725	1.72
2.275	2.269	2.261	2.253	2.247	2.24	2.233	2.227	2.222
2.578	2.568	2.561	2.553	2.545	2.536	2.528	2.521	2.512
1.746	1.738	1.731	1.726	1.72	1.714	1.708	1.703	1.698
2.187	2.179	2.172	2.165	2.158	2.151	2.144	2.138	2.131
1.848	1.843	1.836	1.832	1.828	1.821	1.816	1.811	1.806
2.092	2.084	2.079	2.072	2.065	2.058	2.051	2.044	2.039
2.088	2.082	2.074	2.067	2.062	2.054	2.049	2.041	2.034
2.147	2.139	2.133	2.126	2.119	2.112	2.106	2.1	2.093
1.819	1.813	1.806	1.799	1.795	1.789	1.782	1.776	1.77
2.373	2.366	2.357	2.35	2.342	2.334	2.325	2.319	2.312
1.645	1.641	1.634	1.628	1.622	1.618	1.612	1.608	1.602
2.716	2.706	2.698	2.691	2.684	2.675	2.668	2.661	2.655
1.801	1.798	1.792	1.787	1.782	1.778	1.773	1.769	1.763
1.884	1.879	1.872	1.866	1.861	1.856	1.851	1.845	1.838
2.126	2.119	2.111	2.106	2.098	2.092	2.087	2.081	2.074
2.026	2.019	2.011	2.006	1.999	1.993	1.986	1.979	1.975
1.8	1.794	1.789	1.783	1.778	1.773	1.766	1.762	1.756
1.444	1.44	1.436	1.431	1.428	1.423	1.418	1.414	1.411
2.242	2.234	2.227	2.219	2.211	2.204	2.196	2.189	2.182
2.9	2.889	2.878	2.87	2.859	2.849	2.84	2.831	2.823
1.859	1.852	1.846	1.841	1.834	1.829	1.823	1.818	1.812
2.399	2.391	2.382	2.374	2.367	2.36	2.351	2.343	2.336
2.481	2.474	2.465	2.458	2.451	2.442	2.434	2.427	2.421
2.233	2.226	2.218	2.211	2.204	2.197	2.188	2.182	2.176
2.588	2.581	2.57	2.562	2.554	2.545	2.538	2.529	2.522
2.563	2.554	2.545	2.536	2.527	2.519	2.511	2.504	2.495
1.846	1.839	1.833	1.826	1.821	1.814	1.808	1.804	1.797
1.993	1.986	1.979	1.973	1.966	1.961	1.954	1.948	1.945
1.341	1.337	1.331	1.327	1.323	1.319	1.313	1.309	1.305
1.754	1.748	1.741	1.736	1.729	1.723	1.717	1.712	1.707
2.091	2.084	2.077	2.07	2.064	2.057	2.05	2.043	2.037
1.922	1.915	1.91	1.903	1.897	1.89	1.883	1.879	1.873
1.866	1.86	1.855	1.849	1.843	1.837	1.832	1.827	1.822
2.094	2.089	2.082	2.075	2.07	2.062	2.056	2.049	2.044
1.851	1.845	1.839	1.833	1.827	1.821	1.815	1.809	1.805
1.614	1.608	1.601	1.597	1.593	1.586	1.581	1.577	1.571
2.047	2.042	2.034	2.028	2.021	2.014	2.008	2.002	1.996
1.515	1.509	1.504	1.499	1.493	1.488	1.484	1.478	1.476

554	556	558	560	562	564	566	568	570
1.571	1.566	1.561	1.557	1.553	1.549	1.545	1.542	1.537
1.716	1.711	1.707	1.702	1.697	1.693	1.688	1.685	1.68
2.215	2.208	2.203	2.195	2.19	2.183	2.178	2.172	2.166
2.505	2.499	2.492	2.484	2.477	2.469	2.462	2.455	2.448
1.693	1.688	1.683	1.678	1.673	1.669	1.663	1.659	1.655
2.125	2.119	2.113	2.107	2.101	2.096	2.09	2.083	2.079
1.802	1.796	1.792	1.788	1.783	1.777	1.774	1.769	1.764
2.032	2.026	2.02	2.016	2.009	2.004	1.999	1.992	1.988
2.03	2.024	2.018	2.013	2.006	2.002	1.997	1.992	1.986
2.088	2.081	2.074	2.069	2.063	2.057	2.052	2.046	2.04
1.765	1.759	1.754	1.749	1.742	1.738	1.734	1.728	1.724
2.305	2.298	2.292	2.285	2.28	2.273	2.265	2.26	2.254
1.597	1.591	1.587	1.581	1.578	1.573	1.568	1.565	1.56
2.647	2.642	2.635	2.628	2.622	2.614	2.608	2.602	2.597
1.759	1.755	1.75	1.746	1.741	1.738	1.733	1.729	1.723
1.834	1.829	1.824	1.818	1.814	1.808	1.804	1.799	1.794
2.069	2.064	2.058	2.053	2.047	2.041	2.035	2.03	2.024
1.968	1.962	1.957	1.952	1.946	1.94	1.934	1.928	1.924
1.75	1.745	1.739	1.734	1.73	1.725	1.722	1.717	1.713
1.406	1.403	1.399	1.395	1.39	1.387	1.383	1.379	1.376
2.176	2.167	2.16	2.155	2.147	2.142	2.136	2.129	2.124
2.814	2.805	2.797	2.789	2.782	2.772	2.764	2.757	2.748
1.808	1.801	1.796	1.791	1.786	1.78	1.775	1.771	1.768
2.328	2.322	2.314	2.308	2.3	2.294	2.288	2.281	2.275
2.414	2.407	2.4	2.394	2.387	2.38	2.374	2.368	2.362
2.168	2.162	2.155	2.15	2.143	2.137	2.13	2.125	2.119
2.513	2.506	2.499	2.492	2.485	2.478	2.471	2.466	2.459
2.487	2.479	2.472	2.465	2.458	2.452	2.446	2.439	2.433
1.792	1.786	1.781	1.776	1.771	1.765	1.76	1.756	1.751
1.937	1.931	1.926	1.92	1.915	1.911	1.906	1.9	1.896
1.301	1.296	1.293	1.29	1.287	1.283	1.28	1.277	1.274
1.701	1.696	1.69	1.685	1.681	1.675	1.67	1.667	1.662
2.032	2.026	2.019	2.015	2.009	2.005	1.999	1.993	1.988
1.866	1.859	1.856	1.849	1.843	1.839	1.833	1.828	1.823
1.816	1.812	1.806	1.802	1.798	1.792	1.788	1.782	1.778
2.038	2.032	2.026	2.02	2.016	2.009	2.004	1.998	1.993
1.799	1.794	1.789	1.784	1.779	1.774	1.769	1.765	1.761
1.567	1.563	1.559	1.554	1.549	1.545	1.54	1.537	1.533
1.991	1.985	1.98	1.973	1.969	1.963	1.958	1.952	1.948
1.471	1.467	1.463	1.458	1.454	1.451	1.447	1.443	1.439

572	574	576	578	580	582	584	586	588
1.533	1.529	1.526	1.523	1.52	1.514	1.51	1.507	1.503
1.676	1.672	1.666	1.664	1.659	1.655	1.652	1.65	1.644
2.16	2.156	2.15	2.144	2.139	2.134	2.129	2.124	2.119
2.442	2.437	2.429	2.424	2.418	2.412	2.405	2.398	2.392
1.65	1.646	1.64	1.636	1.632	1.627	1.623	1.62	1.616
2.072	2.067	2.063	2.057	2.052	2.047	2.042	2.036	2.032
1.759	1.755	1.752	1.748	1.743	1.739	1.735	1.732	1.728
1.982	1.976	1.972	1.966	1.962	1.957	1.952	1.947	1.943
1.981	1.975	1.969	1.966	1.96	1.956	1.952	1.946	1.943
2.036	2.03	2.026	2.019	2.015	2.009	2.003	1.999	1.995
1.719	1.715	1.71	1.705	1.701	1.697	1.694	1.687	1.684
2.249	2.243	2.237	2.231	2.226	2.221	2.216	2.209	2.203
1.555	1.551	1.546	1.543	1.538	1.534	1.53	1.528	1.524
2.59	2.585	2.58	2.574	2.568	2.563	2.558	2.551	2.546
1.719	1.715	1.711	1.706	1.702	1.698	1.694	1.688	1.685
1.789	1.786	1.781	1.776	1.772	1.768	1.763	1.758	1.754
2.02	2.015	2.009	2.005	1.999	1.995	1.99	1.985	1.982
1.919	1.915	1.909	1.905	1.899	1.896	1.89	1.885	1.881
1.709	1.702	1.699	1.695	1.691	1.688	1.684	1.68	1.676
1.374	1.369	1.366	1.362	1.359	1.356	1.353	1.349	1.346
2.117	2.112	2.107	2.101	2.095	2.089	2.084	2.079	2.075
2.743	2.735	2.727	2.72	2.713	2.706	2.699	2.692	2.685
1.763	1.758	1.753	1.748	1.744	1.739	1.735	1.731	1.727
2.268	2.261	2.254	2.251	2.245	2.239	2.233	2.228	2.223
2.357	2.35	2.344	2.337	2.332	2.327	2.321	2.315	2.31
2.113	2.108	2.102	2.097	2.091	2.086	2.081	2.076	2.07
2.453	2.447	2.442	2.434	2.429	2.423	2.417	2.411	2.405
2.428	2.421	2.414	2.408	2.402	2.397	2.392	2.386	2.381
1.746	1.741	1.737	1.731	1.727	1.721	1.717	1.713	1.708
1.892	1.886	1.882	1.878	1.873	1.868	1.863	1.859	1.855
1.27	1.267	1.263	1.26	1.257	1.254	1.25	1.247	1.244
1.657	1.652	1.649	1.644	1.64	1.635	1.631	1.627	1.622
1.982	1.977	1.972	1.969	1.962	1.957	1.953	1.948	1.942
1.819	1.814	1.808	1.804	1.8	1.795	1.791	1.786	1.782
1.774	1.77	1.766	1.762	1.756	1.753	1.748	1.745	1.741
1.989	1.983	1.979	1.975	1.967	1.964	1.96	1.955	1.95
1.757	1.752	1.748	1.743	1.739	1.734	1.729	1.725	1.722
1.53	1.525	1.521	1.518	1.514	1.51	1.506	1.503	1.497
1.94	1.936	1.929	1.925	1.922	1.915	1.912	1.908	1.902
1.435	1.431	1.427	1.424	1.42	1.417	1.414	1.41	1.406

590	592	594	596	598	600	602	604	606
1.5	1.496	1.493	1.489	1.484	1.481	1.477	1.474	1.47
1.64	1.637	1.632	1.63	1.625	1.622	1.617	1.612	1.61
2.114	2.109	2.105	2.1	2.094	2.09	2.084	2.079	2.074
2.387	2.381	2.375	2.37	2.363	2.358	2.352	2.347	2.341
1.613	1.609	1.605	1.602	1.597	1.593	1.59	1.586	1.583
2.027	2.023	2.019	2.013	2.008	2.003	1.998	1.994	1.989
1.725	1.72	1.717	1.713	1.709	1.706	1.702	1.697	1.693
1.938	1.933	1.93	1.925	1.919	1.915	1.909	1.905	1.901
1.938	1.933	1.928	1.923	1.918	1.913	1.909	1.905	1.9
1.989	1.985	1.98	1.976	1.972	1.966	1.96	1.956	1.952
1.68	1.676	1.672	1.669	1.665	1.66	1.656	1.651	1.647
2.198	2.193	2.187	2.182	2.177	2.171	2.165	2.16	2.154
1.52	1.516	1.513	1.509	1.504	1.5	1.497	1.493	1.489
2.541	2.536	2.532	2.526	2.52	2.513	2.507	2.503	2.497
1.681	1.676	1.672	1.667	1.665	1.659	1.655	1.651	1.647
1.749	1.744	1.741	1.737	1.732	1.728	1.722	1.719	1.715
1.977	1.971	1.967	1.962	1.957	1.953	1.948	1.943	1.939
1.875	1.872	1.867	1.862	1.857	1.854	1.848	1.844	1.838
1.672	1.669	1.666	1.662	1.658	1.654	1.648	1.644	1.639
1.343	1.34	1.336	1.333	1.33	1.326	1.323	1.32	1.317
2.069	2.063	2.058	2.053	2.048	2.043	2.039	2.035	2.031
2.677	2.672	2.666	2.66	2.653	2.646	2.639	2.633	2.627
1.722	1.718	1.714	1.711	1.708	1.702	1.698	1.694	1.691
2.216	2.212	2.207	2.203	2.197	2.191	2.186	2.181	2.178
2.304	2.3	2.294	2.288	2.284	2.278	2.273	2.268	2.263
2.064	2.06	2.055	2.05	2.044	2.04	2.034	2.03	2.026
2.399	2.394	2.388	2.382	2.375	2.37	2.365	2.36	2.354
2.374	2.369	2.363	2.357	2.352	2.347	2.342	2.335	2.331
1.704	1.699	1.696	1.692	1.688	1.683	1.679	1.674	1.671
1.851	1.846	1.842	1.838	1.833	1.828	1.825	1.82	1.816
1.242	1.239	1.237	1.233	1.231	1.23	1.226	1.224	1.22
1.618	1.614	1.61	1.607	1.602	1.598	1.594	1.591	1.587
1.937	1.933	1.929	1.924	1.919	1.915	1.91	1.906	1.9
1.778	1.774	1.768	1.765	1.761	1.756	1.752	1.747	1.744
1.736	1.732	1.728	1.724	1.719	1.715	1.711	1.708	1.704
1.945	1.94	1.936	1.932	1.927	1.923	1.918	1.913	1.908
1.718	1.714	1.71	1.706	1.702	1.697	1.692	1.69	1.686
1.494	1.491	1.487	1.483	1.48	1.476	1.471	1.468	1.464
1.898	1.893	1.889	1.883	1.879	1.875	1.872	1.866	1.862
1.401	1.399	1.396	1.392	1.388	1.384	1.382	1.379	1.376

608	610	612	614	616	618	620	622	624
1.466	1.463	1.461	1.458	1.454	1.453	1.449	1.446	1.443
1.607	1.602	1.6	1.595	1.594	1.59	1.587	1.581	1.58
2.07	2.066	2.06	2.056	2.052	2.047	2.043	2.039	2.035
2.335	2.331	2.325	2.32	2.315	2.311	2.305	2.302	2.297
1.58	1.576	1.573	1.57	1.567	1.564	1.562	1.557	1.555
1.985	1.98	1.977	1.972	1.969	1.965	1.96	1.956	1.95
1.69	1.687	1.683	1.679	1.674	1.672	1.668	1.665	1.661
1.897	1.892	1.886	1.882	1.878	1.874	1.869	1.866	1.864
1.896	1.892	1.888	1.885	1.881	1.877	1.873	1.869	1.865
1.948	1.943	1.939	1.935	1.93	1.926	1.923	1.919	1.915
1.642	1.639	1.636	1.632	1.628	1.625	1.62	1.617	1.614
2.151	2.146	2.14	2.135	2.13	2.127	2.121	2.118	2.113
1.484	1.482	1.48	1.476	1.472	1.468	1.467	1.463	1.461
2.493	2.485	2.483	2.477	2.473	2.468	2.463	2.458	2.453
1.643	1.639	1.635	1.632	1.629	1.627	1.624	1.619	1.618
1.711	1.707	1.702	1.698	1.695	1.691	1.688	1.684	1.681
1.934	1.93	1.926	1.922	1.918	1.915	1.912	1.906	1.903
1.834	1.829	1.826	1.823	1.819	1.815	1.811	1.808	1.804
1.636	1.633	1.628	1.625	1.622	1.619	1.616	1.612	1.61
1.314	1.312	1.309	1.307	1.304	1.3	1.298	1.297	1.294
2.025	2.022	2.018	2.013	2.01	2.007	2.002	1.998	1.994
2.62	2.613	2.608	2.602	2.595	2.59	2.585	2.579	2.572
1.687	1.685	1.681	1.677	1.674	1.669	1.667	1.664	1.66
2.173	2.169	2.165	2.16	2.156	2.151	2.147	2.141	2.137
2.258	2.254	2.249	2.244	2.24	2.234	2.23	2.224	2.221
2.02	2.015	2.011	2.006	2.002	1.997	1.995	1.989	1.985
2.348	2.344	2.339	2.336	2.33	2.324	2.318	2.315	2.308
2.326	2.321	2.316	2.311	2.305	2.301	2.296	2.291	2.287
1.666	1.663	1.66	1.657	1.653	1.65	1.645	1.644	1.641
1.811	1.808	1.802	1.799	1.795	1.791	1.787	1.783	1.779
1.217	1.212	1.208	1.206	1.205	1.203	1.199	1.196	1.196
1.583	1.579	1.575	1.572	1.568	1.565	1.561	1.557	1.554
1.896	1.892	1.888	1.883	1.879	1.875	1.872	1.868	1.865
1.739	1.735	1.731	1.726	1.724	1.719	1.714	1.709	1.707
1.701	1.698	1.694	1.689	1.686	1.682	1.678	1.675	1.671
1.905	1.9	1.895	1.891	1.887	1.883	1.879	1.873	1.871
1.682	1.677	1.674	1.671	1.667	1.664	1.66	1.655	1.652
1.461	1.458	1.456	1.451	1.448	1.446	1.441	1.437	1.435
1.858	1.853	1.848	1.844	1.841	1.836	1.832	1.828	1.823
1.373	1.37	1.366	1.362	1.36	1.356	1.353	1.352	1.347

626	628	630	632	634	636	638	640	642
1.439	1.436	1.433	1.43	1.426	1.424	1.421	1.419	1.416
1.577	1.573	1.57	1.567	1.563	1.559	1.557	1.554	1.55
2.03	2.026	2.023	2.02	2.015	2.011	2.006	2.003	1.998
2.292	2.287	2.282	2.278	2.274	2.269	2.264	2.26	2.255
1.551	1.547	1.545	1.542	1.537	1.535	1.532	1.528	1.525
1.947	1.944	1.94	1.936	1.932	1.929	1.926	1.922	1.918
1.658	1.655	1.65	1.645	1.642	1.64	1.637	1.633	1.63
1.859	1.855	1.852	1.849	1.846	1.842	1.838	1.834	1.831
1.862	1.856	1.855	1.851	1.846	1.843	1.839	1.836	1.832
1.911	1.908	1.902	1.898	1.893	1.889	1.886	1.882	1.878
1.611	1.607	1.604	1.6	1.596	1.593	1.59	1.587	1.585
2.109	2.104	2.099	2.096	2.09	2.086	2.082	2.077	2.072
1.457	1.453	1.45	1.447	1.444	1.44	1.437	1.434	1.431
2.45	2.444	2.44	2.435	2.431	2.427	2.423	2.418	2.414
1.615	1.612	1.608	1.607	1.604	1.601	1.597	1.594	1.592
1.677	1.673	1.67	1.666	1.662	1.658	1.656	1.651	1.647
1.898	1.895	1.89	1.887	1.882	1.879	1.874	1.871	1.868
1.799	1.795	1.792	1.788	1.784	1.781	1.777	1.772	1.769
1.607	1.605	1.602	1.598	1.594	1.592	1.588	1.584	1.582
1.292	1.288	1.286	1.283	1.28	1.277	1.276	1.273	1.27
1.991	1.986	1.982	1.978	1.973	1.969	1.966	1.961	1.956
2.565	2.559	2.555	2.549	2.543	2.538	2.533	2.529	2.523
1.656	1.652	1.65	1.646	1.641	1.638	1.632	1.631	1.628
2.133	2.128	2.123	2.119	2.114	2.11	2.105	2.103	2.097
2.216	2.211	2.207	2.203	2.198	2.193	2.19	2.187	2.181
1.98	1.976	1.972	1.969	1.963	1.959	1.956	1.951	1.948
2.305	2.3	2.295	2.291	2.287	2.281	2.276	2.273	2.267
2.282	2.277	2.274	2.268	2.264	2.258	2.254	2.25	2.245
1.636	1.632	1.63	1.625	1.622	1.618	1.615	1.613	1.61
1.776	1.772	1.768	1.764	1.761	1.756	1.754	1.751	1.746
1.193	1.19	1.185	1.184	1.181	1.177	1.175	1.173	1.172
1.55	1.546	1.544	1.54	1.537	1.534	1.53	1.527	1.523
1.861	1.856	1.853	1.849	1.845	1.84	1.836	1.833	1.829
1.702	1.698	1.695	1.692	1.688	1.684	1.681	1.677	1.672
1.666	1.663	1.661	1.656	1.654	1.65	1.646	1.644	1.639
1.866	1.862	1.858	1.852	1.849	1.846	1.842	1.838	1.834
1.648	1.644	1.641	1.637	1.634	1.63	1.625	1.622	1.619
1.431	1.429	1.426	1.421	1.419	1.416	1.412	1.41	1.407
1.819	1.816	1.812	1.808	1.804	1.799	1.796	1.793	1.789
1.344	1.341	1.338	1.336	1.332	1.33	1.327	1.324	1.32

644	646	648	650	652	654	656	658	660
1.412	1.409	1.406	1.403	1.401	1.397	1.393	1.39	1.387
1.547	1.544	1.541	1.537	1.534	1.533	1.528	1.526	1.523
1.993	1.989	1.986	1.982	1.979	1.975	1.971	1.968	1.963
2.251	2.247	2.242	2.237	2.233	2.228	2.224	2.22	2.215
1.522	1.519	1.516	1.512	1.509	1.506	1.503	1.5	1.498
1.915	1.91	1.906	1.903	1.9	1.896	1.893	1.889	1.886
1.627	1.624	1.621	1.618	1.615	1.612	1.61	1.607	1.602
1.828	1.824	1.82	1.817	1.812	1.809	1.805	1.8	1.797
1.829	1.825	1.821	1.816	1.813	1.811	1.808	1.804	1.798
1.873	1.87	1.865	1.861	1.858	1.853	1.849	1.845	1.841
1.581	1.578	1.574	1.57	1.566	1.564	1.56	1.555	1.553
2.068	2.064	2.059	2.055	2.051	2.046	2.042	2.037	2.034
1.429	1.425	1.421	1.419	1.416	1.413	1.41	1.407	1.404
2.41	2.404	2.401	2.396	2.391	2.387	2.382	2.378	2.373
1.588	1.585	1.582	1.58	1.578	1.575	1.572	1.57	1.568
1.644	1.641	1.637	1.633	1.63	1.627	1.624	1.62	1.616
1.863	1.86	1.855	1.852	1.848	1.844	1.839	1.835	1.833
1.767	1.763	1.759	1.755	1.751	1.748	1.744	1.741	1.737
1.578	1.575	1.572	1.568	1.567	1.562	1.561	1.558	1.554
1.269	1.266	1.263	1.26	1.257	1.255	1.252	1.248	1.246
1.953	1.949	1.946	1.942	1.938	1.935	1.931	1.926	1.922
2.518	2.514	2.508	2.503	2.498	2.492	2.488	2.482	2.477
1.624	1.621	1.618	1.615	1.612	1.61	1.608	1.604	1.601
2.095	2.09	2.086	2.082	2.077	2.074	2.071	2.066	2.063
2.176	2.173	2.168	2.164	2.16	2.157	2.151	2.147	2.143
1.945	1.94	1.936	1.932	1.928	1.925	1.921	1.916	1.913
2.264	2.26	2.255	2.25	2.247	2.243	2.237	2.233	2.228
2.242	2.237	2.231	2.227	2.224	2.22	2.216	2.21	2.207
1.606	1.603	1.599	1.597	1.594	1.593	1.591	1.588	1.585
1.742	1.739	1.735	1.732	1.728	1.725	1.722	1.718	1.715
1.168	1.163	1.165	1.159	1.156	1.155	1.153	1.15	1.147
1.52	1.517	1.513	1.51	1.507	1.503	1.5	1.497	1.494
1.824	1.82	1.817	1.813	1.809	1.806	1.801	1.796	1.794
1.669	1.667	1.662	1.658	1.655	1.651	1.648	1.644	1.641
1.636	1.632	1.629	1.627	1.622	1.62	1.616	1.612	1.609
1.829	1.826	1.821	1.818	1.815	1.811	1.806	1.804	1.799
1.615	1.612	1.61	1.604	1.603	1.598	1.594	1.59	1.587
1.403	1.4	1.399	1.395	1.393	1.389	1.386	1.383	1.38
1.785	1.781	1.777	1.773	1.769	1.765	1.762	1.759	1.754
1.317	1.314	1.312	1.31	1.307	1.305	1.302	1.299	1.295

662	664	666	668	670	672	674	676	678
1.385	1.382	1.379	1.376	1.372	1.369	1.366	1.365	1.362
1.518	1.515	1.513	1.509	1.506	1.503	1.498	1.497	1.493
1.959	1.955	1.951	1.948	1.944	1.939	1.937	1.933	1.929
2.211	2.206	2.201	2.197	2.193	2.19	2.186	2.183	2.179
1.495	1.492	1.49	1.487	1.483	1.48	1.478	1.475	1.471
1.883	1.879	1.876	1.872	1.868	1.865	1.862	1.858	1.855
1.601	1.598	1.594	1.593	1.588	1.585	1.583	1.581	1.578
1.793	1.79	1.785	1.782	1.778	1.775	1.771	1.768	1.765
1.795	1.791	1.788	1.785	1.78	1.777	1.774	1.769	1.766
1.837	1.833	1.828	1.825	1.821	1.818	1.813	1.809	1.806
1.548	1.543	1.537	1.535	1.533	1.529	1.527	1.524	1.521
2.03	2.025	2.02	2.016	2.012	2.007	2.003	2	1.996
1.4	1.397	1.393	1.391	1.389	1.385	1.383	1.38	1.377
2.369	2.364	2.36	2.356	2.351	2.347	2.344	2.34	2.336
1.564	1.561	1.559	1.555	1.554	1.55	1.546	1.544	1.541
1.612	1.609	1.605	1.601	1.598	1.594	1.591	1.587	1.584
1.829	1.825	1.821	1.818	1.815	1.811	1.808	1.804	1.801
1.732	1.729	1.725	1.721	1.717	1.714	1.71	1.707	1.704
1.551	1.548	1.545	1.544	1.541	1.537	1.534	1.531	1.527
1.243	1.24	1.239	1.236	1.233	1.23	1.227	1.225	1.222
1.918	1.914	1.911	1.907	1.904	1.9	1.898	1.893	1.89
2.472	2.467	2.462	2.458	2.452	2.448	2.441	2.437	2.432
1.597	1.594	1.591	1.588	1.585	1.583	1.581	1.578	1.574
2.059	2.054	2.052	2.047	2.043	2.04	2.036	2.032	2.029
2.139	2.134	2.13	2.126	2.122	2.117	2.114	2.111	2.107
1.909	1.904	1.9	1.898	1.893	1.889	1.886	1.883	1.879
2.224	2.22	2.216	2.211	2.208	2.204	2.2	2.197	2.193
2.204	2.198	2.196	2.191	2.186	2.184	2.178	2.175	2.171
1.583	1.58	1.577	1.575	1.571	1.568	1.565	1.563	1.56
1.709	1.707	1.702	1.699	1.695	1.692	1.689	1.687	1.682
1.145	1.143	1.14	1.137	1.136	1.133	1.132	1.129	1.126
1.491	1.488	1.484	1.48	1.478	1.474	1.471	1.468	1.464
1.789	1.786	1.782	1.779	1.775	1.772	1.768	1.765	1.761
1.637	1.634	1.631	1.627	1.622	1.62	1.615	1.612	1.61
1.607	1.603	1.599	1.597	1.594	1.591	1.587	1.583	1.579
1.795	1.791	1.788	1.784	1.78	1.777	1.772	1.769	1.765
1.584	1.581	1.578	1.575	1.572	1.568	1.566	1.563	1.558
1.377	1.373	1.37	1.367	1.364	1.362	1.36	1.356	1.353
1.75	1.746	1.742	1.739	1.735	1.732	1.728	1.724	1.721
1.292	1.289	1.286	1.284	1.282	1.279	1.277	1.273	1.271

680	682	684	686	688	690	692	694	696
1.359	1.356	1.352	1.349	1.348	1.346	1.343	1.341	1.338
1.491	1.488	1.486	1.481	1.48	1.477	1.474	1.473	1.468
1.925	1.922	1.918	1.914	1.912	1.908	1.904	1.9	1.898
2.174	2.171	2.166	2.163	2.159	2.154	2.153	2.148	2.144
1.469	1.466	1.464	1.462	1.458	1.456	1.453	1.45	1.448
1.852	1.848	1.845	1.842	1.839	1.836	1.832	1.83	1.826
1.574	1.571	1.57	1.567	1.563	1.561	1.558	1.554	1.553
1.761	1.758	1.754	1.751	1.748	1.745	1.742	1.739	1.735
1.763	1.758	1.755	1.752	1.749	1.745	1.741	1.739	1.736
1.804	1.8	1.796	1.792	1.79	1.786	1.782	1.779	1.776
1.518	1.515	1.513	1.51	1.508	1.505	1.502	1.5	1.497
1.992	1.987	1.984	1.98	1.976	1.973	1.97	1.966	1.964
1.375	1.372	1.369	1.366	1.363	1.36	1.357	1.354	1.352
2.332	2.327	2.324	2.32	2.317	2.313	2.31	2.306	2.302
1.54	1.538	1.536	1.532	1.53	1.528	1.526	1.52	1.518
1.575	1.572	1.57	1.567	1.565	1.561	1.558	1.557	1.554
1.791	1.787	1.785	1.782	1.778	1.776	1.774	1.771	1.768
1.7	1.697	1.694	1.685	1.681	1.678	1.675	1.673	1.671
1.525	1.522	1.52	1.518	1.516	1.512	1.508	1.505	1.504
1.22	1.217	1.216	1.212	1.209	1.207	1.206	1.203	1.202
1.887	1.884	1.88	1.876	1.872	1.871	1.866	1.864	1.861
2.429	2.424	2.419	2.415	2.411	2.407	2.402	2.398	2.393
1.573	1.569	1.565	1.563	1.561	1.557	1.554	1.551	1.548
2.024	2.02	2.017	2.014	2.009	2.007	2.005	2	1.997
2.104	2.099	2.095	2.091	2.088	2.084	2.08	2.079	2.074
1.875	1.871	1.867	1.863	1.861	1.857	1.853	1.851	1.846
2.188	2.186	2.181	2.177	2.173	2.169	2.166	2.162	2.159
2.167	2.165	2.161	2.157	2.154	2.15	2.146	2.142	2.14
1.557	1.555	1.553	1.55	1.547	1.544	1.54	1.537	1.536
1.68	1.676	1.672	1.669	1.667	1.665	1.66	1.658	1.654
1.123	1.122	1.12	1.119	1.116	1.115	1.112	1.109	1.108
1.463	1.459	1.458	1.454	1.451	1.447	1.446	1.443	1.44
1.757	1.754	1.751	1.747	1.742	1.739	1.736	1.733	1.731
1.606	1.602	1.6	1.597	1.593	1.59	1.586	1.585	1.581
1.575	1.574	1.571	1.568	1.564	1.562	1.558	1.556	1.554
1.762	1.758	1.754	1.751	1.746	1.744	1.741	1.738	1.734
1.556	1.55	1.548	1.544	1.543	1.54	1.536	1.533	1.53
1.35	1.349	1.344	1.343	1.34	1.337	1.334	1.332	1.329
1.717	1.714	1.711	1.707	1.703	1.7	1.696	1.692	1.689
1.268	1.266	1.262	1.26	1.257	1.256	1.252	1.25	1.249

698	700	702	704	706	708	710	712	714
1.336	1.333	1.332	1.329	1.328	1.325	1.323	1.322	1.319
1.467	1.464	1.461	1.458	1.456	1.453	1.451	1.449	1.447
1.895	1.891	1.888	1.883	1.881	1.879	1.865	1.863	1.861
2.141	2.139	2.136	2.133	2.129	2.126	2.123	2.12	2.117
1.445	1.441	1.44	1.438	1.436	1.433	1.43	1.428	1.426
1.824	1.822	1.819	1.816	1.812	1.81	1.806	1.805	1.802
1.55	1.547	1.545	1.542	1.54	1.538	1.535	1.534	1.53
1.734	1.73	1.728	1.725	1.722	1.721	1.719	1.717	1.714
1.734	1.732	1.728	1.727	1.724	1.721	1.718	1.716	1.712
1.774	1.77	1.767	1.764	1.762	1.758	1.756	1.754	1.751
1.494	1.493	1.49	1.488	1.485	1.483	1.48	1.478	1.477
1.96	1.957	1.955	1.95	1.949	1.945	1.941	1.939	1.936
1.35	1.343	1.34	1.339	1.337	1.335	1.334	1.332	1.33
2.299	2.296	2.292	2.289	2.287	2.273	2.268	2.266	2.263
1.516	1.515	1.512	1.511	1.51	1.508	1.506	1.505	1.504
1.55	1.548	1.547	1.544	1.541	1.538	1.536	1.534	1.531
1.765	1.762	1.76	1.758	1.755	1.752	1.75	1.749	1.746
1.669	1.665	1.662	1.661	1.658	1.655	1.653	1.65	1.648
1.501	1.498	1.495	1.493	1.491	1.488	1.486	1.484	1.482
1.2	1.198	1.196	1.195	1.192	1.19	1.189	1.187	1.185
1.858	1.856	1.852	1.849	1.848	1.845	1.842	1.838	1.837
2.39	2.387	2.382	2.378	2.374	2.371	2.367	2.364	2.36
1.546	1.543	1.541	1.538	1.536	1.534	1.532	1.53	1.527
1.994	1.99	1.987	1.985	1.983	1.979	1.976	1.973	1.971
2.072	2.069	2.064	2.061	2.059	2.056	2.053	2.05	2.047
1.845	1.841	1.838	1.835	1.832	1.83	1.828	1.825	1.822
2.156	2.154	2.15	2.147	2.144	2.141	2.139	2.134	2.131
2.136	2.133	2.13	2.127	2.123	2.12	2.117	2.114	2.111
1.533	1.53	1.528	1.527	1.525	1.523	1.52	1.517	1.516
1.652	1.65	1.647	1.644	1.641	1.638	1.636	1.634	1.63
1.105	1.102	1.1	1.099	1.097	1.096	1.093	1.092	1.09
1.437	1.434	1.433	1.43	1.429	1.424	1.424	1.421	1.419
1.729	1.725	1.723	1.722	1.718	1.715	1.712	1.711	1.708
1.578	1.577	1.574	1.571	1.568	1.566	1.563	1.561	1.558
1.55	1.548	1.546	1.544	1.541	1.538	1.537	1.534	1.531
1.732	1.729	1.727	1.724	1.721	1.717	1.715	1.712	1.709
1.527	1.526	1.521	1.52	1.517	1.514	1.511	1.509	1.507
1.326	1.324	1.323	1.32	1.317	1.316	1.314	1.31	1.309
1.687	1.685	1.681	1.679	1.678	1.674	1.672	1.669	1.665
1.246	1.243	1.242	1.239	1.237	1.235	1.233	1.231	1.229

716	718	720	722	724	726	728	730	732
1.318	1.316	1.314	1.312	1.309	1.307	1.306	1.303	1.302
1.445	1.443	1.441	1.439	1.437	1.434	1.433	1.432	1.43
1.858	1.855	1.853	1.851	1.848	1.845	1.845	1.842	1.839
2.114	2.11	2.109	2.104	2.102	2.1	2.096	2.093	2.089
1.423	1.423	1.42	1.418	1.416	1.413	1.412	1.409	1.408
1.799	1.798	1.795	1.792	1.79	1.786	1.783	1.782	1.779
1.528	1.526	1.524	1.522	1.519	1.517	1.515	1.513	1.511
1.711	1.707	1.707	1.704	1.7	1.698	1.697	1.694	1.692
1.709	1.708	1.704	1.702	1.699	1.698	1.697	1.693	1.691
1.748	1.745	1.743	1.739	1.737	1.735	1.734	1.731	1.728
1.474	1.473	1.47	1.467	1.466	1.465	1.462	1.461	1.458
1.932	1.929	1.926	1.923	1.92	1.92	1.916	1.914	1.905
1.327	1.326	1.324	1.323	1.32	1.319	1.318	1.317	1.315
2.261	2.259	2.256	2.253	2.249	2.246	2.243	2.241	2.237
1.502	1.501	1.501	1.498	1.498	1.495	1.494	1.493	1.49
1.531	1.528	1.526	1.523	1.521	1.518	1.517	1.516	1.513
1.742	1.741	1.738	1.737	1.734	1.732	1.73	1.728	1.725
1.645	1.643	1.643	1.64	1.638	1.635	1.634	1.631	1.63
1.48	1.478	1.477	1.475	1.474	1.471	1.469	1.467	1.465
1.183	1.182	1.18	1.178	1.178	1.175	1.173	1.172	1.17
1.834	1.832	1.829	1.827	1.825	1.822	1.82	1.817	1.815
2.355	2.352	2.351	2.347	2.344	2.341	2.338	2.335	2.332
1.524	1.522	1.52	1.517	1.517	1.513	1.511	1.508	1.508
1.969	1.966	1.963	1.96	1.957	1.955	1.952	1.95	1.948
2.044	2.04	2.038	2.037	2.034	2.03	2.029	2.027	2.024
1.819	1.815	1.813	1.812	1.809	1.807	1.805	1.804	1.801
2.129	2.126	2.123	2.12	2.117	2.114	2.112	2.11	2.106
2.109	2.107	2.103	2.1	2.097	2.094	2.092	2.09	2.087
1.513	1.511	1.51	1.508	1.507	1.504	1.503	1.501	1.5
1.628	1.625	1.623	1.62	1.618	1.615	1.614	1.611	1.61
1.089	1.086	1.086	1.085	1.082	1.082	1.08	1.079	1.078
1.417	1.414	1.413	1.411	1.409	1.407	1.405	1.403	1.4
1.705	1.704	1.7	1.698	1.695	1.693	1.691	1.689	1.687
1.557	1.554	1.552	1.55	1.548	1.544	1.543	1.54	1.538
1.528	1.528	1.526	1.524	1.522	1.52	1.517	1.517	1.515
1.707	1.704	1.702	1.699	1.697	1.696	1.692	1.691	1.689
1.504	1.502	1.5	1.498	1.496	1.494	1.493	1.491	1.488
1.307	1.305	1.303	1.301	1.299	1.296	1.295	1.293	1.292
1.662	1.661	1.658	1.657	1.654	1.651	1.65	1.648	1.645
1.228	1.226	1.223	1.223	1.22	1.218	1.215	1.215	1.213

734	736	738	740	742	744	746	748	750
1.301	1.299	1.298	1.296	1.293	1.292	1.292	1.29	1.288
1.429	1.426	1.424	1.423	1.421	1.419	1.417	1.416	1.414
1.837	1.835	1.833	1.832	1.828	1.826	1.825	1.822	1.819
2.087	2.084	2.072	2.069	2.066	2.064	2.063	2.06	2.058
1.406	1.403	1.402	1.401	1.398	1.396	1.395	1.392	1.391
1.776	1.775	1.773	1.771	1.768	1.764	1.764	1.762	1.758
1.508	1.506	1.503	1.501	1.498	1.496	1.495	1.493	1.491
1.69	1.688	1.687	1.684	1.682	1.679	1.677	1.675	1.672
1.689	1.687	1.684	1.681	1.679	1.677	1.675	1.672	1.671
1.725	1.724	1.722	1.718	1.718	1.715	1.712	1.709	1.707
1.456	1.454	1.453	1.45	1.448	1.447	1.446	1.443	1.441
1.902	1.9	1.899	1.896	1.895	1.892	1.89	1.889	1.886
1.313	1.311	1.31	1.309	1.306	1.305	1.302	1.301	1.3
2.234	2.231	2.227	2.224	2.222	2.217	2.215	2.21	2.207
1.488	1.487	1.485	1.484	1.481	1.48	1.479	1.478	1.475
1.511	1.51	1.507	1.504	1.503	1.501	1.5	1.497	1.496
1.724	1.722	1.719	1.718	1.717	1.714	1.712	1.709	1.708
1.627	1.627	1.624	1.622	1.619	1.618	1.617	1.614	1.613
1.464	1.463	1.462	1.46	1.458	1.455	1.454	1.454	1.453
1.17	1.168	1.166	1.165	1.163	1.162	1.16	1.159	1.158
1.812	1.811	1.808	1.807	1.802	1.801	1.798	1.797	1.794
2.329	2.324	2.322	2.319	2.316	2.314	2.31	2.308	2.305
1.504	1.504	1.503	1.5	1.497	1.496	1.494	1.492	1.49
1.945	1.942	1.94	1.939	1.935	1.933	1.93	1.928	1.926
2.022	2.019	2.017	2.015	2.014	2.01	2.007	2.006	2.003
1.799	1.796	1.794	1.791	1.789	1.786	1.785	1.782	1.78
2.104	2.101	2.099	2.097	2.094	2.093	2.09	2.088	2.086
2.084	2.082	2.08	2.078	2.074	2.072	2.07	2.067	2.065
1.498	1.496	1.494	1.493	1.491	1.488	1.487	1.486	1.483
1.607	1.605	1.602	1.601	1.598	1.597	1.595	1.593	1.59
1.078	1.075	1.074	1.073	1.07	1.069	1.068	1.066	1.065
1.399	1.396	1.394	1.393	1.391	1.389	1.388	1.385	1.383
1.685	1.683	1.681	1.679	1.677	1.675	1.674	1.672	1.669
1.535	1.534	1.534	1.532	1.528	1.528	1.525	1.523	1.523
1.512	1.511	1.509	1.507	1.505	1.504	1.501	1.501	1.498
1.688	1.685	1.682	1.681	1.678	1.677	1.674	1.673	1.671
1.487	1.485	1.483	1.48	1.479	1.477	1.475	1.474	1.471
1.29	1.288	1.286	1.285	1.284	1.282	1.28	1.277	1.277
1.642	1.641	1.638	1.638	1.634	1.632	1.631	1.628	1.626
1.212	1.21	1.209	1.207	1.206	1.204	1.203	1.2	1.199

752	754	756	758	760	762	764	766	768
1.286	1.283	1.282	1.282	1.279	1.279	1.278	1.276	1.274
1.413	1.411	1.409	1.406	1.404	1.403	1.402	1.4	1.399
1.818	1.816	1.814	1.812	1.811	1.809	1.806	1.804	1.802
2.056	2.053	2.05	2.048	2.046	2.045	2.042	2.04	2.037
1.389	1.385	1.385	1.383	1.379	1.378	1.376	1.375	1.373
1.756	1.754	1.752	1.749	1.746	1.744	1.741	1.739	1.737
1.488	1.487	1.485	1.483	1.48	1.478	1.477	1.474	1.473
1.671	1.667	1.667	1.665	1.664	1.662	1.658	1.657	1.655
1.669	1.667	1.664	1.662	1.66	1.658	1.657	1.655	1.652
1.706	1.704	1.701	1.699	1.697	1.695	1.692	1.691	1.689
1.439	1.437	1.434	1.433	1.432	1.43	1.428	1.427	1.424
1.885	1.882	1.881	1.879	1.876	1.875	1.873	1.872	1.871
1.297	1.296	1.295	1.293	1.292	1.289	1.288	1.286	1.285
2.203	2.199	2.195	2.192	2.187	2.182	2.178	2.173	2.17
1.474	1.473	1.471	1.47	1.467	1.465	1.463	1.461	1.461
1.494	1.492	1.49	1.488	1.486	1.484	1.483	1.48	1.479
1.705	1.702	1.701	1.698	1.697	1.695	1.692	1.691	1.688
1.611	1.608	1.606	1.604	1.602	1.6	1.598	1.596	1.594
1.453	1.451	1.451	1.451	1.45	1.446	1.445	1.443	1.443
1.156	1.155	1.154	1.152	1.15	1.149	1.147	1.145	1.145
1.791	1.79	1.788	1.785	1.784	1.782	1.781	1.778	1.777
2.303	2.299	2.295	2.294	2.291	2.287	2.285	2.283	2.28
1.488	1.487	1.484	1.483	1.481	1.478	1.477	1.474	1.473
1.923	1.919	1.918	1.915	1.912	1.91	1.909	1.906	1.905
2	1.997	1.996	1.995	1.991	1.99	1.989	1.985	1.983
1.778	1.775	1.773	1.772	1.769	1.767	1.765	1.763	1.761
2.082	2.081	2.077	2.076	2.073	2.072	2.069	2.067	2.065
2.063	2.062	2.059	2.056	2.053	2.051	2.049	2.046	2.044
1.481	1.481	1.48	1.478	1.476	1.476	1.473	1.47	1.469
1.588	1.585	1.584	1.581	1.581	1.579	1.576	1.574	1.574
1.065	1.063	1.061	1.061	1.059	1.058	1.058	1.056	1.055
1.383	1.381	1.381	1.379	1.377	1.376	1.374	1.372	1.371
1.667	1.664	1.662	1.661	1.659	1.658	1.656	1.655	1.654
1.521	1.518	1.515	1.515	1.513	1.511	1.509	1.507	1.505
1.497	1.495	1.493	1.492	1.49	1.489	1.487	1.485	1.484
1.668	1.667	1.664	1.662	1.661	1.658	1.657	1.655	1.652
1.47	1.469	1.467	1.464	1.463	1.461	1.46	1.459	1.456
1.276	1.273	1.272	1.27	1.269	1.267	1.266	1.265	1.263
1.624	1.622	1.62	1.618	1.616	1.613	1.612	1.61	1.608
1.199	1.196	1.196	1.193	1.192	1.19	1.189	1.188	1.187

770	772	774	776	778	780	782	784	786
1.272	1.271	1.269	1.268	1.266	1.266	1.264	1.263	1.262
1.396	1.395	1.393	1.391	1.391	1.39	1.389	1.386	1.384
1.802	1.798	1.797	1.796	1.794	1.792	1.789	1.788	1.787
2.035	2.033	2.032	2.029	2.027	2.025	2.023	2.02	2.019
1.371	1.369	1.368	1.366	1.365	1.362	1.361	1.36	1.358
1.735	1.732	1.731	1.728	1.725	1.724	1.721	1.719	1.717
1.47	1.468	1.466	1.466	1.463	1.461	1.458	1.457	1.454
1.654	1.653	1.65	1.648	1.646	1.645	1.643	1.641	1.64
1.651	1.648	1.647	1.645	1.644	1.641	1.64	1.637	1.635
1.687	1.685	1.684	1.682	1.679	1.678	1.676	1.675	1.672
1.422	1.421	1.419	1.418	1.416	1.414	1.413	1.41	1.409
1.868	1.866	1.865	1.861	1.858	1.857	1.855	1.852	1.851
1.283	1.282	1.28	1.279	1.278	1.276	1.275	1.273	1.272
2.166	2.161	2.156	2.152	2.148	2.142	2.138	2.133	2.128
1.458	1.457	1.455	1.455	1.453	1.453	1.45	1.448	1.447
1.476	1.474	1.473	1.471	1.47	1.468	1.467	1.464	1.461
1.687	1.685	1.684	1.682	1.679	1.677	1.675	1.674	1.672
1.593	1.591	1.589	1.586	1.585	1.583	1.58	1.578	1.578
1.438	1.437	1.436	1.435	1.434	1.43	1.429	1.428	1.427
1.143	1.14	1.14	1.139	1.138	1.136	1.133	1.132	1.129
1.774	1.774	1.772	1.769	1.767	1.765	1.762	1.76	1.758
2.277	2.275	2.273	2.27	2.268	2.266	2.263	2.26	2.258
1.471	1.47	1.467	1.467	1.464	1.463	1.461	1.46	1.458
1.902	1.899	1.896	1.895	1.895	1.893	1.89	1.888	1.886
1.981	1.979	1.977	1.975	1.973	1.971	1.969	1.965	1.963
1.759	1.758	1.755	1.754	1.751	1.75	1.748	1.745	1.744
2.063	2.061	2.058	2.057	2.054	2.053	2.05	2.049	2.046
2.042	2.04	2.038	2.036	2.034	2.032	2.03	2.029	2.026
1.467	1.466	1.464	1.464	1.463	1.461	1.457	1.457	1.454
1.571	1.568	1.567	1.565	1.563	1.561	1.558	1.557	1.555
1.053	1.052	1.051	1.049	1.049	1.048	1.046	1.045	1.043
1.37	1.368	1.367	1.366	1.364	1.363	1.361	1.36	1.357
1.651	1.65	1.648	1.647	1.644	1.642	1.64	1.638	1.637
1.504	1.503	1.501	1.5	1.498	1.495	1.493	1.491	1.49
1.482	1.48	1.478	1.476	1.474	1.473	1.471	1.471	1.468
1.651	1.65	1.647	1.645	1.644	1.642	1.641	1.638	1.635
1.454	1.452	1.451	1.45	1.449	1.446	1.444	1.443	1.441
1.262	1.262	1.26	1.257	1.257	1.256	1.254	1.252	1.25
1.607	1.603	1.602	1.6	1.598	1.595	1.594	1.593	1.591
1.186	1.183	1.183	1.18	1.179	1.178	1.176	1.175	1.175

788	790	792	794	796	798	800	
1.259	1.259	1.257	1.256	1.255	1.253	1.252	WT1
1.383	1.382	1.38	1.378	1.376	1.376	1.374	WT2
1.785	1.781	1.779	1.779	1.777	1.776	1.774	WT3
2.017	2.015	2.013	2.01	2.008	2.007	2.005	WT4
1.356	1.353	1.352	1.351	1.351	1.348	1.348	WT5
1.715	1.714	1.711	1.709	1.707	1.704	1.704	WT6
1.453	1.451	1.449	1.447	1.444	1.443	1.441	WT7
1.637	1.635	1.633	1.631	1.63	1.628	1.627	WT8
1.634	1.631	1.63	1.628	1.627	1.625	1.623	WT9
1.669	1.668	1.667	1.663	1.661	1.661	1.66	WT10
1.407	1.407	1.406	1.403	1.401	1.4	1.398	WB1
1.849	1.848	1.845	1.843	1.842	1.84	1.838	WB2
1.27	1.269	1.268	1.266	1.266	1.263	1.262	WB3
2.125	2.119	2.115	2.11	2.106	2.102	2.096	WB4
1.445	1.443	1.441	1.44	1.438	1.437	1.435	WB5
1.46	1.459	1.457	1.456	1.453	1.451	1.45	WB6
1.671	1.668	1.667	1.665	1.663	1.661	1.66	WB7
1.575	1.575	1.573	1.57	1.568	1.566	1.564	WB8
1.425	1.424	1.423	1.423	1.421	1.418	1.417	WB9
1.129	1.128	1.127	1.125	1.123	1.123	1.121	WB10
1.757	1.755	1.753	1.751	1.75	1.747	1.745	SL1
2.256	2.254	2.253	2.248	2.245	2.244	2.241	SL2
1.457	1.454	1.454	1.453	1.451	1.449	1.447	SL3
1.885	1.883	1.88	1.878	1.876	1.875	1.872	SL4
1.962	1.96	1.959	1.956	1.955	1.953	1.951	SL5
1.742	1.74	1.739	1.737	1.735	1.732	1.731	SL6
2.045	2.043	2.041	2.039	2.036	2.034	2.032	SL7
2.024	2.022	2.02	2.018	2.015	2.015	2.012	SL8
1.454	1.451	1.45	1.45	1.447	1.446	1.444	SL9
1.553	1.551	1.551	1.55	1.548	1.547	1.545	SL10
1.042	1.041	1.041	1.039	1.037	1.036	1.034	CW1
1.356	1.355	1.353	1.353	1.35	1.349	1.347	CW2
1.635	1.632	1.631	1.63	1.628	1.627	1.625	CW3
1.488	1.487	1.484	1.483	1.483	1.48	1.478	CW4
1.467	1.465	1.464	1.463	1.46	1.459	1.458	CW5
1.634	1.632	1.63	1.63	1.627	1.625	1.624	CW6
1.44	1.438	1.437	1.435	1.434	1.433	1.43	CW7
1.249	1.248	1.246	1.246	1.245	1.243	1.242	CW8
1.59	1.587	1.585	1.584	1.583	1.581	1.58	CW9
1.173	1.171	1.169	1.167	1.166	1.165	1.163	CW10

106	108	110	112	114	116	118	120	122
6.481	6.475	6.468	6.465	6.461	6.452	6.451	6.448	6.442
8.052	8.033	8.024	8.005	7.999	7.983	7.976	7.967	7.958
4.698	4.697	4.692	4.692	4.688	4.686	4.684	4.679	4.678
5.591	5.581	5.579	5.573	5.567	5.564	5.558	5.557	5.552
9.981	9.974	9.949	9.943	9.936	9.914	9.909	9.903	9.884
6.679	6.679	6.679	6.679	6.678	6.676	6.676	6.675	6.675
5.903	5.903	5.903	5.903	5.903	5.900	5.900	5.901	5.900
6.661	6.654	6.64	6.629	6.625	6.607	6.601	6.598	6.585
6.975	6.973	6.973	6.971	6.969	6.967	6.965	6.965	6.963
6.974	6.968	6.96	6.957	6.949	6.946	6.943	6.935	6.933
4.979	4.982	4.980	4.982	4.979	4.980	4.979	4.978	4.978
6.937	6.934	6.935	6.934	6.931	6.931	6.930	6.929	6.927
5.424	5.422	5.418	5.416	5.414	5.409	5.409	5.406	5.404
6.106	6.106	6.101	6.093	6.09	6.086	6.079	6.076	6.074
6.781	6.776	6.768	6.759	6.756	6.745	6.743	6.738	6.733
7.399	7.382	7.374	7.358	7.351	7.342	7.329	7.323	7.311
5.326	5.325	5.321	5.321	5.317	5.316	5.314	5.312	5.31
5.523	5.512	5.506	5.496	5.492	5.482	5.48	5.474	5.471
6.853	6.852	6.849	6.845	6.844	6.837	6.836	6.83	6.829
5.929	5.926	5.919	5.916	5.912	5.907	5.906	5.899	5.899
7.002	6.999	6.999	6.997	6.998	6.997	6.997	6.997	6.994
7.016	7.015	7.015	7.01	7.009	7.003	7.003	7.001	6.996
7.225	7.225	7.225	7.225	7.225	7.225	7.227	7.224	7.223
6.208	6.201	6.184	6.18	6.164	6.16	6.154	6.144	6.14
5.935	5.923	5.919	5.905	5.9	5.896	5.885	5.881	5.874
5.23	5.226	5.226	5.223	5.218	5.218	5.214	5.214	5.21
5.017	5.012	5.003	4.999	4.993	4.988	4.984	4.977	4.975
5.946	5.927	5.924	5.92	5.912	5.902	5.899	5.896	5.884
5.08	5.071	5.068	5.058	5.056	5.049	5.045	5.041	5.039
6.267	6.263	6.255	6.244	6.237	6.228	6.223	6.213	6.21
6.491	6.486	6.47	6.458	6.455	6.451	6.447	6.44	6.428
5.825	5.807	5.804	5.8	5.798	5.794	5.783	5.782	5.78
7.417	7.412	7.404	7.396	7.388	7.38	7.371	7.353	7.351
4.721	4.713	4.712	4.709	4.707	4.701	4.7	4.698	4.696
6.376	6.371	6.363	6.356	6.35	6.343	6.338	6.332	6.329
7.511	7.506	7.5	7.482	7.479	7.476	7.462	7.457	7.455
10.439	10.428	10.411	10.406	10.397	10.381	10.377	10.369	10.355
5.52	5.52	5.519	5.513	5.511	5.509	5.505	5.503	5.503
5.466	5.462	5.461	5.459	5.455	5.454	5.453	5.45	5.447
4.736	4.733	4.73	4.724	4.723	4.722	4.713	4.714	4.713

124	126	128	130	132	134	136	138	140
6.441	6.438	6.433	6.432	6.431	6.428	6.428	6.426	6.424
7.952	7.944	7.941	7.933	7.93	7.926	7.922	7.921	7.917
4.676	4.675	4.673	4.672	4.67	4.671	4.67	4.669	4.669
5.55	5.544	5.544	5.542	5.539	5.538	5.536	5.535	5.532
9.879	9.874	9.861	9.857	9.854	9.849	9.839	9.836	9.834
6.672	6.672	6.670	6.670	6.668	6.667	6.664	6.664	6.663
5.900	5.899	5.900	5.897	5.897	5.897	5.896	5.897	5.895
6.582	6.579	6.569	6.567	6.564	6.556	6.555	6.553	6.551
6.961	6.96	6.959	6.954	6.954	6.95	6.951	6.95	6.947
6.926	6.924	6.921	6.917	6.916	6.911	6.911	6.91	6.909
4.978	4.979	4.978	4.973	4.975	4.975	4.973	4.973	4.975
6.926	6.923	6.923	6.922	6.918	6.919	6.919	6.918	6.914
5.403	5.399	5.398	5.397	5.395	5.394	5.394	5.392	5.391
6.073	6.067	6.066	6.065	6.062	6.061	6.058	6.057	6.055
6.731	6.725	6.726	6.723	6.719	6.718	6.715	6.715	6.715
7.307	7.302	7.295	7.292	7.284	7.282	7.278	7.274	7.274
5.309	5.308	5.305	5.306	5.302	5.301	5.301	5.3	5.3
5.467	5.462	5.462	5.457	5.457	5.452	5.451	5.448	5.447
6.826	6.824	6.821	6.818	6.817	6.813	6.814	6.812	6.81
5.897	5.893	5.894	5.889	5.888	5.887	5.884	5.883	5.883
6.994	6.993	6.993	6.990	6.991	6.989	6.989	6.987	6.986
6.995	6.99	6.989	6.987	6.984	6.982	6.979	6.978	6.977
7.223	7.223	7.220	7.221	7.219	7.227	7.217	7.217	7.216
6.128	6.126	6.123	6.116	6.114	6.107	6.106	6.1	6.099
5.869	5.866	5.86	5.856	5.857	5.853	5.849	5.849	5.846
5.208	5.203	5.203	5.202	5.198	5.197	5.195	5.195	5.194
4.969	4.969	4.964	4.964	4.959	4.959	4.956	4.956	4.953
5.882	5.88	5.876	5.868	5.867	5.867	5.863	5.859	5.858
5.035	5.032	5.029	5.026	5.024	5.022	5.021	5.018	5.017
6.202	6.197	6.195	6.19	6.188	6.182	6.18	6.176	6.176
6.425	6.422	6.42	6.417	6.414	6.41	6.41	6.408	6.406
5.779	5.776	5.775	5.772	5.771	5.768	5.767	5.766	5.765
7.349	7.344	7.34	7.337	7.336	7.332	7.331	7.328	7.327
4.693	4.693	4.693	4.692	4.69	4.687	4.688	4.687	4.686
6.324	6.321	6.315	6.314	6.311	6.309	6.309	6.306	6.306
7.451	7.442	7.441	7.438	7.431	7.43	7.428	7.423	7.425
10.351	10.345	10.333	10.327	10.324	10.314	10.312	10.309	10.302
5.5	5.498	5.499	5.496	5.495	5.495	5.495	5.492	5.492
5.447	5.444	5.445	5.443	5.442	5.441	5.441	5.44	5.437
4.709	4.708	4.709	4.706	4.705	4.705	4.705	4.701	4.704

142	144	146	148	150	152	154	156	158
6.423	6.423	6.421	6.421	6.42	6.42	6.419	6.419	6.416
7.915	7.912	7.912	7.91	7.909	7.909	7.909	7.908	7.908
4.666	4.667	4.666	4.666	4.665	4.663	4.664	4.663	4.665
5.533	5.532	5.529	5.528	5.528	5.526	5.525	5.524	5.522
9.828	9.827	9.826	9.819	9.819	9.818	9.816	9.814	9.812
6.664	6.662	6.66	6.662	6.658	6.656	6.656	6.655	6.654
5.896	5.895	5.895	5.895	5.893	5.892	5.892	5.891	5.891
6.549	6.547	6.544	6.544	6.543	6.541	6.539	6.539	6.538
6.947	6.947	6.945	6.943	6.942	6.942	6.941	6.941	6.941
6.905	6.905	6.905	6.901	6.901	6.901	6.899	6.898	6.897
4.973	4.972	4.972	4.972	4.972	4.97	4.97	4.97	4.969
6.914	6.914	6.912	6.912	6.911	6.911	6.911	6.91	6.91
5.39	5.39	5.39	5.388	5.387	5.388	5.387	5.386	5.386
6.055	6.054	6.053	6.053	6.054	6.05	6.051	6.051	6.051
6.712	6.714	6.713	6.711	6.711	6.71	6.71	6.71	6.707
7.269	7.268	7.268	7.266	7.266	7.263	7.262	7.262	7.262
5.297	5.297	5.296	5.296	5.296	5.294	5.298	5.297	5.293
5.447	5.444	5.444	5.442	5.441	5.441	5.439	5.44	5.438
6.81	6.809	6.809	6.807	6.805	6.805	6.804	6.804	6.804
5.883	5.883	5.882	5.879	5.88	5.878	5.878	5.878	5.878
6.986	6.983	6.983	6.981	6.979	6.979	6.978	6.978	6.978
6.975	6.974	6.972	6.971	6.97	6.967	6.966	6.966	6.964
7.213	7.213	7.213	7.212	7.213	7.211	7.208	7.208	7.206
6.095	6.092	6.092	6.088	6.088	6.086	6.082	6.082	6.079
5.844	5.841	5.841	5.84	5.838	5.838	5.838	5.837	5.836
5.191	5.19	5.189	5.189	5.187	5.185	5.186	5.185	5.186
4.953	4.953	4.951	4.951	4.949	4.951	4.949	4.948	4.949
5.856	5.856	5.854	5.854	5.853	5.85	5.85	5.85	5.848
5.015	5.016	5.014	5.015	5.012	5.012	5.012	5.011	5.011
6.173	6.172	6.172	6.168	6.168	6.167	6.164	6.164	6.161
6.403	6.399	6.399	6.401	6.4	6.399	6.398	6.398	6.398
5.764	5.762	5.762	5.762	5.762	5.762	5.762	5.76	5.762
7.324	7.323	7.323	7.323	7.322	7.321	7.321	7.321	7.32
4.686	4.687	4.686	4.686	4.685	4.685	4.684	4.685	4.685
6.306	6.303	6.303	6.303	6.303	6.303	6.302	6.302	6.302
7.424	7.423	7.42	7.42	7.419	7.416	7.418	7.417	7.416
10.301	10.299	10.294	10.294	10.292	10.288	10.288	10.287	10.287
5.493	5.49	5.491	5.491	5.488	5.489	5.489	5.488	5.487
5.437	5.437	5.435	5.436	5.435	5.435	5.433	5.435	5.434
4.702	4.702	4.701	4.701	4.701	4.7	4.7	4.699	4.698

160	162	164	166	168	170	172	174	176
6.417	6.416	6.416	6.416	6.416	6.416	6.414	6.415	6.413
7.906	7.905	7.906	7.905	7.904	7.902	7.903	7.902	7.901
4.662	4.663	4.663	4.663	4.661	4.662	4.661	4.661	4.661
5.522	5.52	5.52	5.517	5.518	5.517	5.516	5.516	5.514
9.81	9.808	9.807	9.806	9.803	9.802	9.803	9.8	9.8
6.654	6.653	6.651	6.65	6.648	6.646	6.646	6.646	6.644
5.892	5.891	5.889	5.889	5.888	5.888	5.887	5.886	5.885
6.535	6.535	6.535	6.533	6.532	6.532	6.531	6.529	6.528
6.939	6.938	6.937	6.937	6.936	6.935	6.935	6.933	6.932
6.897	6.895	6.894	6.894	6.892	6.891	6.892	6.89	6.89
4.969	4.969	4.969	4.967	4.967	4.968	4.967	4.965	4.965
6.907	6.907	6.908	6.906	6.908	6.907	6.907	6.907	6.905
5.386	5.386	5.384	5.386	5.384	5.383	5.384	5.383	5.382
6.048	6.049	6.047	6.046	6.046	6.046	6.045	6.045	6.045
6.707	6.707	6.707	6.705	6.704	6.705	6.702	6.702	6.702
7.259	7.26	7.258	7.258	7.258	7.256	7.256	7.256	7.255
5.294	5.294	5.293	5.294	5.294	5.293	5.291	5.292	5.292
5.438	5.437	5.436	5.436	5.435	5.436	5.434	5.434	5.432
6.802	6.801	6.801	6.801	6.8	6.8	6.798	6.797	6.796
5.876	5.876	5.876	5.876	5.874	5.874	5.874	5.874	5.874
6.976	6.975	6.975	6.973	6.973	6.971	6.971	6.97	6.969
6.964	6.964	6.963	6.961	6.96	6.961	6.959	6.959	6.958
7.205	7.206	7.204	7.204	7.202	7.202	7.201	7.2	7.199
6.079	6.078	6.075	6.075	6.075	6.073	6.07	6.07	6.068
5.834	5.833	5.833	5.832	5.832	5.83	5.832	5.83	5.83
5.185	5.184	5.183	5.182	5.18	5.181	5.18	5.18	5.179
4.947	4.948	4.947	4.946	4.945	4.944	4.945	4.943	4.944
5.846	5.846	5.846	5.844	5.844	5.844	5.842	5.842	5.84
5.009	5.008	5.007	5.006	5.005	5.007	5.005	5.004	5.004
6.163	6.16	6.159	6.159	6.158	6.157	6.159	6.157	6.156
6.397	6.395	6.396	6.395	6.395	6.395	6.395	6.394	6.394
5.762	5.76	5.762	5.759	5.759	5.76	5.76	5.76	5.759
7.321	7.319	7.319	7.319	7.317	7.318	7.317	7.316	7.315
4.684	4.684	4.683	4.684	4.684	4.684	4.682	4.682	4.682
6.301	6.301	6.301	6.299	6.299	6.299	6.299	6.298	6.298
7.416	7.415	7.414	7.414	7.415	7.414	7.413	7.41	7.41
10.284	10.284	10.284	10.283	10.282	10.283	10.28	10.281	10.28
5.487	5.487	5.488	5.487	5.487	5.485	5.485	5.485	5.484
5.434	5.435	5.433	5.433	5.433	5.432	5.433	5.431	5.431
4.698	4.698	4.697	4.697	4.697	4.697	4.696	4.696	4.694

178	180	182	184	186	188	190	192	194
6.412	6.412	6.411	6.409	6.408	6.408	6.407	6.406	6.404
7.9	7.9	7.9	7.898	7.898	7.898	7.897	7.896	7.895
4.661	4.66	4.66	4.659	4.659	4.658	4.657	4.656	4.655
5.514	5.513	5.511	5.511	5.509	5.509	5.507	5.507	5.505
9.799	9.798	9.796	9.795	9.792	9.791	9.791	9.79	9.788
6.643	6.642	6.642	6.642	6.642	6.64	6.637	6.636	6.636
5.885	5.885	5.885	5.885	5.885	5.884	5.883	5.881	5.88
6.527	6.525	6.525	6.524	6.523	6.521	6.521	6.518	6.517
6.931	6.93	6.93	6.929	6.927	6.926	6.926	6.925	6.923
6.889	6.889	6.887	6.885	6.886	6.883	6.882	6.882	6.881
4.968	4.967	4.965	4.965	4.965	4.964	4.964	4.963	4.962
6.905	6.905	6.904	6.903	6.903	6.903	6.903	6.902	6.902
5.383	5.383	5.383	5.382	5.38	5.38	5.38	5.38	5.38
6.043	6.042	6.043	6.042	6.04	6.04	6.04	6.039	6.039
6.7	6.701	6.7	6.699	6.698	6.698	6.698	6.696	6.695
7.252	7.252	7.252	7.251	7.25	7.25	7.25	7.248	7.248
5.29	5.29	5.289	5.289	5.289	5.287	5.287	5.288	5.287
5.43	5.43	5.43	5.429	5.428	5.426	5.425	5.424	5.425
6.797	6.798	6.797	6.796	6.796	6.793	6.793	6.793	6.792
5.872	5.871	5.87	5.87	5.869	5.868	5.868	5.868	5.867
6.967	6.966	6.965	6.965	6.963	6.962	6.96	6.958	6.957
6.958	6.956	6.956	6.955	6.952	6.951	6.951	6.949	6.948
7.197	7.197	7.196	7.194	7.193	7.192	7.19	7.188	7.188
6.067	6.064	6.063	6.061	6.06	6.06	6.056	6.055	6.054
5.829	5.826	5.825	5.825	5.825	5.824	5.823	5.821	5.819
5.179	5.178	5.176	5.175	5.174	5.173	5.172	5.171	5.168
4.944	4.943	4.941	4.94	4.94	4.939	4.939	4.937	4.937
5.84	5.838	5.838	5.836	5.835	5.834	5.832	5.831	5.83
5.004	5.003	5.001	5.001	4.999	5	4.997	4.996	4.995
6.155	6.153	6.153	6.153	6.152	6.151	6.149	6.147	6.147
6.393	6.394	6.394	6.393	6.393	6.392	6.393	6.391	6.391
5.759	5.758	5.756	5.756	5.756	5.756	5.756	5.756	5.755
7.315	7.315	7.315	7.313	7.313	7.312	7.311	7.312	7.309
4.681	4.681	4.681	4.681	4.681	4.681	4.681	4.68	4.678
6.298	6.297	6.297	6.297	6.294	6.294	6.294	6.294	6.294
7.411	7.41	7.41	7.408	7.408	7.408	7.408	7.408	7.407
10.279	10.279	10.278	10.278	10.278	10.277	10.276	10.276	10.276
5.484	5.484	5.483	5.483	5.483	5.481	5.48	5.479	5.479
5.43	5.431	5.43	5.43	5.429	5.429	5.427	5.427	5.426
4.693	4.694	4.694	4.693	4.693	4.692	4.692	4.692	4.69

196	198	200	202	204	206	208	210	212
6.405	6.403	6.403	6.401	6.399	6.397	6.396	6.396	6.393
7.896	7.894	7.893	7.891	7.89	7.889	7.889	7.888	7.885
4.654	4.654	4.652	4.651	4.649	4.649	4.648	4.646	4.645
5.505	5.504	5.503	5.502	5.5	5.498	5.496	5.494	5.493
9.786	9.786	9.783	9.78	9.779	9.777	9.774	9.772	9.77
6.634	6.634	6.632	6.631	6.63	6.627	6.626	6.623	6.621
5.879	5.877	5.877	5.877	5.875	5.873	5.871	5.871	5.868
6.516	6.516	6.514	6.511	6.511	6.509	6.508	6.505	6.504
6.922	6.921	6.921	6.919	6.918	6.917	6.915	6.914	6.911
6.88	6.878	6.878	6.877	6.875	6.874	6.872	6.87	6.869
4.961	4.961	4.96	4.96	4.959	4.959	4.959	4.957	4.957
6.902	6.902	6.9	6.898	6.899	6.897	6.897	6.895	6.895
5.379	5.378	5.378	5.378	5.377	5.376	5.374	5.374	5.374
6.038	6.038	6.037	6.038	6.036	6.036	6.034	6.034	6.032
6.695	6.695	6.692	6.692	6.691	6.69	6.689	6.686	6.683
7.248	7.247	7.247	7.244	7.244	7.243	7.243	7.242	7.242
5.286	5.286	5.285	5.284	5.283	5.281	5.281	5.281	5.28
5.424	5.422	5.421	5.421	5.419	5.419	5.417	5.416	5.414
6.792	6.792	6.791	6.789	6.789	6.788	6.787	6.785	6.784
5.867	5.867	5.866	5.866	5.864	5.864	5.863	5.862	5.86
6.955	6.951	6.95	6.947	6.946	6.942	6.941	6.935	6.933
6.946	6.944	6.942	6.941	6.939	6.937	6.935	6.933	6.931
7.186	7.183	7.18	7.178	7.177	7.174	7.169	7.166	7.164
6.051	6.048	6.045	6.044	6.041	6.039	6.035	6.032	6.029
5.818	5.818	5.816	5.814	5.813	5.813	5.809	5.808	5.805
5.167	5.166	5.164	5.162	5.16	5.159	5.156	5.153	5.15
4.936	4.934	4.933	4.931	4.929	4.928	4.925	4.923	4.922
5.827	5.824	5.824	5.82	5.818	5.816	5.815	5.81	5.808
4.995	4.992	4.989	4.988	4.986	4.984	4.983	4.98	4.977
6.145	6.144	6.143	6.141	6.139	6.136	6.135	6.132	6.129
6.391	6.39	6.389	6.389	6.387	6.387	6.387	6.386	6.385
5.754	5.754	5.751	5.751	5.75	5.75	5.748	5.748	5.748
7.308	7.308	7.305	7.304	7.304	7.302	7.302	7.301	7.299
4.678	4.677	4.675	4.674	4.675	4.673	4.673	4.672	4.672
6.293	6.293	6.292	6.29	6.29	6.289	6.287	6.287	6.287
7.405	7.406	7.405	7.403	7.404	7.401	7.402	7.4	7.399
10.275	10.272	10.271	10.271	10.27	10.27	10.267	10.267	10.266
5.479	5.479	5.477	5.477	5.475	5.473	5.474	5.473	5.473
5.427	5.426	5.423	5.423	5.422	5.422	5.422	5.421	5.419
4.69	4.689	4.689	4.689	4.688	4.688	4.686	4.685	4.685

214	216	218	220	222	224	226	228	230
6.391	6.39	6.388	6.385	6.381	6.379	6.376	6.372	6.369
7.883	7.883	7.882	7.88	7.877	7.876	7.873	7.87	7.868
4.642	4.642	4.639	4.638	4.635	4.632	4.629	4.628	4.626
5.492	5.489	5.486	5.484	5.482	5.479	5.476	5.473	5.47
9.767	9.764	9.761	9.758	9.754	9.751	9.747	9.742	9.738
6.619	6.618	6.615	6.611	6.608	6.605	6.602	6.599	6.595
5.867	5.864	5.863	5.861	5.859	5.856	5.853	5.851	5.848
6.501	6.499	6.496	6.495	6.492	6.489	6.486	6.483	6.48
6.91	6.907	6.906	6.905	6.902	6.898	6.894	6.892	6.888
6.867	6.864	6.863	6.86	6.858	6.855	6.852	6.849	6.845
4.955	4.955	4.953	4.952	4.952	4.949	4.95	4.948	4.947
6.895	6.893	6.892	6.891	6.89	6.887	6.886	6.883	6.88
5.373	5.372	5.37	5.37	5.367	5.366	5.364	5.362	5.36
6.032	6.031	6.028	6.028	6.026	6.023	6.023	6.02	6.018
6.683	6.68	6.678	6.677	6.674	6.672	6.668	6.664	6.662
7.24	7.238	7.237	7.235	7.233	7.231	7.23	7.227	7.224
5.28	5.278	5.277	5.276	5.276	5.273	5.272	5.27	5.268
5.411	5.409	5.408	5.406	5.404	5.401	5.4	5.396	5.393
6.782	6.781	6.78	6.777	6.777	6.774	6.774	6.771	6.769
5.859	5.859	5.858	5.856	5.855	5.853	5.851	5.851	5.848
6.929	6.925	6.92	6.916	6.91	6.903	6.9	6.892	6.886
6.927	6.924	6.922	6.919	6.914	6.911	6.906	6.902	6.897
7.159	7.154	7.15	7.145	7.139	7.134	7.128	7.121	7.114
6.025	6.022	6.017	6.014	6.008	6.003	5.997	5.991	5.985
5.803	5.8	5.798	5.794	5.79	5.788	5.785	5.781	5.777
5.146	5.144	5.14	5.135	5.131	5.126	5.122	5.118	5.113
4.918	4.916	4.913	4.909	4.906	4.903	4.896	4.893	4.889
5.804	5.8	5.798	5.792	5.788	5.783	5.777	5.772	5.767
4.975	4.971	4.968	4.964	4.959	4.956	4.952	4.946	4.942
6.125	6.122	6.12	6.117	6.113	6.108	6.104	6.1	6.094
6.385	6.383	6.382	6.381	6.379	6.377	6.374	6.371	6.37
5.746	5.746	5.744	5.743	5.742	5.739	5.738	5.737	5.735
7.298	7.296	7.293	7.292	7.289	7.288	7.285	7.283	7.28
4.67	4.67	4.669	4.668	4.666	4.666	4.664	4.662	4.661
6.286	6.283	6.282	6.282	6.279	6.278	6.275	6.274	6.271
7.396	7.395	7.394	7.392	7.392	7.39	7.388	7.385	7.383
10.264	10.263	10.261	10.262	10.259	10.257	10.255	10.253	10.251
5.471	5.469	5.469	5.467	5.465	5.465	5.464	5.461	5.46
5.419	5.417	5.417	5.414	5.413	5.412	5.411	5.409	5.407
4.684	4.682	4.682	4.68	4.68	4.678	4.678	4.676	4.673

232	234	236	238	240	242	244	246	248
6.367	6.363	6.359	6.356	6.352	6.346	6.342	6.337	6.332
7.865	7.862	7.859	7.856	7.852	7.848	7.843	7.838	7.834
4.622	4.619	4.615	4.611	4.608	4.605	4.601	4.595	4.59
5.466	5.462	5.46	5.454	5.45	5.445	5.441	5.434	5.429
9.732	9.728	9.722	9.715	9.71	9.703	9.695	9.685	9.677
6.591	6.587	6.582	6.578	6.572	6.565	6.56	6.553	6.546
5.845	5.841	5.837	5.833	5.829	5.826	5.82	5.814	5.809
6.477	6.473	6.47	6.465	6.461	6.455	6.451	6.446	6.44
6.885	6.882	6.878	6.873	6.869	6.863	6.859	6.853	6.846
6.842	6.838	6.834	6.829	6.824	6.82	6.814	6.809	6.8
4.944	4.942	4.939	4.937	4.935	4.933	4.929	4.927	4.923
6.878	6.876	6.874	6.871	6.867	6.865	6.861	6.858	6.855
5.358	5.356	5.354	5.351	5.349	5.346	5.342	5.338	5.335
6.017	6.014	6.012	6.008	6.006	6.003	6	5.997	5.992
6.657	6.653	6.649	6.647	6.641	6.636	6.63	6.624	6.619
7.222	7.22	7.217	7.214	7.212	7.207	7.204	7.199	7.196
5.267	5.264	5.262	5.261	5.258	5.256	5.253	5.25	5.247
5.39	5.386	5.384	5.381	5.378	5.373	5.369	5.364	5.358
6.766	6.764	6.761	6.758	6.755	6.752	6.747	6.743	6.738
5.846	5.844	5.841	5.839	5.836	5.835	5.83	5.828	5.823
6.879	6.871	6.863	6.855	6.845	6.836	6.825	6.813	6.8
6.892	6.885	6.878	6.873	6.865	6.857	6.849	6.841	6.832
7.106	7.1	7.093	7.084	7.075	7.064	7.053	7.042	7.029
5.978	5.97	5.963	5.955	5.944	5.936	5.925	5.914	5.902
5.771	5.768	5.761	5.755	5.749	5.742	5.734	5.726	5.718
5.107	5.1	5.093	5.086	5.079	5.07	5.063	5.054	5.043
4.884	4.879	4.873	4.866	4.86	4.853	4.844	4.835	4.825
5.759	5.752	5.745	5.735	5.727	5.718	5.708	5.697	5.687
4.936	4.929	4.924	4.915	4.907	4.899	4.889	4.879	4.868
6.089	6.084	6.076	6.072	6.063	6.055	6.047	6.039	6.028
6.367	6.366	6.362	6.359	6.356	6.354	6.35	6.345	6.341
5.733	5.732	5.729	5.727	5.724	5.723	5.719	5.715	5.712
7.277	7.273	7.269	7.265	7.261	7.258	7.253	7.247	7.241
4.66	4.658	4.656	4.653	4.653	4.648	4.646	4.643	4.641
6.27	6.269	6.265	6.264	6.262	6.258	6.254	6.25	6.247
7.381	7.379	7.376	7.372	7.37	7.365	7.362	7.357	7.351
10.248	10.246	10.244	10.239	10.235	10.232	10.227	10.221	10.216
5.457	5.455	5.453	5.45	5.448	5.444	5.441	5.438	5.433
5.406	5.403	5.401	5.397	5.395	5.393	5.389	5.385	5.382
4.67	4.67	4.666	4.665	4.662	4.66	4.656	4.653	4.649

250	252	254	256	258	260	262	264	266
6.326	6.321	6.313	6.307	6.299	6.292	6.282	6.274	6.264
7.829	7.822	7.817	7.81	7.801	7.794	7.784	7.773	7.762
4.585	4.579	4.575	4.567	4.562	4.554	4.546	4.538	4.529
5.422	5.416	5.408	5.4	5.392	5.383	5.373	5.363	5.354
9.667	9.656	9.644	9.631	9.62	9.607	9.591	9.574	9.558
6.54	6.531	6.523	6.513	6.503	6.493	6.482	6.47	6.456
5.803	5.799	5.791	5.784	5.776	5.768	5.758	5.749	5.74
6.433	6.425	6.418	6.41	6.401	6.394	6.384	6.374	6.362
6.841	6.834	6.826	6.817	6.807	6.796	6.787	6.775	6.764
6.793	6.786	6.777	6.766	6.757	6.747	6.736	6.724	6.711
4.921	4.917	4.912	4.909	4.904	4.899	4.894	4.888	4.882
6.85	6.844	6.838	6.833	6.828	6.822	6.815	6.808	6.8
5.332	5.328	5.322	5.319	5.315	5.308	5.303	5.296	5.289
5.988	5.983	5.978	5.973	5.967	5.96	5.955	5.947	5.94
6.612	6.603	6.598	6.591	6.583	6.575	6.566	6.557	6.547
7.191	7.185	7.18	7.175	7.168	7.161	7.153	7.145	7.137
5.244	5.241	5.236	5.229	5.225	5.221	5.213	5.209	5.202
5.353	5.347	5.34	5.332	5.325	5.317	5.309	5.301	5.291
6.734	6.729	6.723	6.717	6.712	6.704	6.698	6.688	6.68
5.82	5.816	5.812	5.807	5.802	5.796	5.791	5.784	5.775
6.786	6.771	6.757	6.739	6.719	6.702	6.682	6.661	6.639
6.821	6.809	6.796	6.782	6.769	6.754	6.736	6.721	6.701
7.016	7	6.983	6.967	6.948	6.932	6.911	6.89	6.868
5.889	5.877	5.862	5.847	5.831	5.814	5.795	5.777	5.756
5.709	5.7	5.689	5.676	5.664	5.652	5.638	5.623	5.609
5.032	5.02	5.008	4.996	4.982	4.969	4.954	4.939	4.921
4.815	4.803	4.793	4.781	4.767	4.755	4.74	4.725	4.711
5.672	5.66	5.644	5.628	5.612	5.597	5.579	5.561	5.541
4.858	4.846	4.834	4.82	4.806	4.791	4.773	4.759	4.743
6.016	6.004	5.993	5.979	5.966	5.95	5.934	5.917	5.898
6.337	6.333	6.326	6.32	6.314	6.308	6.301	6.293	6.285
5.708	5.704	5.701	5.697	5.691	5.685	5.679	5.672	5.665
7.235	7.228	7.22	7.213	7.203	7.193	7.183	7.173	7.161
4.638	4.635	4.631	4.627	4.624	4.618	4.614	4.608	4.602
6.243	6.239	6.234	6.228	6.223	6.217	6.211	6.204	6.197
7.346	7.341	7.335	7.329	7.324	7.315	7.308	7.299	7.291
10.21	10.204	10.198	10.191	10.183	10.175	10.167	10.157	10.15
5.431	5.426	5.421	5.417	5.412	5.405	5.399	5.392	5.385
5.379	5.374	5.369	5.365	5.36	5.354	5.347	5.342	5.334
4.646	4.643	4.639	4.634	4.629	4.625	4.62	4.614	4.606

268	270	272	274	276	278	280	282	284
6.254	6.244	6.23	6.217	6.203	6.187	6.173	6.156	6.139
7.751	7.74	7.726	7.711	7.696	7.679	7.663	7.646	7.628
4.519	4.509	4.496	4.485	4.475	4.462	4.449	4.436	4.421
5.343	5.331	5.32	5.306	5.292	5.279	5.262	5.246	5.229
9.539	9.519	9.498	9.476	9.452	9.428	9.403	9.375	9.348
6.442	6.429	6.414	6.397	6.38	6.362	6.344	6.326	6.306
5.729	5.717	5.704	5.692	5.678	5.665	5.649	5.632	5.615
6.351	6.338	6.324	6.311	6.294	6.28	6.264	6.246	6.228
6.75	6.736	6.72	6.706	6.69	6.673	6.655	6.637	6.617
6.697	6.683	6.668	6.652	6.635	6.618	6.6	6.579	6.559
4.876	4.869	4.859	4.85	4.84	4.831	4.82	4.809	4.798
6.79	6.781	6.772	6.761	6.749	6.737	6.724	6.709	6.694
5.28	5.273	5.264	5.254	5.244	5.233	5.221	5.21	5.197
5.931	5.921	5.912	5.901	5.89	5.879	5.866	5.853	5.84
6.537	6.526	6.515	6.503	6.491	6.478	6.463	6.449	6.433
7.129	7.119	7.107	7.096	7.082	7.07	7.056	7.04	7.023
5.194	5.186	5.177	5.168	5.158	5.147	5.137	5.125	5.113
5.28	5.27	5.258	5.247	5.233	5.218	5.203	5.19	5.172
6.669	6.658	6.647	6.638	6.624	6.61	6.595	6.582	6.564
5.767	5.76	5.752	5.742	5.734	5.723	5.712	5.698	5.687
6.618	6.592	6.568	6.543	6.518	6.491	6.462	6.435	6.404
6.682	6.663	6.64	6.62	6.596	6.572	6.549	6.522	6.496
6.846	6.825	6.8	6.775	6.752	6.722	6.696	6.666	6.639
5.734	5.714	5.691	5.668	5.646	5.621	5.596	5.57	5.542
5.593	5.578	5.561	5.542	5.523	5.522	5.503	5.482	5.46
4.904	4.886	4.869	4.849	4.828	4.808	4.788	4.766	4.744
4.694	4.674	4.656	4.638	4.62	4.6	4.582	4.56	4.539
5.522	5.503	5.482	5.46	5.437	5.412	5.39	5.365	5.341
4.725	4.707	4.688	4.669	4.651	4.63	4.61	4.588	4.566
5.881	5.862	5.842	5.82	5.801	5.78	5.755	5.73	5.705
6.277	6.268	6.258	6.247	6.233	6.222	6.209	6.194	6.181
5.659	5.651	5.641	5.632	5.624	5.612	5.601	5.588	5.575
7.149	7.135	7.121	7.105	7.09	7.075	7.056	7.036	7.017
4.595	4.59	4.583	4.576	4.568	4.559	4.549	4.54	4.528
6.19	6.182	6.171	6.162	6.151	6.138	6.126	6.112	6.099
7.28	7.271	7.26	7.247	7.235	7.221	7.205	7.189	7.173
10.138	10.127	10.114	10.101	10.087	10.07	10.055	10.038	10.019
5.376	5.369	5.36	5.35	5.34	5.327	5.316	5.303	5.29
5.328	5.321	5.311	5.301	5.293	5.28	5.271	5.258	5.245
4.6	4.594	4.586	4.578	4.568	4.558	4.549	4.536	4.525

286	288	290	292	294	296	298	300	302
6.121	6.104	6.085	6.064	6.043	6.019	5.994	5.971	5.944
7.608	7.588	7.566	7.543	7.518	7.493	7.466	7.437	7.407
4.408	4.391	4.376	4.36	4.342	4.325	4.306	4.286	4.267
5.211	5.193	5.174	5.153	5.132	5.109	5.085	5.061	5.034
9.318	9.287	9.255	9.222	9.187	9.15	9.109	9.067	9.024
6.284	6.261	6.235	6.209	6.183	6.154	6.127	6.096	6.064
5.599	5.579	5.56	5.539	5.518	5.494	5.469	5.442	5.412
6.207	6.187	6.167	6.144	6.12	6.094	6.066	6.037	6.005
6.596	6.574	6.552	6.529	6.503	6.477	6.449	6.42	6.391
6.538	6.514	6.49	6.467	6.441	6.416	6.387	6.358	6.325
4.787	4.774	4.759	4.744	4.729	4.711	4.694	4.673	4.654
6.677	6.66	6.641	6.623	6.603	6.58	6.557	6.532	6.505
5.182	5.169	5.153	5.137	5.119	5.1	5.082	5.06	5.038
5.826	5.809	5.793	5.776	5.756	5.737	5.717	5.695	5.671
6.415	6.396	6.374	6.353	6.328	6.303	6.274	6.242	6.209
7.007	6.989	6.969	6.949	6.928	6.904	6.879	6.853	6.826
5.1	5.085	5.07	5.052	5.035	5.017	4.995	4.972	4.949
5.155	5.138	5.118	5.101	5.082	5.061	5.039	5.017	4.992
6.547	6.53	6.51	6.492	6.469	6.447	6.421	6.396	6.367
5.672	5.659	5.644	5.626	5.61	5.589	5.568	5.546	5.524
6.373	6.342	6.311	6.278	6.242	6.208	6.171	6.134	6.095
6.469	6.44	6.411	6.381	6.35	6.317	6.284	6.252	6.215
6.611	6.581	6.547	6.513	6.48	6.448	6.413	6.375	6.337
5.516	5.487	5.457	5.425	5.396	5.364	5.331	5.299	5.266
5.439	5.415	5.388	5.364	5.342	5.317	5.289	5.259	5.216
4.721	4.697	4.673	4.647	4.623	4.596	4.571	4.543	4.516
4.518	4.494	4.469	4.447	4.423	4.397	4.373	4.346	4.32
5.316	5.29	5.262	5.233	5.204	5.175	5.143	5.111	5.082
4.544	4.521	4.496	4.473	4.449	4.423	4.394	4.365	4.339
5.68	5.653	5.629	5.602	5.574	5.544	5.513	5.481	5.45
6.165	6.146	6.13	6.11	6.094	6.072	6.052	6.029	6.005
5.561	5.545	5.531	5.515	5.498	5.48	5.46	5.44	5.418
6.995	6.972	6.95	6.926	6.903	6.877	6.849	6.82	6.789
4.516	4.505	4.49	4.477	4.463	4.448	4.433	4.416	4.4
6.085	6.07	6.053	6.037	6.018	5.998	5.979	5.957	5.934
7.156	7.139	7.118	7.098	7.078	7.055	7.031	7.008	6.982
9.999	9.979	9.957	9.936	9.911	9.884	9.857	9.829	9.798
5.275	5.26	5.244	5.227	5.209	5.19	5.17	5.15	5.128
5.233	5.22	5.206	5.192	5.175	5.157	5.141	5.122	5.101
4.512	4.5	4.487	4.473	4.457	4.442	4.425	4.407	4.388

304	306	308	310	312	314	316	318	320
5.918	5.888	5.861	5.831	5.801	5.766	5.735	5.7	5.663
7.377	7.344	7.312	7.274	7.236	7.199	7.16	7.121	7.079
4.247	4.225	4.202	4.179	4.154	4.129	4.103	4.078	4.052
5.006	4.975	4.944	4.911	4.877	4.839	4.803	4.766	4.727
8.977	8.929	8.878	8.826	8.769	8.714	8.654	8.593	8.53
6.028	5.991	5.952	5.911	5.873	5.829	5.786	5.738	5.692
5.381	5.35	5.319	5.284	5.249	5.211	5.174	5.133	5.093
5.973	5.939	5.904	5.866	5.828	5.786	5.744	5.701	5.657
6.357	6.321	6.286	6.251	6.214	6.175	6.135	6.094	6.054
6.292	6.257	6.222	6.185	6.149	6.11	6.068	6.025	5.982
4.634	4.611	4.589	4.565	4.536	4.511	4.484	4.457	4.425
6.478	6.449	6.419	6.385	6.353	6.317	6.28	6.244	6.204
5.014	4.987	4.963	4.935	4.907	4.877	4.847	4.817	4.782
5.647	5.622	5.595	5.568	5.54	5.51	5.476	5.442	5.41
6.174	6.138	6.1	6.058	6.011	5.963	5.911	5.86	5.807
6.798	6.767	6.735	6.7	6.665	6.628	6.591	6.551	6.507
4.926	4.902	4.875	4.848	4.818	4.787	4.754	4.721	4.688
4.969	4.944	4.918	4.891	4.863	4.835	4.806	4.774	4.742
6.337	6.307	6.274	6.239	6.202	6.166	6.128	6.09	6.049
5.5	5.473	5.443	5.415	5.383	5.352	5.318	5.284	5.247
6.057	6.018	5.979	5.939	5.897	5.855	5.811	5.768	5.722
6.181	6.142	6.106	6.067	6.028	5.989	5.947	5.904	5.861
6.301	6.26	6.221	6.178	6.136	6.091	6.049	6	5.954
5.23	5.195	5.16	5.121	5.084	5.045	5.009	4.97	4.932
5.182	5.151	5.121	5.088	5.055	5.023	4.986	4.952	4.917
4.489	4.46	4.43	4.399	4.367	4.337	4.304	4.27	4.237
4.292	4.265	4.237	4.206	4.176	4.146	4.115	4.085	4.053
5.049	5.016	4.983	4.95	4.915	4.882	4.847	4.809	4.77
4.312	4.283	4.254	4.225	4.196	4.164	4.133	4.102	4.069
5.417	5.384	5.348	5.315	5.279	5.241	5.205	5.167	5.127
5.982	5.954	5.929	5.9	5.872	5.843	5.812	5.779	5.744
5.396	5.372	5.348	5.326	5.302	5.274	5.246	5.217	5.188
6.758	6.726	6.696	6.66	6.621	6.581	6.541	6.5	6.462
4.382	4.363	4.343	4.321	4.3	4.279	4.256	4.232	4.206
5.911	5.888	5.862	5.835	5.807	5.778	5.746	5.713	5.681
6.954	6.924	6.893	6.861	6.825	6.792	6.756	6.718	6.679
9.765	9.731	9.696	9.659	9.618	9.574	9.53	9.485	9.438
5.106	5.082	5.058	5.031	5.007	4.978	4.949	4.919	4.888
5.08	5.059	5.036	5.011	4.986	4.96	4.933	4.905	4.875
4.369	4.35	4.33	4.309	4.285	4.264	4.241	4.215	4.189

322	324	326	328	330	332	334	336	338
5.624	5.584	5.544	5.499	5.452	5.407	5.357	5.309	5.26
7.033	6.984	6.933	6.881	6.824	6.77	6.713	6.655	6.595
4.023	3.993	3.963	3.932	3.901	3.867	3.832	3.797	3.762
4.686	4.646	4.603	4.558	4.512	4.468	4.421	4.373	4.326
8.463	8.396	8.326	8.253	8.179	8.1	8.022	7.944	7.861
5.642	5.591	5.537	5.484	5.429	5.375	5.32	5.265	5.208
5.048	5.003	4.958	4.912	4.864	4.819	4.771	4.722	4.673
5.609	5.561	5.51	5.457	5.406	5.353	5.297	5.242	5.188
6.007	5.958	5.911	5.865	5.817	5.763	5.713	5.66	5.603
5.938	5.891	5.844	5.794	5.744	5.689	5.637	5.583	5.529
4.394	4.362	4.328	4.294	4.26	4.216	4.176	4.135	4.099
6.16	6.116	6.071	6.023	5.974	5.922	5.871	5.818	5.762
4.748	4.712	4.673	4.635	4.596	4.556	4.514	4.472	4.429
5.374	5.335	5.294	5.252	5.21	5.168	5.123	5.077	5.03
5.753	5.696	5.638	5.579	5.52	5.46	5.398	5.338	5.272
6.463	6.418	6.372	6.32	6.267	6.213	6.158	6.1	6.042
4.649	4.612	4.572	4.533	4.492	4.452	4.409	4.366	4.321
4.709	4.672	4.636	4.599	4.561	4.518	4.477	4.434	4.39
6.006	5.961	5.914	5.866	5.817	5.767	5.714	5.659	5.606
5.209	5.169	5.127	5.082	5.041	4.995	4.95	4.903	4.853
5.676	5.629	5.581	5.535	5.487	5.438	5.389	5.338	5.287
5.814	5.769	5.721	5.673	5.627	5.579	5.529	5.479	5.428
5.905	5.853	5.804	5.755	5.704	5.653	5.598	5.548	5.493
4.889	4.847	4.805	4.76	4.719	4.675	4.634	4.592	4.545
4.878	4.84	4.799	4.758	4.718	4.678	4.635	4.594	4.552
4.204	4.171	4.136	4.102	4.065	4.031	3.993	3.956	3.918
4.02	3.985	3.953	3.923	3.886	3.854	3.817	3.782	3.746
4.733	4.691	4.652	4.615	4.575	4.534	4.494	4.453	4.411
4.035	4.001	3.965	3.93	3.895	3.859	3.824	3.786	3.749
5.088	5.047	5.004	4.962	4.919	4.878	4.835	4.792	4.747
5.708	5.668	5.627	5.586	5.542	5.499	5.454	5.408	5.359
5.155	5.122	5.086	5.05	5.013	4.974	4.935	4.894	4.85
6.418	6.375	6.33	6.282	6.229	6.178	6.123	6.065	6.001
4.18	4.152	4.122	4.093	4.062	4.03	3.997	3.963	3.927
5.646	5.609	5.571	5.528	5.486	5.442	5.397	5.348	5.3
6.637	6.591	6.544	6.495	6.445	6.393	6.338	6.281	6.22
9.383	9.328	9.269	9.207	9.141	9.073	9.004	8.932	8.86
4.857	4.822	4.785	4.748	4.71	4.671	4.628	4.584	4.54
4.843	4.81	4.775	4.74	4.703	4.666	4.625	4.582	4.536
4.161	4.134	4.105	4.075	4.042	4.008	3.97	3.933	3.896

340	342	344	346	348	350	352	354	356
5.207	5.153	5.098	5.042	4.982	4.919	4.857	4.793	4.727
6.534	6.472	6.405	6.333	6.261	6.185	6.11	6.031	5.953
3.724	3.686	3.649	3.606	3.566	3.522	3.475	3.432	3.387
4.28	4.233	4.185	4.136	4.085	4.036	3.982	3.929	3.878
7.778	7.696	7.609	7.527	7.442	7.351	7.264	7.175	7.087
5.149	5.089	5.028	4.969	4.911	4.847	4.787	4.724	4.663
4.621	4.571	4.519	4.468	4.416	4.361	4.306	4.252	4.198
5.131	5.074	5.013	4.953	4.892	4.826	4.765	4.699	4.637
5.546	5.49	5.431	5.373	5.313	5.252	5.191	5.131	5.068
5.472	5.417	5.359	5.3	5.24	5.174	5.113	5.048	4.984
4.062	4.023	3.981	3.937	3.893	3.845	3.797	3.74	3.686
5.705	5.644	5.581	5.519	5.454	5.388	5.32	5.25	5.179
4.382	4.335	4.287	4.238	4.186	4.136	4.083	4.029	3.973
4.98	4.93	4.878	4.823	4.769	4.712	4.655	4.594	4.537
5.206	5.142	5.077	5.013	4.947	4.879	4.807	4.736	4.663
5.985	5.922	5.86	5.794	5.727	5.662	5.59	5.518	5.445
4.277	4.231	4.184	4.135	4.089	4.04	3.988	3.935	3.884
4.344	4.296	4.248	4.198	4.148	4.096	4.041	3.986	3.932
5.551	5.495	5.438	5.378	5.315	5.252	5.187	5.124	5.057
4.802	4.753	4.701	4.648	4.593	4.536	4.478	4.419	4.361
5.236	5.187	5.136	5.084	5.031	4.978	4.919	4.864	4.808
5.378	5.324	5.271	5.217	5.16	5.105	5.049	4.992	4.935
5.441	5.386	5.332	5.276	5.218	5.157	5.1	5.041	4.985
4.5	4.455	4.411	4.367	4.32	4.271	4.221	4.174	4.124
4.508	4.464	4.419	4.375	4.33	4.282	4.234	4.186	4.137
3.882	3.845	3.809	3.772	3.734	3.696	3.654	3.614	3.571
3.71	3.673	3.636	3.598	3.559	3.522	3.482	3.439	3.396
4.368	4.326	4.281	4.235	4.19	4.145	4.099	4.054	4.007
3.713	3.674	3.634	3.596	3.558	3.518	3.476	3.433	3.389
4.702	4.658	4.611	4.564	4.515	4.467	4.417	4.364	4.313
5.308	5.253	5.197	5.139	5.08	5.02	4.955	4.894	4.829
4.804	4.755	4.704	4.653	4.601	4.546	4.487	4.431	4.367
5.937	5.871	5.807	5.737	5.668	5.599	5.529	5.456	5.381
3.889	3.85	3.807	3.766	3.721	3.673	3.625	3.578	3.531
5.248	5.196	5.142	5.082	5.023	4.961	4.898	4.833	4.763
6.157	6.093	6.027	5.959	5.89	5.817	5.744	5.665	5.585
8.786	8.703	8.621	8.535	8.441	8.347	8.25	8.151	8.049
4.492	4.443	4.392	4.34	4.287	4.232	4.176	4.117	4.059
4.491	4.444	4.397	4.347	4.296	4.243	4.191	4.132	4.075
3.858	3.818	3.775	3.731	3.686	3.638	3.592	3.542	3.49

358	360	362	364	366	368	370	372	374
4.661	4.595	4.528	4.46	4.39	4.321	4.249	4.175	4.103
5.875	5.798	5.718	5.635	5.553	5.465	5.378	5.285	5.191
3.344	3.298	3.251	3.206	3.157	3.107	3.058	3.006	2.956
3.827	3.774	3.719	3.662	3.605	3.548	3.487	3.425	3.364
6.992	6.898	6.802	6.707	6.612	6.509	6.401	6.296	6.187
4.597	4.535	4.469	4.402	4.334	4.263	4.192	4.12	4.041
4.143	4.088	4.031	3.972	3.912	3.853	3.794	3.735	3.67
4.572	4.508	4.439	4.371	4.3	4.229	4.158	4.085	4.009
5.005	4.934	4.864	4.795	4.727	4.659	4.588	4.514	4.44
4.921	4.857	4.789	4.722	4.653	4.58	4.505	4.429	4.354
3.633	3.586	3.542	3.498	3.45	3.397	3.343	3.285	3.223
5.11	5.04	4.967	4.892	4.817	4.737	4.659	4.58	4.499
3.92	3.863	3.804	3.747	3.687	3.626	3.565	3.5	3.435
4.476	4.416	4.356	4.292	4.224	4.16	4.09	4.021	3.951
4.588	4.516	4.438	4.357	4.272	4.185	4.096	4.004	3.91
5.373	5.298	5.22	5.14	5.062	4.984	4.901	4.819	4.733
3.834	3.78	3.726	3.668	3.611	3.553	3.493	3.429	3.364
3.877	3.82	3.762	3.701	3.642	3.583	3.522	3.456	3.392
4.991	4.921	4.849	4.779	4.707	4.633	4.556	4.483	4.404
4.303	4.242	4.181	4.117	4.055	3.99	3.92	3.851	3.78
4.753	4.697	4.64	4.579	4.518	4.454	4.39	4.323	4.255
4.881	4.821	4.762	4.699	4.636	4.572	4.508	4.443	4.373
4.928	4.869	4.81	4.752	4.692	4.632	4.566	4.503	4.439
4.073	4.024	3.971	3.917	3.861	3.803	3.744	3.683	3.621
4.089	4.038	3.988	3.938	3.885	3.831	3.776	3.717	3.659
3.531	3.49	3.451	3.408	3.363	3.319	3.273	3.229	3.184
3.355	3.313	3.272	3.228	3.183	3.136	3.09	3.04	2.993
3.959	3.91	3.859	3.811	3.759	3.708	3.652	3.597	3.541
3.347	3.304	3.261	3.217	3.171	3.123	3.075	3.026	2.975
4.261	4.211	4.16	4.107	4.052	3.996	3.939	3.881	3.82
4.763	4.695	4.629	4.56	4.491	4.42	4.346	4.27	4.188
4.307	4.245	4.182	4.119	4.058	3.994	3.926	3.859	3.791
5.308	5.232	5.154	5.078	5.002	4.92	4.838	4.752	4.666
3.482	3.433	3.383	3.335	3.283	3.232	3.18	3.127	3.071
4.697	4.63	4.564	4.494	4.424	4.353	4.279	4.204	4.13
5.506	5.426	5.346	5.266	5.183	5.095	5.005	4.92	4.833
7.944	7.837	7.729	7.626	7.518	7.408	7.294	7.18	7.057
4.003	3.942	3.881	3.82	3.758	3.697	3.632	3.567	3.502
4.018	3.958	3.901	3.843	3.782	3.721	3.66	3.596	3.526
3.44	3.39	3.338	3.284	3.232	3.178	3.126	3.07	3.013

376	378	380	382	384	386	388	390	392
4.024	3.944	3.866	3.783	3.698	3.609	3.517	3.42	3.324
5.094	4.995	4.89	4.786	4.683	4.574	4.457	4.339	4.217
2.902	2.847	2.791	2.737	2.679	2.622	2.562	2.502	2.441
3.3	3.234	3.168	3.098	3.03	2.962	2.892	2.821	2.749
6.071	5.947	5.824	5.703	5.576	5.446	5.312	5.178	5.048
3.96	3.88	3.8	3.716	3.632	3.547	3.461	3.375	3.292
3.604	3.532	3.457	3.385	3.31	3.24	3.162	3.086	3.011
3.929	3.85	3.763	3.677	3.59	3.497	3.407	3.315	3.222
4.364	4.284	4.201	4.119	4.033	3.944	3.855	3.757	3.664
4.276	4.195	4.116	4.036	3.954	3.863	3.777	3.687	3.594
3.159	3.093	3.019	2.95	2.889	2.827	2.764	2.696	2.622
4.415	4.328	4.24	4.148	4.058	3.964	3.861	3.76	3.656
3.367	3.298	3.226	3.155	3.081	3.001	2.919	2.835	2.75
3.879	3.805	3.73	3.65	3.571	3.488	3.4	3.313	3.224
3.812	3.715	3.611	3.513	3.411	3.305	3.201	3.098	3
4.643	4.554	4.461	4.368	4.27	4.169	4.063	3.955	3.843
3.297	3.23	3.156	3.081	3.003	2.924	2.843	2.763	2.679
3.325	3.258	3.187	3.113	3.037	2.963	2.882	2.802	2.719
4.323	4.24	4.152	4.063	3.968	3.87	3.774	3.673	3.573
3.706	3.63	3.554	3.476	3.394	3.304	3.217	3.128	3.038
4.184	4.114	4.044	3.973	3.903	3.832	3.762	3.695	3.627
4.302	4.232	4.162	4.092	4.019	3.949	3.88	3.808	3.735
4.37	4.303	4.234	4.163	4.088	4.02	3.948	3.879	3.81
3.56	3.498	3.436	3.373	3.313	3.25	3.189	3.13	3.076
3.602	3.539	3.477	3.415	3.352	3.29	3.228	3.166	3.107
3.132	3.082	3.032	2.981	2.931	2.881	2.832	2.785	2.739
2.942	2.89	2.839	2.789	2.737	2.684	2.633	2.582	2.534
3.481	3.424	3.363	3.307	3.25	3.193	3.135	3.079	3.026
2.926	2.873	2.819	2.767	2.714	2.663	2.613	2.565	2.514
3.76	3.697	3.634	3.569	3.503	3.439	3.376	3.316	3.257
4.105	4.026	3.944	3.86	3.772	3.684	3.592	3.496	3.398
3.718	3.646	3.573	3.5	3.421	3.341	3.259	3.171	3.087
4.578	4.491	4.402	4.307	4.209	4.103	3.999	3.895	3.788
3.014	2.954	2.892	2.83	2.767	2.704	2.638	2.57	2.498
4.054	3.979	3.896	3.811	3.728	3.642	3.549	3.449	3.346
4.74	4.644	4.545	4.448	4.34	4.226	4.112	3.993	3.876
6.938	6.815	6.679	6.543	6.399	6.26	6.109	5.943	5.774
3.435	3.366	3.297	3.222	3.146	3.07	2.992	2.912	2.829
3.46	3.389	3.321	3.247	3.171	3.089	3.005	2.921	2.833
2.958	2.9	2.841	2.782	2.719	2.653	2.583	2.514	2.442

394	396	398	400	402	404	406	408	410
3.225	3.124	3.028	2.934	2.839	2.748	2.66	2.576	2.501
4.097	3.976	3.856	3.733	3.608	3.492	3.384	3.286	3.197
2.38	2.313	2.247	2.187	2.124	2.067	2.011	1.958	1.911
2.679	2.609	2.539	2.47	2.407	2.35	2.292	2.243	2.197
4.915	4.783	4.656	4.534	4.419	4.31	4.213	4.122	4.044
3.208	3.129	3.046	2.968	2.895	2.829	2.767	2.711	2.657
2.931	2.853	2.776	2.7	2.626	2.551	2.485	2.424	2.368
3.133	3.043	2.957	2.873	2.794	2.72	2.652	2.593	2.541
3.575	3.486	3.399	3.313	3.23	3.153	3.084	3.02	2.962
3.503	3.415	3.331	3.244	3.161	3.086	3.019	2.952	2.894
2.55	2.473	2.393	2.315	2.233	2.157	2.09	2.031	1.982
3.547	3.443	3.338	3.235	3.137	3.04	2.954	2.873	2.801
2.666	2.582	2.5	2.417	2.331	2.251	2.179	2.109	2.042
3.136	3.044	2.953	2.862	2.777	2.696	2.62	2.549	2.485
2.903	2.81	2.723	2.641	2.56	2.486	2.42	2.358	2.304
3.734	3.621	3.513	3.403	3.298	3.202	3.113	3.029	2.951
2.594	2.508	2.427	2.345	2.264	2.189	2.121	2.059	2.004
2.639	2.559	2.477	2.398	2.324	2.255	2.191	2.131	2.077
3.474	3.372	3.273	3.175	3.084	2.994	2.911	2.835	2.766
2.946	2.854	2.759	2.667	2.576	2.49	2.408	2.337	2.268
3.562	3.498	3.441	3.387	3.336	3.287	3.242	3.201	3.161
3.666	3.602	3.538	3.478	3.419	3.364	3.314	3.265	3.221
3.744	3.68	3.617	3.558	3.502	3.449	3.402	3.355	3.313
3.027	2.974	2.925	2.879	2.836	2.794	2.758	2.724	2.692
3.047	2.99	2.933	2.876	2.825	2.777	2.73	2.691	2.653
2.695	2.65	2.606	2.564	2.527	2.492	2.461	2.432	2.402
2.488	2.443	2.399	2.357	2.32	2.285	2.251	2.22	2.194
2.975	2.925	2.878	2.834	2.794	2.752	2.717	2.684	2.652
2.468	2.423	2.378	2.338	2.3	2.264	2.231	2.201	2.172
3.198	3.137	3.083	3.026	2.978	2.931	2.887	2.847	2.809
3.302	3.199	3.093	2.984	2.875	2.776	2.681	2.59	2.505
2.995	2.905	2.809	2.719	2.624	2.53	2.436	2.345	2.261
3.682	3.574	3.465	3.358	3.253	3.155	3.06	2.966	2.88
2.422	2.348	2.272	2.195	2.118	2.038	1.961	1.885	1.815
3.245	3.14	3.035	2.927	2.818	2.709	2.604	2.503	2.406
3.756	3.631	3.511	3.389	3.268	3.148	3.034	2.927	2.826
5.596	5.414	5.222	5.033	4.86	4.688	4.513	4.346	4.195
2.742	2.654	2.569	2.482	2.391	2.306	2.221	2.141	2.066
2.744	2.653	2.558	2.462	2.369	2.281	2.197	2.118	2.042
2.372	2.295	2.216	2.141	2.064	1.987	1.914	1.842	1.776

412	414	416	418	420	422	424	426	428
2.431	2.371	2.319	2.271	2.228	2.189	2.157	2.128	2.103
3.118	3.045	2.978	2.922	2.87	2.823	2.78	2.743	2.712
1.866	1.826	1.791	1.756	1.728	1.702	1.678	1.656	1.637
2.155	2.118	2.082	2.05	2.021	1.997	1.973	1.953	1.935
3.973	3.909	3.851	3.798	3.749	3.704	3.666	3.629	3.596
2.613	2.574	2.536	2.503	2.472	2.444	2.419	2.398	2.376
2.317	2.269	2.23	2.193	2.163	2.133	2.107	2.084	2.065
2.494	2.452	2.415	2.382	2.352	2.325	2.301	2.28	2.262
2.908	2.861	2.818	2.78	2.745	2.715	2.688	2.662	2.638
2.841	2.792	2.75	2.709	2.673	2.644	2.615	2.588	2.566
1.937	1.894	1.856	1.822	1.79	1.757	1.732	1.711	1.691
2.735	2.676	2.622	2.58	2.537	2.501	2.47	2.441	2.415
1.982	1.928	1.879	1.837	1.799	1.767	1.738	1.712	1.69
2.428	2.373	2.328	2.287	2.249	2.221	2.196	2.172	2.152
2.255	2.212	2.171	2.138	2.109	2.081	2.057	2.035	2.017
2.881	2.82	2.765	2.716	2.673	2.634	2.599	2.568	2.542
1.952	1.906	1.867	1.832	1.802	1.775	1.751	1.731	1.712
2.032	1.988	1.949	1.916	1.885	1.858	1.835	1.816	1.797
2.703	2.645	2.595	2.551	2.512	2.478	2.447	2.418	2.393
2.208	2.153	2.105	2.063	2.025	1.991	1.963	1.936	1.913
3.125	3.093	3.065	3.035	3.008	2.981	2.957	2.935	2.912
3.181	3.147	3.111	3.08	3.052	3.023	2.999	2.976	2.955
3.274	3.237	3.205	3.175	3.146	3.12	3.093	3.07	3.046
2.662	2.635	2.613	2.589	2.566	2.542	2.524	2.506	2.49
2.618	2.587	2.56	2.532	2.507	2.484	2.463	2.442	2.425
2.378	2.352	2.33	2.309	2.288	2.27	2.254	2.237	2.222
2.166	2.144	2.123	2.103	2.083	2.066	2.048	2.032	2.017
2.623	2.597	2.572	2.548	2.525	2.505	2.487	2.468	2.449
2.145	2.12	2.099	2.081	2.062	2.044	2.028	2.011	1.997
2.774	2.741	2.71	2.685	2.659	2.637	2.615	2.595	2.576
2.43	2.363	2.302	2.246	2.198	2.154	2.114	2.079	2.047
2.178	2.104	2.03	1.968	1.912	1.866	1.823	1.783	1.749
2.802	2.734	2.67	2.614	2.562	2.515	2.473	2.436	2.401
1.749	1.686	1.631	1.579	1.532	1.49	1.453	1.42	1.392
2.313	2.234	2.161	2.092	2.032	1.979	1.934	1.891	1.853
2.737	2.654	2.582	2.517	2.459	2.409	2.362	2.32	2.282
4.052	3.923	3.8	3.694	3.6	3.519	3.449	3.384	3.327
1.994	1.929	1.869	1.816	1.77	1.73	1.694	1.661	1.632
1.97	1.906	1.847	1.795	1.75	1.711	1.676	1.647	1.62
1.715	1.656	1.603	1.557	1.516	1.48	1.447	1.417	1.391

430	432	434	436	438	440	442	444	446
2.081	2.06	2.042	2.026	2.01	1.996	1.983	1.971	1.961
2.684	2.657	2.633	2.61	2.593	2.575	2.557	2.541	2.526
1.62	1.606	1.593	1.58	1.569	1.558	1.549	1.539	1.531
1.918	1.903	1.889	1.877	1.866	1.855	1.842	1.832	1.823
3.565	3.537	3.511	3.487	3.467	3.444	3.423	3.403	3.387
2.358	2.341	2.325	2.311	2.298	2.283	2.271	2.259	2.245
2.046	2.029	2.013	1.998	1.987	1.974	1.962	1.952	1.941
2.244	2.23	2.215	2.202	2.191	2.178	2.167	2.155	2.144
2.619	2.6	2.583	2.567	2.552	2.539	2.526	2.513	2.501
2.545	2.524	2.507	2.489	2.472	2.458	2.444	2.432	2.418
1.673	1.659	1.64	1.627	1.619	1.611	1.603	1.595	1.588
2.392	2.372	2.353	2.336	2.32	2.307	2.294	2.281	2.269
1.677	1.653	1.638	1.624	1.612	1.601	1.59	1.582	1.572
2.135	2.12	2.106	2.093	2.082	2.071	2.061	2.05	2.041
2.001	1.986	1.973	1.96	1.947	1.937	1.925	1.915	1.905
2.516	2.494	2.474	2.455	2.44	2.423	2.408	2.395	2.381
1.696	1.683	1.67	1.659	1.65	1.642	1.63	1.622	1.614
1.78	1.764	1.751	1.738	1.728	1.716	1.706	1.696	1.688
2.37	2.351	2.332	2.316	2.303	2.288	2.275	2.264	2.251
1.894	1.875	1.858	1.844	1.831	1.819	1.809	1.799	1.789
2.892	2.871	2.853	2.834	2.816	2.796	2.777	2.76	2.742
2.936	2.915	2.895	2.875	2.857	2.839	2.823	2.805	2.789
3.024	3.003	2.983	2.964	2.945	2.925	2.908	2.891	2.871
2.473	2.457	2.44	2.425	2.408	2.393	2.38	2.365	2.35
2.407	2.39	2.375	2.362	2.349	2.334	2.322	2.309	2.295
2.208	2.193	2.179	2.166	2.152	2.14	2.128	2.115	2.102
2.004	1.991	1.978	1.963	1.951	1.939	1.926	1.915	1.905
2.432	2.415	2.399	2.383	2.369	2.353	2.338	2.323	2.31
1.982	1.967	1.954	1.94	1.929	1.915	1.903	1.891	1.879
2.557	2.54	2.522	2.507	2.49	2.474	2.459	2.445	2.431
2.019	1.995	1.971	1.952	1.934	1.917	1.904	1.892	1.879
1.719	1.691	1.665	1.644	1.626	1.608	1.594	1.58	1.569
2.373	2.344	2.32	2.297	2.279	2.26	2.243	2.227	2.213
1.368	1.346	1.326	1.308	1.292	1.279	1.264	1.255	1.247
1.819	1.79	1.764	1.741	1.72	1.702	1.685	1.67	1.658
2.25	2.222	2.196	2.172	2.152	2.133	2.119	2.102	2.088
3.277	3.234	3.198	3.166	3.135	3.11	3.086	3.065	3.046
1.607	1.583	1.562	1.543	1.526	1.513	1.501	1.488	1.479
1.596	1.575	1.558	1.54	1.525	1.513	1.502	1.492	1.482
1.368	1.347	1.329	1.314	1.3	1.288	1.277	1.268	1.259

448	450	452	454	456	458	460	462	464
1.949	1.939	1.926	1.916	1.904	1.895	1.885	1.875	1.865
2.513	2.499	2.485	2.472	2.459	2.447	2.434	2.42	2.409
1.523	1.513	1.505	1.498	1.489	1.481	1.474	1.466	1.459
1.812	1.801	1.792	1.783	1.773	1.763	1.755	1.747	1.738
3.368	3.351	3.333	3.315	3.3	3.283	3.266	3.25	3.232
2.232	2.221	2.208	2.197	2.185	2.175	2.165	2.154	2.144
1.931	1.923	1.911	1.901	1.893	1.884	1.875	1.865	1.857
2.134	2.123	2.113	2.104	2.094	2.085	2.075	2.066	2.057
2.488	2.477	2.466	2.454	2.443	2.432	2.419	2.409	2.398
2.405	2.394	2.382	2.368	2.357	2.345	2.335	2.323	2.312
1.58	1.571	1.559	1.547	1.542	1.536	1.529	1.524	1.517
2.259	2.246	2.234	2.222	2.211	2.199	2.189	2.177	2.166
1.564	1.554	1.546	1.539	1.531	1.523	1.514	1.505	1.498
2.031	2.022	2.013	2.003	1.994	1.986	1.977	1.966	1.958
1.896	1.886	1.878	1.869	1.861	1.853	1.844	1.836	1.827
2.369	2.356	2.344	2.332	2.319	2.307	2.295	2.284	2.272
1.606	1.597	1.588	1.58	1.572	1.564	1.557	1.548	1.541
1.678	1.668	1.66	1.651	1.642	1.634	1.626	1.618	1.61
2.241	2.229	2.218	2.206	2.194	2.185	2.175	2.163	2.154
1.778	1.77	1.759	1.751	1.741	1.734	1.725	1.715	1.708
2.725	2.708	2.692	2.674	2.658	2.641	2.626	2.611	2.595
2.773	2.757	2.743	2.727	2.711	2.696	2.681	2.667	2.654
2.855	2.836	2.819	2.801	2.786	2.769	2.754	2.739	2.724
2.336	2.323	2.31	2.297	2.284	2.271	2.259	2.253	2.237
2.284	2.27	2.257	2.247	2.234	2.222	2.21	2.2	2.188
2.09	2.079	2.068	2.055	2.044	2.031	2.02	2.01	1.998
1.893	1.881	1.869	1.859	1.848	1.837	1.827	1.816	1.807
2.295	2.281	2.268	2.253	2.241	2.227	2.214	2.2	2.186
1.868	1.855	1.845	1.835	1.823	1.812	1.803	1.79	1.781
2.417	2.403	2.389	2.376	2.361	2.349	2.337	2.322	2.311
1.868	1.858	1.847	1.839	1.829	1.82	1.812	1.802	1.795
1.558	1.549	1.538	1.53	1.521	1.513	1.504	1.496	1.488
2.199	2.185	2.174	2.161	2.149	2.138	2.126	2.116	2.104
1.237	1.229	1.222	1.214	1.208	1.2	1.193	1.187	1.181
1.646	1.635	1.624	1.616	1.606	1.596	1.587	1.578	1.57
2.075	2.064	2.05	2.038	2.027	2.015	2.005	1.994	1.982
3.027	3.009	2.993	2.977	2.961	2.945	2.931	2.916	2.902
1.468	1.458	1.45	1.44	1.432	1.424	1.416	1.407	1.399
1.472	1.465	1.456	1.448	1.439	1.431	1.422	1.415	1.406
1.249	1.243	1.235	1.229	1.219	1.213	1.205	1.198	1.192

466	468	470	472	474	476	478	480	482
1.855	1.845	1.836	1.827	1.819	1.809	1.8	1.791	1.783
2.396	2.384	2.372	2.362	2.353	2.34	2.328	2.318	2.309
1.451	1.443	1.436	1.43	1.422	1.414	1.407	1.401	1.394
1.728	1.72	1.711	1.704	1.696	1.687	1.679	1.67	1.664
3.217	3.202	3.186	3.172	3.157	3.143	3.128	3.115	3.102
2.133	2.122	2.112	2.102	2.092	2.081	2.073	2.063	2.055
1.849	1.841	1.832	1.824	1.817	1.808	1.801	1.792	1.785
2.048	2.038	2.029	2.022	2.012	2.006	1.998	1.989	1.982
2.388	2.378	2.367	2.356	2.345	2.337	2.326	2.318	2.309
2.298	2.29	2.279	2.268	2.257	2.248	2.239	2.228	2.217
1.51	1.504	1.495	1.484	1.473	1.468	1.463	1.459	1.456
2.156	2.147	2.135	2.124	2.114	2.104	2.095	2.087	2.077
1.489	1.482	1.476	1.469	1.461	1.454	1.448	1.44	1.434
1.951	1.943	1.935	1.927	1.918	1.911	1.904	1.895	1.888
1.818	1.812	1.804	1.797	1.789	1.782	1.776	1.768	1.763
2.261	2.25	2.239	2.228	2.217	2.207	2.196	2.186	2.177
1.534	1.525	1.52	1.511	1.506	1.499	1.492	1.486	1.479
1.601	1.594	1.585	1.577	1.57	1.563	1.556	1.547	1.542
2.143	2.132	2.124	2.113	2.104	2.095	2.085	2.077	2.068
1.699	1.691	1.682	1.675	1.666	1.659	1.652	1.642	1.635
2.579	2.565	2.55	2.536	2.52	2.507	2.495	2.481	2.468
2.639	2.624	2.611	2.598	2.584	2.573	2.559	2.547	2.535
2.708	2.691	2.677	2.663	2.649	2.635	2.621	2.608	2.596
2.221	2.208	2.2	2.19	2.177	2.165	2.154	2.143	2.134
2.175	2.166	2.155	2.145	2.134	2.124	2.114	2.103	2.095
1.987	1.976	1.966	1.954	1.943	1.935	1.924	1.915	1.907
1.796	1.787	1.777	1.768	1.759	1.75	1.741	1.732	1.723
2.174	2.164	2.15	2.139	2.13	2.117	2.106	2.096	2.084
1.77	1.76	1.75	1.741	1.73	1.721	1.711	1.704	1.694
2.298	2.286	2.274	2.262	2.25	2.239	2.228	2.217	2.207
1.787	1.777	1.769	1.76	1.753	1.745	1.737	1.73	1.722
1.48	1.471	1.464	1.456	1.448	1.442	1.434	1.426	1.419
2.092	2.082	2.072	2.061	2.05	2.038	2.028	2.018	2.008
1.174	1.167	1.16	1.154	1.148	1.142	1.136	1.13	1.123
1.56	1.55	1.541	1.532	1.526	1.517	1.509	1.499	1.492
1.972	1.961	1.952	1.942	1.934	1.922	1.912	1.902	1.893
2.886	2.871	2.857	2.843	2.828	2.815	2.802	2.786	2.773
1.391	1.384	1.376	1.368	1.359	1.351	1.346	1.338	1.33
1.401	1.393	1.386	1.377	1.372	1.363	1.356	1.349	1.342
1.185	1.176	1.17	1.163	1.157	1.15	1.146	1.138	1.132

484	486	488	490	492	494	496	498	500
1.774	1.766	1.759	1.751	1.743	1.735	1.729	1.722	1.714
2.297	2.287	2.276	2.269	2.258	2.249	2.239	2.23	2.223
1.388	1.381	1.376	1.37	1.363	1.358	1.351	1.347	1.342
1.656	1.649	1.642	1.635	1.629	1.622	1.617	1.609	1.604
3.088	3.075	3.062	3.05	3.04	3.026	3.015	3.004	2.993
2.047	2.038	2.029	2.02	2.013	2.006	1.998	1.99	1.982
1.779	1.77	1.764	1.758	1.751	1.744	1.738	1.733	1.725
1.974	1.968	1.96	1.952	1.946	1.939	1.934	1.927	1.919
2.3	2.291	2.282	2.274	2.266	2.258	2.25	2.242	2.234
2.209	2.199	2.19	2.181	2.172	2.164	2.155	2.145	2.139
1.448	1.441	1.435	1.428	1.417	1.412	1.407	1.405	1.403
2.068	2.059	2.05	2.04	2.033	2.026	2.017	2.008	2
1.428	1.422	1.415	1.409	1.402	1.397	1.391	1.387	1.381
1.881	1.874	1.867	1.862	1.854	1.848	1.842	1.836	1.83
1.755	1.748	1.743	1.736	1.731	1.725	1.72	1.714	1.709
2.168	2.157	2.149	2.14	2.13	2.121	2.112	2.104	2.097
1.473	1.468	1.461	1.455	1.449	1.442	1.437	1.432	1.427
1.535	1.529	1.521	1.516	1.51	1.504	1.497	1.492	1.486
2.06	2.05	2.042	2.034	2.026	2.018	2.01	2.002	1.996
1.628	1.621	1.615	1.608	1.601	1.595	1.588	1.583	1.577
2.456	2.444	2.431	2.421	2.41	2.4	2.388	2.377	2.367
2.524	2.512	2.501	2.491	2.48	2.467	2.46	2.45	2.439
2.593	2.571	2.56	2.546	2.535	2.524	2.512	2.502	2.492
2.126	2.117	2.109	2.101	2.094	2.084	2.074	2.066	2.059
2.086	2.078	2.068	2.059	2.051	2.041	2.035	2.027	2.018
1.898	1.888	1.88	1.874	1.864	1.856	1.848	1.84	1.834
1.714	1.707	1.697	1.689	1.684	1.676	1.668	1.661	1.655
2.075	2.064	2.055	2.044	2.036	2.027	2.02	2.011	2.002
1.685	1.676	1.668	1.662	1.654	1.647	1.639	1.63	1.625
2.198	2.186	2.177	2.168	2.159	2.15	2.141	2.132	2.123
1.716	1.708	1.701	1.695	1.688	1.681	1.675	1.669	1.662
1.411	1.405	1.396	1.39	1.385	1.379	1.372	1.366	1.362
1.999	1.99	1.98	1.971	1.961	1.953	1.944	1.937	1.929
1.118	1.111	1.107	1.102	1.096	1.091	1.086	1.082	1.076
1.486	1.478	1.471	1.463	1.457	1.449	1.442	1.435	1.429
1.884	1.875	1.867	1.857	1.848	1.842	1.834	1.827	1.819
2.759	2.747	2.735	2.724	2.712	2.7	2.689	2.679	2.667
1.323	1.317	1.31	1.304	1.298	1.29	1.285	1.28	1.273
1.336	1.331	1.323	1.316	1.312	1.306	1.301	1.296	1.291
1.127	1.12	1.115	1.111	1.105	1.099	1.094	1.089	1.084

502	504	506	508	510	512	514	516	518
1.707	1.7	1.695	1.688	1.682	1.676	1.669	1.664	1.658
2.216	2.206	2.199	2.191	2.183	2.176	2.169	2.162	2.154
1.337	1.333	1.327	1.324	1.317	1.314	1.309	1.305	1.301
1.597	1.592	1.587	1.581	1.576	1.571	1.565	1.56	1.553
2.983	2.971	2.961	2.952	2.941	2.929	2.921	2.911	2.901
1.975	1.966	1.961	1.954	1.947	1.94	1.934	1.928	1.922
1.721	1.715	1.709	1.703	1.697	1.691	1.687	1.681	1.676
1.914	1.909	1.902	1.896	1.89	1.885	1.879	1.874	1.868
2.228	2.221	2.214	2.207	2.201	2.194	2.188	2.181	2.175
2.132	2.124	2.116	2.11	2.102	2.095	2.088	2.082	2.075
1.399	1.395	1.39	1.385	1.379	1.371	1.365	1.361	1.358
1.995	1.987	1.978	1.972	1.965	1.96	1.953	1.946	1.94
1.375	1.372	1.366	1.362	1.358	1.351	1.347	1.343	1.338
1.824	1.819	1.814	1.807	1.803	1.799	1.794	1.788	1.783
1.705	1.699	1.695	1.689	1.684	1.68	1.675	1.67	1.667
2.089	2.081	2.076	2.066	2.061	2.053	2.046	2.04	2.034
1.423	1.418	1.413	1.409	1.404	1.4	1.396	1.389	1.385
1.481	1.475	1.47	1.466	1.462	1.457	1.453	1.448	1.444
1.988	1.981	1.975	1.969	1.963	1.957	1.95	1.945	1.939
1.572	1.566	1.56	1.554	1.549	1.544	1.539	1.535	1.529
2.357	2.347	2.338	2.329	2.319	2.309	2.302	2.295	2.285
2.429	2.419	2.411	2.403	2.393	2.385	2.376	2.368	2.361
2.482	2.472	2.462	2.453	2.442	2.434	2.424	2.417	2.407
2.052	2.044	2.036	2.029	2.023	2.015	2.007	2.003	1.997
2.011	2.004	1.996	1.988	1.981	1.975	1.968	1.961	1.956
1.827	1.82	1.812	1.807	1.8	1.794	1.787	1.782	1.775
1.648	1.641	1.636	1.63	1.623	1.618	1.613	1.607	1.601
1.993	1.987	1.979	1.971	1.965	1.956	1.948	1.942	1.935
1.618	1.61	1.605	1.598	1.593	1.587	1.581	1.574	1.57
2.116	2.107	2.098	2.091	2.083	2.075	2.068	2.062	2.054
1.657	1.652	1.645	1.641	1.636	1.63	1.625	1.62	1.615
1.356	1.35	1.345	1.339	1.333	1.329	1.323	1.32	1.315
1.922	1.913	1.906	1.896	1.891	1.883	1.878	1.872	1.866
1.072	1.068	1.063	1.058	1.054	1.05	1.046	1.042	1.039
1.422	1.417	1.411	1.404	1.401	1.394	1.389	1.383	1.379
1.811	1.803	1.797	1.791	1.783	1.778	1.772	1.765	1.758
2.656	2.645	2.636	2.625	2.617	2.607	2.597	2.59	2.581
1.269	1.263	1.257	1.253	1.248	1.242	1.238	1.234	1.229
1.284	1.279	1.274	1.269	1.264	1.259	1.255	1.25	1.246
1.078	1.075	1.07	1.066	1.062	1.058	1.054	1.05	1.046

520	522	524	526	528	530	532	534	536
1.653	1.647	1.643	1.637	1.633	1.629	1.623	1.619	1.615
2.147	2.142	2.136	2.129	2.122	2.117	2.112	2.105	2.1
1.297	1.293	1.29	1.286	1.282	1.278	1.273	1.271	1.268
1.549	1.545	1.54	1.536	1.531	1.525	1.52	1.518	1.513
2.892	2.884	2.875	2.866	2.857	2.85	2.842	2.834	2.826
1.916	1.909	1.903	1.898	1.892	1.886	1.88	1.876	1.869
1.671	1.666	1.662	1.657	1.653	1.65	1.644	1.64	1.637
1.863	1.858	1.854	1.848	1.842	1.838	1.834	1.828	1.824
2.169	2.164	2.157	2.151	2.146	2.142	2.137	2.131	2.125
2.069	2.062	2.057	2.049	2.044	2.039	2.034	2.029	2.022
1.354	1.35	1.348	1.345	1.342	1.338	1.333	1.329	1.324
1.933	1.928	1.924	1.918	1.912	1.906	1.903	1.897	1.892
1.334	1.33	1.326	1.323	1.32	1.316	1.312	1.307	1.304
1.779	1.774	1.77	1.766	1.76	1.756	1.753	1.749	1.746
1.661	1.658	1.653	1.649	1.645	1.64	1.637	1.632	1.628
2.028	2.02	2.014	2.009	2.002	1.997	1.991	1.985	1.981
1.381	1.379	1.375	1.371	1.367	1.363	1.36	1.357	1.352
1.44	1.433	1.43	1.427	1.423	1.418	1.414	1.41	1.406
1.935	1.928	1.923	1.917	1.911	1.905	1.9	1.896	1.891
1.524	1.52	1.516	1.511	1.507	1.503	1.498	1.495	1.49
2.277	2.271	2.262	2.255	2.248	2.241	2.235	2.229	2.222
2.353	2.346	2.338	2.331	2.325	2.318	2.311	2.303	2.297
2.4	2.392	2.384	2.377	2.37	2.362	2.356	2.348	2.341
1.988	1.982	1.975	1.971	1.967	1.961	1.955	1.949	1.945
1.949	1.943	1.94	1.933	1.926	1.923	1.916	1.91	1.905
1.768	1.764	1.759	1.754	1.748	1.743	1.739	1.732	1.728
1.596	1.591	1.586	1.582	1.576	1.572	1.566	1.562	1.558
1.93	1.923	1.915	1.91	1.904	1.899	1.894	1.888	1.882
1.564	1.559	1.554	1.55	1.545	1.541	1.537	1.532	1.528
2.048	2.042	2.036	2.03	2.022	2.017	2.012	2.006	2
1.611	1.605	1.601	1.596	1.592	1.588	1.584	1.58	1.575
1.31	1.305	1.302	1.297	1.294	1.289	1.286	1.282	1.278
1.857	1.853	1.847	1.841	1.835	1.83	1.824	1.819	1.815
1.035	1.031	1.029	1.026	1.022	1.019	1.015	1.012	1.01
1.374	1.37	1.365	1.361	1.356	1.351	1.346	1.343	1.34
1.753	1.746	1.739	1.735	1.729	1.726	1.719	1.715	1.71
2.573	2.562	2.554	2.547	2.539	2.532	2.525	2.517	2.511
1.224	1.22	1.216	1.214	1.209	1.205	1.202	1.197	1.196
1.242	1.239	1.235	1.23	1.227	1.223	1.22	1.215	1.211
1.043	1.039	1.035	1.031	1.03	1.027	1.022	1.018	1.016

538	540	542	544	546	548	550	552	554
1.61	1.606	1.603	1.596	1.592	1.589	1.584	1.58	1.577
2.094	2.088	2.084	2.078	2.072	2.068	2.062	2.058	2.052
1.264	1.259	1.257	1.254	1.251	1.247	1.246	1.242	1.238
1.508	1.504	1.5	1.495	1.49	1.488	1.484	1.48	1.476
2.82	2.812	2.803	2.797	2.79	2.783	2.776	2.769	2.76
1.864	1.859	1.855	1.85	1.845	1.84	1.835	1.831	1.825
1.632	1.629	1.624	1.62	1.616	1.612	1.609	1.604	1.602
1.818	1.814	1.811	1.806	1.802	1.798	1.793	1.788	1.783
2.119	2.115	2.109	2.104	2.1	2.095	2.09	2.087	2.083
2.016	2.011	2.006	2.001	1.997	1.991	1.986	1.981	1.975
1.321	1.32	1.317	1.314	1.31	1.305	1.301	1.298	1.296
1.888	1.883	1.878	1.872	1.868	1.863	1.858	1.854	1.848
1.299	1.296	1.295	1.291	1.287	1.286	1.28	1.278	1.274
1.741	1.738	1.733	1.729	1.726	1.723	1.719	1.716	1.713
1.623	1.62	1.616	1.612	1.607	1.604	1.6	1.595	1.591
1.975	1.971	1.965	1.961	1.955	1.95	1.945	1.94	1.936
1.349	1.347	1.343	1.339	1.336	1.333	1.329	1.326	1.324
1.402	1.4	1.397	1.393	1.389	1.386	1.381	1.377	1.373
1.886	1.881	1.877	1.873	1.867	1.863	1.858	1.854	1.851
1.485	1.483	1.479	1.475	1.471	1.467	1.464	1.46	1.457
2.214	2.209	2.202	2.196	2.19	2.185	2.179	2.174	2.169
2.291	2.286	2.279	2.273	2.266	2.259	2.255	2.248	2.243
2.334	2.326	2.322	2.315	2.308	2.303	2.297	2.29	2.284
1.939	1.934	1.928	1.922	1.916	1.911	1.907	1.903	1.899
1.9	1.895	1.889	1.885	1.881	1.877	1.872	1.867	1.863
1.724	1.718	1.713	1.71	1.704	1.699	1.695	1.691	1.687
1.554	1.549	1.545	1.542	1.538	1.534	1.531	1.528	1.522
1.878	1.872	1.868	1.863	1.859	1.853	1.85	1.844	1.839
1.522	1.518	1.514	1.51	1.506	1.502	1.499	1.494	1.491
1.996	1.99	1.984	1.979	1.973	1.969	1.965	1.96	1.955
1.571	1.568	1.563	1.56	1.556	1.553	1.549	1.545	1.542
1.276	1.272	1.269	1.265	1.262	1.258	1.255	1.253	1.249
1.809	1.804	1.799	1.794	1.79	1.785	1.781	1.776	1.772
1.006	1.003	1.002	0.998	0.996	0.994	0.99	0.988	0.987
1.335	1.332	1.328	1.324	1.32	1.316	1.313	1.309	1.306
1.706	1.702	1.696	1.691	1.687	1.682	1.678	1.675	1.671
2.505	2.499	2.491	2.485	2.48	2.472	2.465	2.46	2.453
1.192	1.188	1.185	1.18	1.177	1.173	1.172	1.168	1.165
1.21	1.205	1.202	1.199	1.198	1.194	1.191	1.187	1.183
1.014	1.011	1.007	1.005	1.002	1	0.997	0.995	0.992

556	558	560	562	564	566	568	570	572
1.573	1.569	1.565	1.562	1.557	1.555	1.551	1.547	1.543
2.048	2.043	2.039	2.034	2.028	2.023	2.021	2.016	2.011
1.235	1.231	1.229	1.226	1.223	1.22	1.218	1.214	1.212
1.473	1.469	1.465	1.461	1.457	1.455	1.451	1.449	1.445
2.756	2.749	2.744	2.737	2.73	2.725	2.718	2.711	2.706
1.821	1.817	1.813	1.809	1.804	1.799	1.796	1.794	1.79
1.597	1.594	1.59	1.587	1.584	1.581	1.576	1.573	1.569
1.78	1.776	1.774	1.769	1.764	1.76	1.757	1.753	1.751
2.078	2.073	2.067	2.065	2.059	2.056	2.051	2.047	2.042
1.971	1.966	1.962	1.957	1.953	1.948	1.943	1.939	1.937
1.293	1.291	1.288	1.285	1.282	1.279	1.277	1.274	1.27
1.845	1.84	1.836	1.831	1.826	1.823	1.819	1.815	1.81
1.27	1.268	1.266	1.262	1.259	1.256	1.254	1.251	1.248
1.711	1.706	1.701	1.7	1.696	1.693	1.689	1.687	1.683
1.588	1.584	1.58	1.577	1.575	1.571	1.567	1.564	1.561
1.931	1.927	1.922	1.917	1.913	1.907	1.905	1.899	1.895
1.32	1.316	1.313	1.311	1.308	1.304	1.301	1.297	1.296
1.371	1.367	1.364	1.361	1.357	1.354	1.351	1.349	1.345
1.846	1.842	1.838	1.833	1.829	1.824	1.819	1.815	1.812
1.452	1.449	1.445	1.441	1.438	1.435	1.433	1.429	1.426
2.164	2.158	2.153	2.148	2.143	2.138	2.133	2.127	2.123
2.237	2.231	2.227	2.221	2.216	2.211	2.206	2.202	2.196
2.28	2.273	2.269	2.264	2.258	2.252	2.247	2.242	2.237
1.896	1.891	1.887	1.883	1.88	1.873	1.868	1.862	1.859
1.858	1.852	1.849	1.844	1.84	1.837	1.832	1.827	1.823
1.683	1.679	1.675	1.671	1.667	1.663	1.658	1.656	1.653
1.52	1.517	1.513	1.509	1.506	1.504	1.5	1.496	1.493
1.836	1.831	1.827	1.823	1.818	1.814	1.809	1.805	1.802
1.486	1.484	1.481	1.478	1.475	1.472	1.468	1.465	1.461
1.949	1.944	1.94	1.937	1.932	1.928	1.923	1.918	1.915
1.539	1.536	1.533	1.527	1.525	1.521	1.518	1.515	1.511
1.246	1.243	1.24	1.237	1.234	1.231	1.229	1.226	1.223
1.769	1.764	1.759	1.755	1.751	1.745	1.741	1.738	1.733
0.983	0.982	0.977	0.976	0.973	0.972	0.969	0.967	0.964
1.303	1.299	1.297	1.292	1.29	1.287	1.283	1.28	1.277
1.666	1.663	1.659	1.654	1.65	1.646	1.642	1.638	1.634
2.447	2.442	2.434	2.429	2.423	2.417	2.412	2.406	2.402
1.161	1.158	1.157	1.153	1.151	1.147	1.145	1.142	1.14
1.181	1.179	1.176	1.174	1.171	1.167	1.164	1.162	1.159
0.99	0.988	0.985	0.982	0.98	0.977	0.975	0.972	0.97

574	576	578	580	582	584	586	588	590
1.54	1.537	1.533	1.529	1.527	1.523	1.519	1.515	1.512
2.007	2.003	1.998	1.994	1.99	1.987	1.982	1.978	1.974
1.208	1.205	1.202	1.2	1.197	1.194	1.191	1.188	1.186
1.442	1.44	1.436	1.433	1.43	1.428	1.425	1.422	1.42
2.701	2.694	2.689	2.683	2.678	2.673	2.667	2.662	2.658
1.786	1.782	1.779	1.775	1.771	1.768	1.764	1.76	1.758
1.563	1.56	1.557	1.554	1.551	1.549	1.546	1.542	1.539
1.745	1.743	1.738	1.735	1.731	1.728	1.724	1.72	1.716
2.038	2.035	2.031	2.026	2.023	2.021	2.016	2.011	2.008
1.931	1.929	1.923	1.919	1.916	1.91	1.908	1.902	1.899
1.267	1.263	1.261	1.257	1.255	1.253	1.251	1.249	1.246
1.805	1.803	1.798	1.796	1.792	1.788	1.784	1.779	1.775
1.246	1.243	1.24	1.238	1.235	1.232	1.23	1.226	1.225
1.681	1.677	1.675	1.671	1.669	1.665	1.661	1.66	1.657
1.558	1.554	1.551	1.549	1.545	1.542	1.54	1.536	1.533
1.89	1.886	1.882	1.878	1.873	1.87	1.865	1.861	1.857
1.294	1.289	1.288	1.284	1.283	1.281	1.279	1.275	1.273
1.342	1.34	1.336	1.333	1.329	1.326	1.324	1.32	1.318
1.807	1.803	1.801	1.795	1.792	1.79	1.784	1.781	1.778
1.422	1.42	1.416	1.414	1.411	1.407	1.405	1.402	1.398
2.119	2.114	2.11	2.105	2.1	2.096	2.091	2.087	2.085
2.191	2.187	2.182	2.176	2.172	2.167	2.163	2.158	2.152
2.232	2.228	2.222	2.218	2.213	2.209	2.205	2.2	2.195
1.855	1.851	1.864	1.865	1.861	1.859	1.856	1.849	1.845
1.821	1.817	1.813	1.809	1.806	1.801	1.798	1.794	1.792
1.648	1.645	1.64	1.636	1.633	1.629	1.627	1.624	1.62
1.49	1.486	1.484	1.48	1.477	1.473	1.47	1.467	1.466
1.798	1.796	1.792	1.788	1.785	1.782	1.777	1.774	1.77
1.458	1.454	1.452	1.449	1.446	1.444	1.441	1.439	1.435
1.91	1.906	1.902	1.898	1.894	1.891	1.886	1.884	1.88
1.51	1.505	1.502	1.498	1.495	1.493	1.489	1.487	1.482
1.22	1.216	1.214	1.213	1.209	1.205	1.202	1.201	1.198
1.731	1.726	1.723	1.72	1.716	1.711	1.708	1.704	1.7
0.961	0.96	0.959	0.957	0.952	0.951	0.948	0.947	0.945
1.274	1.271	1.268	1.266	1.262	1.26	1.256	1.254	1.251
1.631	1.626	1.623	1.62	1.616	1.612	1.609	1.606	1.602
2.397	2.39	2.387	2.378	2.374	2.369	2.363	2.358	2.354
1.138	1.135	1.132	1.13	1.126	1.125	1.122	1.118	1.117
1.157	1.155	1.151	1.149	1.147	1.143	1.142	1.139	1.138
0.968	0.966	0.963	0.962	0.958	0.957	0.956	0.952	0.95

592	594	596	598	600	602	604	606	608
1.508	1.506	1.501	1.497	1.495	1.492	1.49	1.487	1.484
1.969	1.966	1.961	1.957	1.954	1.95	1.946	1.942	1.938
1.184	1.181	1.179	1.176	1.172	1.171	1.169	1.166	1.162
1.417	1.413	1.412	1.409	1.406	1.404	1.4	1.398	1.396
2.651	2.647	2.642	2.636	2.63	2.625	2.62	2.615	2.611
1.753	1.751	1.749	1.746	1.743	1.74	1.735	1.732	1.727
1.536	1.534	1.53	1.528	1.523	1.522	1.518	1.516	1.512
1.713	1.711	1.707	1.703	1.699	1.695	1.692	1.689	1.685
2.004	2.001	1.995	1.993	1.989	1.987	1.984	1.979	1.974
1.894	1.89	1.886	1.882	1.88	1.876	1.872	1.868	1.864
1.243	1.241	1.239	1.235	1.233	1.23	1.227	1.225	1.223
1.771	1.768	1.764	1.76	1.757	1.753	1.749	1.745	1.742
1.222	1.22	1.217	1.214	1.211	1.208	1.205	1.204	1.201
1.656	1.652	1.649	1.647	1.643	1.64	1.636	1.635	1.631
1.53	1.528	1.524	1.522	1.52	1.517	1.514	1.51	1.508
1.853	1.849	1.846	1.842	1.837	1.833	1.829	1.825	1.822
1.27	1.268	1.264	1.261	1.259	1.254	1.254	1.25	1.248
1.315	1.312	1.309	1.305	1.302	1.301	1.298	1.294	1.292
1.773	1.769	1.765	1.761	1.76	1.756	1.752	1.749	1.745
1.396	1.393	1.39	1.386	1.384	1.381	1.378	1.375	1.371
2.079	2.075	2.07	2.066	2.063	2.059	2.056	2.051	2.047
2.148	2.144	2.139	2.135	2.132	2.128	2.123	2.119	2.114
2.19	2.186	2.183	2.179	2.175	2.169	2.165	2.16	2.156
1.819	1.816	1.814	1.811	1.806	1.802	1.8	1.796	1.789
1.787	1.784	1.78	1.776	1.772	1.769	1.764	1.76	1.757
1.615	1.613	1.609	1.608	1.604	1.599	1.595	1.592	1.589
1.462	1.46	1.456	1.453	1.451	1.448	1.445	1.442	1.44
1.768	1.764	1.76	1.757	1.754	1.749	1.746	1.744	1.74
1.433	1.43	1.428	1.425	1.422	1.419	1.415	1.413	1.41
1.875	1.869	1.866	1.862	1.86	1.856	1.852	1.848	1.843
1.479	1.476	1.474	1.47	1.467	1.465	1.462	1.458	1.455
1.195	1.193	1.19	1.186	1.185	1.183	1.181	1.178	1.175
1.695	1.693	1.689	1.687	1.683	1.679	1.677	1.675	1.669
0.944	0.941	0.939	0.937	0.935	0.933	0.931	0.929	0.927
1.249	1.246	1.243	1.239	1.238	1.234	1.231	1.229	1.227
1.599	1.595	1.592	1.589	1.586	1.582	1.578	1.576	1.572
2.349	2.343	2.338	2.334	2.329	2.325	2.319	2.314	2.307
1.114	1.113	1.111	1.107	1.105	1.102	1.101	1.099	1.095
1.134	1.132	1.13	1.127	1.124	1.122	1.119	1.118	1.115
0.95	0.947	0.944	0.942	0.939	0.937	0.935	0.932	0.931

610	612	614	616	618	620	622	624	626
1.481	1.477	1.473	1.47	1.466	1.464	1.46	1.457	1.453
1.934	1.93	1.926	1.922	1.918	1.915	1.911	1.907	1.905
1.16	1.159	1.155	1.152	1.151	1.147	1.145	1.143	1.14
1.393	1.388	1.386	1.384	1.381	1.378	1.376	1.373	1.371
2.606	2.601	2.595	2.591	2.586	2.58	2.575	2.57	2.566
1.724	1.721	1.717	1.713	1.711	1.708	1.705	1.703	1.7
1.511	1.507	1.505	1.502	1.499	1.497	1.494	1.49	1.488
1.683	1.679	1.675	1.672	1.669	1.665	1.662	1.658	1.656
1.971	1.968	1.965	1.961	1.958	1.954	1.952	1.947	1.945
1.86	1.857	1.852	1.848	1.843	1.841	1.837	1.834	1.829
1.221	1.216	1.215	1.212	1.21	1.207	1.205	1.202	1.2
1.738	1.735	1.731	1.727	1.723	1.718	1.716	1.712	1.709
1.198	1.196	1.194	1.191	1.188	1.187	1.184	1.181	1.179
1.628	1.625	1.622	1.62	1.616	1.614	1.612	1.609	1.606
1.505	1.504	1.499	1.497	1.494	1.492	1.489	1.488	1.484
1.817	1.815	1.81	1.806	1.802	1.8	1.795	1.791	1.789
1.244	1.244	1.241	1.24	1.236	1.234	1.232	1.23	1.226
1.288	1.286	1.283	1.28	1.277	1.274	1.271	1.269	1.266
1.741	1.737	1.734	1.731	1.726	1.723	1.721	1.716	1.714
1.369	1.366	1.364	1.361	1.358	1.356	1.353	1.349	1.346
2.044	2.04	2.035	2.031	2.028	2.024	2.019	2.016	2.012
2.111	2.105	2.101	2.097	2.092	2.09	2.084	2.079	2.075
2.152	2.149	2.145	2.14	2.134	2.131	2.127	2.123	2.121
1.785	1.782	1.78	1.777	1.776	1.771	1.767	1.764	1.76
1.754	1.75	1.748	1.743	1.74	1.738	1.733	1.732	1.73
1.586	1.583	1.581	1.577	1.573	1.571	1.567	1.565	1.563
1.437	1.434	1.43	1.428	1.425	1.423	1.421	1.419	1.418
1.738	1.734	1.731	1.727	1.725	1.72	1.717	1.715	1.711
1.409	1.406	1.403	1.401	1.398	1.395	1.393	1.39	1.388
1.842	1.838	1.834	1.831	1.827	1.823	1.82	1.817	1.814
1.452	1.449	1.446	1.444	1.441	1.438	1.435	1.433	1.43
1.172	1.169	1.166	1.163	1.162	1.159	1.157	1.155	1.151
1.667	1.664	1.66	1.656	1.652	1.65	1.645	1.643	1.64
0.925	0.923	0.92	0.919	0.917	0.915	0.912	0.911	0.908
1.224	1.221	1.218	1.215	1.213	1.211	1.208	1.205	1.202
1.569	1.566	1.562	1.559	1.556	1.552	1.55	1.548	1.544
2.305	2.299	2.295	2.29	2.285	2.281	2.275	2.271	2.267
1.092	1.09	1.088	1.085	1.083	1.082	1.079	1.077	1.074
1.112	1.111	1.107	1.105	1.101	1.099	1.097	1.096	1.093
0.928	0.927	0.925	0.923	0.921	0.918	0.916	0.914	0.912

628	630	632	634	636	638	640	642	644
1.452	1.45	1.446	1.443	1.44	1.437	1.434	1.43	1.429
1.902	1.898	1.895	1.891	1.889	1.885	1.881	1.878	1.875
1.139	1.136	1.135	1.132	1.13	1.128	1.126	1.124	1.122
1.37	1.366	1.365	1.361	1.36	1.357	1.355	1.353	1.35
2.561	2.557	2.553	2.549	2.545	2.541	2.537	2.531	2.529
1.698	1.695	1.692	1.689	1.687	1.684	1.681	1.679	1.676
1.486	1.483	1.481	1.478	1.475	1.473	1.471	1.469	1.465
1.652	1.65	1.645	1.643	1.641	1.637	1.636	1.632	1.627
1.941	1.939	1.935	1.933	1.928	1.926	1.922	1.919	1.916
1.827	1.822	1.819	1.816	1.814	1.81	1.807	1.803	1.8
1.198	1.195	1.194	1.191	1.187	1.186	1.185	1.182	1.179
1.707	1.702	1.699	1.695	1.694	1.688	1.686	1.684	1.68
1.176	1.175	1.172	1.169	1.167	1.166	1.164	1.162	1.16
1.604	1.601	1.599	1.596	1.595	1.592	1.589	1.588	1.585
1.484	1.481	1.478	1.476	1.473	1.472	1.469	1.467	1.463
1.785	1.782	1.779	1.774	1.771	1.767	1.764	1.762	1.759
1.225	1.223	1.219	1.217	1.215	1.212	1.211	1.209	1.206
1.263	1.26	1.259	1.257	1.253	1.25	1.247	1.245	1.242
1.71	1.707	1.705	1.701	1.698	1.694	1.693	1.69	1.686
1.344	1.342	1.34	1.336	1.334	1.332	1.329	1.325	1.325
2.01	2.007	2.003	2.001	1.997	1.993	1.99	1.986	1.982
2.071	2.068	2.065	2.063	2.058	2.055	2.051	2.047	2.043
2.117	2.113	2.11	2.106	2.102	2.097	2.094	2.091	2.087
1.755	1.752	1.749	1.746	1.745	1.743	1.74	1.736	1.733
1.726	1.723	1.721	1.718	1.714	1.71	1.707	1.704	1.701
1.559	1.557	1.553	1.549	1.547	1.545	1.541	1.54	1.537
1.414	1.411	1.409	1.406	1.405	1.402	1.399	1.397	1.395
1.708	1.707	1.704	1.701	1.697	1.694	1.692	1.689	1.687
1.386	1.385	1.382	1.379	1.377	1.374	1.371	1.37	1.367
1.81	1.807	1.805	1.802	1.797	1.795	1.793	1.789	1.786
1.428	1.424	1.422	1.419	1.417	1.414	1.411	1.409	1.406
1.149	1.147	1.145	1.142	1.141	1.137	1.135	1.133	1.131
1.636	1.633	1.629	1.626	1.623	1.62	1.617	1.616	1.613
0.907	0.905	0.902	0.902	0.9	0.896	0.896	0.893	0.892
1.2	1.199	1.195	1.194	1.191	1.189	1.187	1.184	1.182
1.541	1.539	1.535	1.532	1.529	1.527	1.524	1.521	1.519
2.262	2.259	2.254	2.251	2.246	2.242	2.237	2.233	2.229
1.073	1.07	1.067	1.065	1.064	1.061	1.059	1.058	1.055
1.092	1.089	1.088	1.085	1.084	1.08	1.079	1.076	1.074
0.911	0.91	0.907	0.906	0.904	0.903	0.9	0.899	0.898

646	648	650	652	654	656	658	660	662
1.425	1.423	1.42	1.417	1.416	1.412	1.41	1.408	1.405
1.873	1.869	1.865	1.862	1.859	1.857	1.854	1.849	1.846
1.119	1.118	1.115	1.114	1.11	1.11	1.107	1.105	1.103
1.348	1.346	1.345	1.342	1.34	1.337	1.336	1.334	1.332
2.523	2.518	2.513	2.51	2.505	2.501	2.496	2.492	2.489
1.673	1.671	1.668	1.665	1.661	1.66	1.658	1.653	1.651
1.464	1.461	1.458	1.457	1.453	1.451	1.45	1.447	1.444
1.625	1.623	1.62	1.618	1.616	1.612	1.609	1.606	1.603
1.912	1.91	1.906	1.903	1.898	1.897	1.894	1.892	1.888
1.796	1.793	1.79	1.787	1.784	1.78	1.777	1.774	1.77
1.177	1.175	1.173	1.17	1.169	1.166	1.165	1.162	1.159
1.677	1.675	1.675	1.669	1.666	1.663	1.66	1.656	1.653
1.158	1.156	1.153	1.152	1.149	1.148	1.144	1.143	1.14
1.583	1.58	1.579	1.577	1.573	1.57	1.569	1.565	1.563
1.462	1.46	1.457	1.455	1.452	1.452	1.45	1.448	1.445
1.756	1.752	1.749	1.746	1.744	1.739	1.737	1.733	1.73
1.205	1.202	1.2	1.2	1.198	1.196	1.193	1.191	1.189
1.24	1.238	1.234	1.233	1.23	1.229	1.226	1.223	1.221
1.683	1.68	1.676	1.672	1.669	1.667	1.664	1.661	1.659
1.321	1.319	1.317	1.316	1.313	1.31	1.308	1.305	1.304
1.979	1.976	1.972	1.969	1.964	1.963	1.962	1.958	1.955
2.04	2.036	2.032	2.03	2.026	2.023	2.018	2.014	2.012
2.083	2.082	2.077	2.073	2.07	2.066	2.063	2.06	2.056
1.727	1.721	1.72	1.717	1.716	1.714	1.711	1.709	1.705
1.7	1.696	1.693	1.69	1.687	1.685	1.682	1.68	1.677
1.533	1.532	1.529	1.525	1.523	1.521	1.518	1.516	1.513
1.394	1.39	1.389	1.386	1.384	1.382	1.38	1.378	1.375
1.683	1.681	1.677	1.675	1.671	1.669	1.667	1.665	1.662
1.366	1.363	1.361	1.359	1.357	1.354	1.353	1.35	1.349
1.783	1.78	1.778	1.774	1.773	1.769	1.766	1.763	1.76
1.403	1.4	1.398	1.395	1.394	1.39	1.388	1.385	1.382
1.129	1.126	1.125	1.124	1.121	1.119	1.118	1.114	1.113
1.609	1.606	1.605	1.6	1.598	1.594	1.592	1.59	1.586
0.889	0.889	0.887	0.885	0.883	0.881	0.88	0.878	0.877
1.18	1.176	1.174	1.173	1.171	1.169	1.166	1.164	1.162
1.514	1.512	1.508	1.506	1.504	1.5	1.498	1.496	1.493
2.226	2.222	2.215	2.212	2.208	2.204	2.201	2.199	2.194
1.052	1.05	1.049	1.047	1.045	1.042	1.041	1.04	1.038
1.074	1.071	1.069	1.068	1.065	1.064	1.061	1.06	1.059
0.897	0.895	0.891	0.89	0.888	0.887	0.885	0.883	0.883

664	666	668	670	672	674	676	678	680
1.402	1.4	1.397	1.395	1.393	1.389	1.388	1.386	1.384
1.843	1.84	1.838	1.834	1.831	1.828	1.826	1.823	1.82
1.1	1.098	1.095	1.094	1.093	1.091	1.09	1.087	1.085
1.328	1.326	1.324	1.321	1.319	1.317	1.314	1.311	1.31
2.487	2.483	2.478	2.475	2.472	2.468	2.463	2.459	2.456
1.649	1.646	1.644	1.641	1.639	1.636	1.632	1.629	1.627
1.442	1.439	1.436	1.434	1.432	1.43	1.429	1.426	1.423
1.6	1.597	1.595	1.592	1.589	1.588	1.584	1.584	1.58
1.887	1.883	1.88	1.878	1.874	1.871	1.871	1.867	1.863
1.768	1.764	1.761	1.758	1.756	1.752	1.748	1.746	1.742
1.158	1.155	1.155	1.152	1.15	1.149	1.148	1.146	1.142
1.651	1.648	1.647	1.643	1.64	1.637	1.635	1.633	1.63
1.139	1.137	1.134	1.133	1.132	1.129	1.127	1.126	1.124
1.562	1.558	1.557	1.554	1.552	1.549	1.548	1.544	1.544
1.443	1.441	1.438	1.437	1.433	1.432	1.43	1.428	1.428
1.727	1.725	1.721	1.719	1.716	1.713	1.71	1.708	1.705
1.186	1.185	1.182	1.181	1.178	1.177	1.176	1.173	1.171
1.219	1.216	1.214	1.212	1.21	1.207	1.205	1.203	1.2
1.655	1.654	1.649	1.647	1.644	1.642	1.639	1.636	1.634
1.301	1.298	1.297	1.295	1.293	1.291	1.289	1.288	1.285
1.952	1.948	1.946	1.941	1.939	1.936	1.934	1.931	1.928
2.007	2.004	2.001	1.998	1.995	1.993	1.988	1.985	1.984
2.053	2.05	2.046	2.044	2.04	2.037	2.034	2.031	2.027
1.702	1.696	1.694	1.692	1.688	1.686	1.684	1.68	1.678
1.675	1.672	1.669	1.666	1.664	1.661	1.658	1.656	1.652
1.51	1.508	1.504	1.502	1.499	1.497	1.494	1.492	1.49
1.374	1.371	1.369	1.367	1.366	1.365	1.361	1.359	1.357
1.66	1.657	1.655	1.652	1.65	1.648	1.645	1.643	1.639
1.347	1.344	1.342	1.341	1.339	1.335	1.333	1.331	1.33
1.758	1.754	1.751	1.747	1.745	1.743	1.741	1.737	1.735
1.381	1.378	1.377	1.373	1.37	1.37	1.367	1.365	1.363
1.111	1.107	1.106	1.105	1.103	1.101	1.099	1.098	1.097
1.584	1.581	1.579	1.576	1.573	1.57	1.569	1.565	1.562
0.876	0.873	0.872	0.871	0.869	0.868	0.866	0.865	0.864
1.16	1.157	1.155	1.153	1.151	1.148	1.147	1.145	1.144
1.491	1.487	1.485	1.483	1.481	1.478	1.476	1.472	1.47
2.19	2.186	2.183	2.18	2.175	2.172	2.168	2.165	2.16
1.035	1.034	1.032	1.03	1.028	1.027	1.025	1.023	1.021
1.056	1.054	1.052	1.049	1.049	1.047	1.046	1.045	1.043
0.881	0.879	0.878	0.876	0.875	0.873	0.871	0.869	0.869

682	684	686	688	690	692	694	696	698
1.381	1.38	1.377	1.376	1.373	1.372	1.369	1.368	1.364
1.818	1.815	1.812	1.81	1.807	1.806	1.803	1.8	1.798
1.083	1.08	1.079	1.078	1.075	1.074	1.071	1.068	1.068
1.307	1.305	1.303	1.3	1.298	1.297	1.294	1.291	1.29
2.452	2.448	2.446	2.442	2.436	2.434	2.431	2.427	2.423
1.624	1.622	1.618	1.616	1.612	1.611	1.608	1.606	1.604
1.422	1.419	1.418	1.415	1.413	1.411	1.41	1.407	1.405
1.577	1.575	1.573	1.571	1.569	1.565	1.563	1.56	1.559
1.861	1.858	1.856	1.853	1.851	1.849	1.848	1.844	1.84
1.738	1.737	1.733	1.731	1.727	1.726	1.723	1.72	1.717
1.141	1.138	1.136	1.135	1.133	1.133	1.131	1.131	1.128
1.628	1.624	1.621	1.619	1.616	1.613	1.611	1.608	1.604
1.123	1.122	1.119	1.118	1.115	1.113	1.111	1.111	1.108
1.539	1.538	1.536	1.533	1.53	1.528	1.525	1.522	1.52
1.425	1.422	1.421	1.417	1.415	1.413	1.412	1.409	1.407
1.701	1.698	1.697	1.692	1.691	1.688	1.685	1.683	1.68
1.17	1.168	1.168	1.164	1.163	1.161	1.16	1.157	1.155
1.199	1.198	1.195	1.191	1.191	1.19	1.186	1.185	1.181
1.63	1.629	1.628	1.624	1.62	1.618	1.616	1.613	1.61
1.283	1.281	1.279	1.278	1.275	1.273	1.271	1.269	1.267
1.924	1.921	1.918	1.915	1.912	1.91	1.908	1.906	1.903
1.981	1.977	1.974	1.97	1.967	1.964	1.962	1.959	1.956
2.023	2.02	2.018	2.015	2.011	2.008	2.006	2.002	2
1.676	1.673	1.669	1.668	1.665	1.661	1.659	1.657	1.654
1.649	1.646	1.644	1.641	1.639	1.637	1.634	1.63	1.629
1.488	1.484	1.484	1.481	1.479	1.476	1.474	1.472	1.47
1.355	1.354	1.351	1.35	1.347	1.346	1.343	1.342	1.339
1.636	1.634	1.632	1.63	1.628	1.626	1.624	1.621	1.619
1.328	1.327	1.325	1.324	1.322	1.319	1.317	1.318	1.314
1.733	1.729	1.727	1.725	1.722	1.719	1.717	1.714	1.711
1.362	1.358	1.357	1.354	1.352	1.349	1.347	1.346	1.344
1.094	1.093	1.09	1.088	1.088	1.085	1.084	1.082	1.081
1.561	1.558	1.554	1.552	1.55	1.549	1.546	1.545	1.542
0.861	0.86	0.859	0.857	0.857	0.856	0.855	0.852	0.852
1.141	1.14	1.139	1.135	1.135	1.133	1.131	1.129	1.129
1.467	1.465	1.463	1.461	1.459	1.457	1.455	1.452	1.451
2.158	2.155	2.152	2.148	2.144	2.14	2.138	2.134	2.131
1.018	1.018	1.017	1.015	1.013	1.013	1.011	1.009	1.007
1.041	1.037	1.036	1.035	1.033	1.033	1.03	1.029	1.028
0.867	0.867	0.865	0.862	0.861	0.859	0.859	0.857	0.856

700	702	704	706	708	710	712	714	716
1.362	1.361	1.358	1.357	1.354	1.353	1.35	1.349	1.347
1.796	1.795	1.791	1.789	1.787	1.784	1.782	1.779	1.776
1.068	1.066	1.064	1.063	1.063	1.06	1.059	1.056	1.055
1.289	1.285	1.283	1.281	1.279	1.275	1.274	1.271	1.269
2.42	2.415	2.412	2.408	2.405	2.402	2.398	2.394	2.392
1.6	1.597	1.595	1.592	1.589	1.587	1.584	1.581	1.578
1.403	1.401	1.399	1.397	1.395	1.393	1.39	1.389	1.386
1.555	1.552	1.55	1.548	1.546	1.542	1.54	1.536	1.535
1.836	1.835	1.831	1.828	1.827	1.826	1.822	1.821	1.817
1.715	1.712	1.71	1.708	1.705	1.702	1.699	1.696	1.694
1.126	1.125	1.123	1.121	1.119	1.117	1.115	1.112	1.112
1.602	1.6	1.597	1.593	1.592	1.59	1.585	1.584	1.58
1.107	1.105	1.103	1.102	1.1	1.099	1.097	1.095	1.093
1.516	1.513	1.51	1.508	1.503	1.5	1.496	1.492	1.488
1.405	1.403	1.401	1.398	1.397	1.394	1.392	1.39	1.388
1.678	1.676	1.673	1.671	1.669	1.667	1.664	1.661	1.659
1.154	1.153	1.15	1.148	1.146	1.145	1.142	1.142	1.138
1.18	1.178	1.177	1.175	1.173	1.17	1.167	1.167	1.165
1.607	1.604	1.602	1.6	1.596	1.595	1.594	1.59	1.588
1.265	1.262	1.261	1.259	1.257	1.257	1.254	1.253	1.25
1.9	1.898	1.895	1.892	1.889	1.886	1.884	1.882	1.879
1.953	1.949	1.947	1.945	1.943	1.939	1.937	1.933	1.932
1.997	1.994	1.991	1.989	1.986	1.982	1.981	1.977	1.974
1.649	1.646	1.645	1.643	1.64	1.637	1.634	1.632	1.628
1.627	1.624	1.622	1.617	1.616	1.614	1.612	1.61	1.608
1.468	1.466	1.464	1.461	1.459	1.457	1.454	1.452	1.45
1.337	1.334	1.334	1.33	1.33	1.326	1.327	1.325	1.323
1.616	1.613	1.612	1.611	1.608	1.607	1.604	1.603	1.6
1.313	1.31	1.309	1.306	1.303	1.303	1.302	1.299	1.298
1.709	1.707	1.703	1.7	1.698	1.697	1.695	1.693	1.689
1.341	1.34	1.338	1.337	1.335	1.332	1.33	1.33	1.327
1.078	1.077	1.074	1.073	1.071	1.069	1.069	1.069	1.066
1.54	1.537	1.536	1.534	1.533	1.529	1.526	1.525	1.523
0.851	0.849	0.847	0.847	0.845	0.844	0.844	0.841	0.841
1.126	1.125	1.123	1.121	1.119	1.117	1.117	1.115	1.113
1.449	1.447	1.444	1.443	1.44	1.44	1.437	1.435	1.433
2.127	2.125	2.124	2.12	2.118	2.114	2.111	2.108	2.106
1.005	1.003	1.002	1.002	0.998	0.998	0.997	0.995	0.994
1.025	1.024	1.022	1.021	1.021	1.02	1.017	1.016	1.015
0.855	0.854	0.853	0.851	0.851	0.849	0.848	0.845	0.846

718	720	722	724	726	728	730	732	734
1.345	1.342	1.34	1.338	1.337	1.334	1.334	1.332	1.33
1.775	1.774	1.771	1.768	1.767	1.764	1.763	1.76	1.759
1.054	1.053	1.051	1.05	1.048	1.047	1.046	1.043	1.04
1.267	1.266	1.263	1.262	1.259	1.257	1.254	1.252	1.25
2.388	2.385	2.382	2.379	2.375	2.371	2.368	2.363	2.362
1.576	1.572	1.571	1.568	1.565	1.561	1.56	1.557	1.556
1.385	1.383	1.38	1.377	1.377	1.374	1.372	1.37	1.367
1.533	1.529	1.526	1.523	1.521	1.518	1.517	1.514	1.513
1.814	1.812	1.81	1.807	1.804	1.801	1.799	1.798	1.795
1.691	1.69	1.687	1.685	1.681	1.678	1.675	1.673	1.672
1.11	1.108	1.107	1.104	1.102	1.103	1.1	1.1	1.098
1.578	1.576	1.573	1.572	1.568	1.565	1.564	1.561	1.558
1.092	1.09	1.088	1.087	1.085	1.084	1.083	1.081	1.079
1.485	1.48	1.476	1.473	1.469	1.465	1.461	1.457	1.453
1.386	1.385	1.382	1.381	1.378	1.376	1.374	1.372	1.371
1.658	1.655	1.654	1.651	1.647	1.646	1.644	1.64	1.639
1.138	1.136	1.135	1.134	1.133	1.13	1.128	1.127	1.125
1.162	1.16	1.158	1.156	1.155	1.153	1.15	1.149	1.147
1.586	1.584	1.583	1.58	1.577	1.576	1.572	1.57	1.568
1.249	1.246	1.245	1.242	1.24	1.239	1.238	1.237	1.234
1.878	1.875	1.871	1.87	1.867	1.865	1.862	1.86	1.858
1.928	1.926	1.923	1.921	1.919	1.916	1.913	1.911	1.908
1.971	1.97	1.966	1.964	1.961	1.959	1.977	1.971	1.952
1.625	1.623	1.62	1.619	1.616	1.613	1.61	1.609	1.606
1.606	1.605	1.602	1.598	1.596	1.594	1.592	1.59	1.589
1.448	1.446	1.443	1.44	1.44	1.437	1.436	1.434	1.43
1.322	1.32	1.318	1.315	1.314	1.314	1.312	1.309	1.307
1.599	1.596	1.596	1.592	1.589	1.588	1.587	1.585	1.582
1.295	1.294	1.293	1.291	1.29	1.289	1.286	1.285	1.283
1.687	1.685	1.682	1.68	1.679	1.675	1.674	1.672	1.669
1.326	1.324	1.322	1.32	1.318	1.316	1.315	1.313	1.31
1.064	1.062	1.062	1.06	1.058	1.056	1.056	1.054	1.052
1.521	1.518	1.517	1.516	1.513	1.512	1.509	1.506	1.506
0.84	0.84	0.838	0.835	0.835	0.833	0.832	0.832	0.831
1.112	1.11	1.11	1.109	1.107	1.105	1.104	1.103	1.102
1.43	1.429	1.427	1.424	1.422	1.421	1.419	1.416	1.415
2.103	2.1	2.099	2.095	2.092	2.091	2.088	2.087	2.084
0.993	0.991	0.99	0.989	0.987	0.984	0.985	0.983	0.982
1.013	1.012	1.012	1.009	1.008	1.007	1.005	1.003	1.003
0.844	0.842	0.842	0.841	0.84	0.84	0.839	0.838	0.836

736	738	740	742	744	746	748	750	752
1.328	1.326	1.324	1.323	1.322	1.321	1.319	1.318	1.317
1.755	1.754	1.752	1.751	1.748	1.747	1.745	1.743	1.742
1.039	1.039	1.037	1.036	1.035	1.034	1.033	1.032	1.03
1.249	1.247	1.245	1.243	1.241	1.241	1.239	1.237	1.237
2.359	2.356	2.354	2.351	2.348	2.344	2.342	2.34	2.339
1.552	1.55	1.548	1.547	1.545	1.543	1.54	1.537	1.536
1.367	1.367	1.365	1.362	1.361	1.359	1.357	1.357	1.354
1.51	1.507	1.505	1.504	1.502	1.5	1.498	1.496	1.494
1.794	1.791	1.789	1.787	1.785	1.783	1.78	1.779	1.778
1.669	1.667	1.665	1.664	1.661	1.659	1.657	1.655	1.653
1.096	1.095	1.094	1.091	1.09	1.09	1.088	1.087	1.084
1.557	1.555	1.552	1.55	1.549	1.547	1.545	1.544	1.541
1.078	1.076	1.075	1.073	1.071	1.071	1.071	1.068	1.067
1.448	1.443	1.44	1.437	1.43	1.429	1.424	1.421	1.416
1.369	1.368	1.366	1.365	1.363	1.362	1.359	1.359	1.356
1.636	1.635	1.632	1.632	1.631	1.627	1.626	1.623	1.622
1.124	1.122	1.122	1.118	1.118	1.117	1.117	1.114	1.112
1.146	1.145	1.143	1.142	1.138	1.138	1.137	1.135	1.135
1.567	1.566	1.562	1.56	1.558	1.556	1.554	1.552	1.551
1.233	1.231	1.23	1.229	1.228	1.225	1.225	1.223	1.222
1.854	1.852	1.85	1.848	1.847	1.846	1.842	1.842	1.838
1.906	1.904	1.901	1.899	1.896	1.894	1.892	1.891	1.889
1.95	1.947	1.945	1.942	1.941	1.938	1.937	1.934	1.933
1.605	1.602	1.6	1.598	1.594	1.593	1.59	1.589	1.587
1.586	1.585	1.583	1.581	1.579	1.577	1.576	1.574	1.573
1.43	1.427	1.426	1.424	1.422	1.421	1.421	1.418	1.417
1.305	1.305	1.302	1.301	1.3	1.299	1.297	1.296	1.295
1.58	1.579	1.577	1.575	1.573	1.573	1.569	1.568	1.568
1.282	1.281	1.278	1.277	1.275	1.273	1.273	1.271	1.27
1.667	1.666	1.664	1.661	1.659	1.658	1.656	1.655	1.651
1.309	1.306	1.304	1.302	1.3	1.299	1.298	1.296	1.296
1.05	1.05	1.047	1.046	1.045	1.046	1.044	1.043	1.042
1.504	1.502	1.5	1.497	1.496	1.495	1.494	1.492	1.49
0.829	0.829	0.828	0.827	0.825	0.826	0.827	0.824	0.824
1.101	1.1	1.098	1.096	1.095	1.094	1.093	1.093	1.09
1.413	1.414	1.412	1.411	1.408	1.408	1.406	1.405	1.403
2.082	2.079	2.076	2.074	2.073	2.07	2.07	2.067	2.066
0.982	0.981	0.977	0.977	0.975	0.975	0.974	0.974	0.971
1.001	1.001	0.999	0.997	0.996	0.996	0.994	0.993	0.993
0.835	0.833	0.833	0.833	0.831	0.83	0.829	0.828	0.827

754	756	758	760	762	764	766	768	770
1.316	1.314	1.313	1.311	1.309	1.308	1.308	1.305	1.305
1.739	1.74	1.737	1.735	1.733	1.732	1.73	1.728	1.728
1.03	1.028	1.027	1.026	1.025	1.023	1.021	1.021	1.02
1.234	1.233	1.231	1.23	1.229	1.227	1.225	1.223	1.222
2.335	2.331	2.33	2.328	2.326	2.324	2.32	2.318	2.316
1.533	1.532	1.532	1.529	1.528	1.526	1.524	1.522	1.522
1.352	1.35	1.349	1.348	1.347	1.346	1.345	1.343	1.341
1.493	1.491	1.489	1.486	1.484	1.483	1.481	1.48	1.477
1.776	1.775	1.772	1.77	1.768	1.767	1.766	1.763	1.762
1.652	1.651	1.649	1.646	1.645	1.643	1.64	1.639	1.638
1.084	1.082	1.08	1.079	1.079	1.077	1.074	1.075	1.074
1.54	1.537	1.536	1.534	1.533	1.529	1.529	1.528	1.525
1.067	1.065	1.064	1.063	1.061	1.061	1.06	1.059	1.056
1.412	1.409	1.405	1.401	1.398	1.396	1.392	1.389	1.385
1.355	1.354	1.351	1.35	1.349	1.347	1.347	1.346	1.343
1.62	1.619	1.616	1.616	1.615	1.612	1.61	1.61	1.608
1.11	1.11	1.109	1.108	1.106	1.104	1.103	1.102	1.101
1.134	1.133	1.131	1.13	1.129	1.126	1.125	1.123	1.122
1.548	1.547	1.547	1.545	1.543	1.54	1.539	1.538	1.536
1.221	1.219	1.219	1.217	1.215	1.215	1.213	1.213	1.211
1.837	1.835	1.833	1.83	1.828	1.827	1.826	1.825	1.823
1.887	1.884	1.883	1.881	1.88	1.877	1.875	1.873	1.872
1.93	1.927	1.926	1.924	1.922	1.92	1.919	1.916	1.913
1.586	1.582	1.581	1.58	1.577	1.575	1.573	1.57	1.569
1.57	1.57	1.568	1.566	1.565	1.563	1.562	1.559	1.558
1.415	1.414	1.412	1.41	1.409	1.408	1.405	1.405	1.402
1.294	1.292	1.291	1.289	1.289	1.287	1.286	1.285	1.282
1.565	1.564	1.562	1.56	1.559	1.556	1.556	1.555	1.552
1.27	1.269	1.267	1.267	1.265	1.262	1.262	1.26	1.259
1.65	1.65	1.648	1.646	1.644	1.642	1.64	1.639	1.638
1.295	1.294	1.294	1.291	1.29	1.288	1.288	1.287	1.286
1.039	1.04	1.038	1.036	1.036	1.034	1.033	1.034	1.031
1.489	1.486	1.486	1.484	1.482	1.483	1.481	1.477	1.476
0.822	0.821	0.821	0.82	0.819	0.817	0.817	0.816	0.816
1.089	1.088	1.087	1.085	1.085	1.084	1.083	1.081	1.081
1.402	1.401	1.399	1.399	1.396	1.395	1.393	1.392	1.39
2.063	2.062	2.058	2.058	2.055	2.054	2.052	2.05	2.047
0.97	0.969	0.969	0.969	0.966	0.966	0.965	0.965	0.963
0.992	0.991	0.989	0.988	0.987	0.985	0.985	0.985	0.983
0.827	0.825	0.826	0.825	0.824	0.823	0.821	0.82	0.82

772	774	776	778	780	782	784	786	788
1.302	1.301	1.3	1.298	1.298	1.297	1.296	1.293	1.292
1.727	1.725	1.723	1.722	1.72	1.719	1.716	1.715	1.714
1.019	1.018	1.016	1.015	1.015	1.013	1.013	1.012	1.011
1.22	1.219	1.219	1.217	1.215	1.215	1.214	1.213	1.21
2.313	2.311	2.308	2.305	2.304	2.302	2.3	2.298	2.296
1.52	1.518	1.516	1.514	1.512	1.51	1.509	1.508	1.506
1.34	1.339	1.338	1.336	1.335	1.334	1.334	1.331	1.33
1.476	1.474	1.472	1.47	1.468	1.468	1.465	1.465	1.463
1.759	1.758	1.755	1.754	1.754	1.751	1.75	1.748	1.747
1.636	1.635	1.632	1.631	1.629	1.628	1.626	1.624	1.622
1.072	1.071	1.07	1.07	1.067	1.066	1.063	1.063	1.062
1.524	1.522	1.521	1.519	1.518	1.517	1.514	1.513	1.512
1.055	1.054	1.053	1.052	1.051	1.051	1.05	1.048	1.047
1.383	1.38	1.377	1.373	1.372	1.368	1.367	1.364	1.363
1.342	1.341	1.339	1.335	1.335	1.334	1.333	1.333	1.331
1.607	1.606	1.603	1.602	1.6	1.598	1.596	1.596	1.593
1.101	1.098	1.098	1.096	1.095	1.094	1.092	1.092	1.089
1.121	1.119	1.118	1.117	1.115	1.114	1.113	1.111	1.11
1.534	1.532	1.531	1.529	1.527	1.525	1.524	1.523	1.52
1.21	1.209	1.207	1.205	1.203	1.203	1.201	1.2	1.199
1.821	1.819	1.816	1.815	1.813	1.811	1.811	1.808	1.806
1.869	1.867	1.865	1.864	1.862	1.86	1.858	1.855	1.854
1.912	1.91	1.909	1.907	1.904	1.903	1.9	1.899	1.897
1.568	1.566	1.565	1.562	1.561	1.558	1.556	1.553	1.553
1.557	1.555	1.553	1.551	1.55	1.547	1.547	1.546	1.543
1.401	1.4	1.397	1.396	1.394	1.393	1.392	1.39	1.388
1.282	1.28	1.278	1.278	1.277	1.275	1.275	1.273	1.271
1.551	1.551	1.548	1.546	1.544	1.544	1.541	1.541	1.54
1.258	1.255	1.255	1.254	1.251	1.25	1.25	1.248	1.247
1.636	1.634	1.632	1.631	1.63	1.628	1.626	1.623	1.623
1.286	1.284	1.282	1.282	1.28	1.28	1.278	1.277	1.276
1.031	1.03	1.028	1.027	1.027	1.026	1.025	1.024	1.023
1.476	1.474	1.473	1.467	1.468	1.465	1.462	1.461	1.461
0.815	0.815	0.813	0.812	0.812	0.812	0.81	0.809	0.808
1.08	1.079	1.077	1.076	1.075	1.075	1.073	1.072	1.071
1.389	1.387	1.385	1.384	1.383	1.383	1.381	1.38	1.378
2.046	2.043	2.041	2.04	2.039	2.035	2.034	2.032	2.03
0.963	0.962	0.961	0.961	0.959	0.959	0.957	0.954	0.955
0.984	0.981	0.98	0.979	0.978	0.977	0.977	0.976	0.974
0.819	0.819	0.817	0.816	0.815	0.814	0.813	0.813	0.813

790	792	794	796	798	800
1.292	1.29	1.287	1.288	1.286	1.285
1.712	1.71	1.708	1.707	1.706	1.703
1.009	1.009	1.008	1.007	1.006	1.005
1.209	1.209	1.207	1.205	1.203	1.202
2.293	2.291	2.289	2.287	2.284	2.281
1.505	1.503	1.501	1.499	1.498	1.496
1.328	1.327	1.326	1.324	1.323	1.322
1.46	1.458	1.456	1.456	1.453	1.449
1.745	1.743	1.74	1.739	1.737	1.735
1.621	1.619	1.617	1.615	1.613	1.611
1.06	1.058	1.058	1.058	1.055	1.054
1.51	1.508	1.505	1.504	1.502	1.5
1.045	1.044	1.043	1.043	1.041	1.039
1.361	1.359	1.356	1.354	1.353	1.35
1.33	1.329	1.327	1.327	1.325	1.325
1.592	1.591	1.588	1.586	1.585	1.583
1.089	1.086	1.086	1.085	1.084	1.082
1.11	1.108	1.106	1.106	1.103	1.101
1.519	1.515	1.514	1.512	1.511	1.51
1.198	1.196	1.195	1.194	1.193	1.191
1.804	1.802	1.799	1.799	1.797	1.795
1.851	1.849	1.848	1.847	1.844	1.843
1.895	1.894	1.892	1.89	1.888	1.886
1.55	1.549	1.547	1.546	1.543	1.541
1.542	1.541	1.54	1.539	1.535	1.535
1.388	1.386	1.384	1.382	1.381	1.38
1.27	1.269	1.267	1.266	1.265	1.263
1.539	1.536	1.535	1.533	1.532	1.529
1.246	1.244	1.243	1.242	1.241	1.239
1.62	1.619	1.617	1.616	1.613	1.612
1.275	1.273	1.272	1.271	1.27	1.267
1.022	1.02	1.02	1.019	1.018	1.017
1.459	1.458	1.458	1.455	1.454	1.453
0.808	0.807	0.805	0.804	0.803	0.804
1.069	1.069	1.067	1.067	1.065	1.064
1.377	1.375	1.375	1.373	1.371	1.37
2.028	2.026	2.024	2.023	2.021	2.019
0.954	0.952	0.95	0.95	0.949	0.947
0.975	0.972	0.973	0.971	0.971	0.969
0.812	0.811	0.811	0.809	0.808	0.807
